# Individual and combined effects of *GSTM1*, *GSTT1*, and *GSTP1* polymorphisms on breast cancer risk: A meta-analysis and re-analysis of systematic meta-analyses

**DOI:** 10.1371/journal.pone.0216147

**Published:** 2020-03-10

**Authors:** Li-Feng Miao, Xiang-Hua Ye, Xiao-Feng He

**Affiliations:** 1 Department of Galactophore, Heping Hospital Affiliated to Changzhi Medical College, Shanxi, Changzhi, China; 2 Department of Radiotherapy, First Affiliated Hospital, Zhejiang University School of Medicine, Hangzhou, China; 3 Department of Science and Education, Heping Hospital Affiliated to Changzhi Medical College, Shanxi, Changzhi, China; Istituto di Ricovero e Cura a Carattere Scientifico Centro di Riferimento Oncologico della Basilicata, ITALY

## Abstract

**Background:**

Fourteen previous meta-analyses have been published to analyze the polymorphisms of individual *GSTM1* present/null, *GSTT1* present/null, and *GSTP1* IIe105Val on breast cancer (BC) risk. However, their meta-analyses did not explore the combined effects of the three genetic polymorphisms on BC risk. In addition, they did not evaluate the credibility of statistically significant associations. Furthermore, a multitude of new articles have been published on these themes, and therefore a meta-analysis and re-analysis of systematic previous meta-analyses were performed to further explore these issues.

**Objectives:**

To determine the association between the individual and combined effects of *GSTM1*, *GSTT1*, and *GSTP1* polymorphisms on breast cancer risk.

**Methods:**

Crude odds ratios (ORs) and their 95% confidence intervals (CIs) were applied to estimate the association between individual and combined effects of *GSTM1*, *GSTT1*, and *GSTP1* polymorphisms on BC risk. To evaluate the credibility of statistically significant associations in the current and previous meta-analyses, we applied the the false-positive report probabilities (FPRP) test and the Venice criteria.

**Results:**

101 publications were selected to evaluate the individual and combined effects of *GSTM1*, *GSTT1* and *GSTP1* polymorphisms on BC risk. Overall, statistically significant elevated BC risk was found in any individual and combined effects of *GSTM1* present/null, *GSTT1* present/null, and *GSTP1* IIe105Val polymorphisms. However, when we restricted studies only involving with high-quality, matching, HWE, and genotyping examination performed blindly or with quality control, significantly increased BC risk was only found in overall population for *GSTM1* null genotype, among all populations, Caucasians, and postmenopausal women for the combined effects of *GSTM1* and *GSTT1* polymorphisms, and in overall analysis for the combined effects of *GSTM1*, *GSTT1*, and *GSTP1* IIe105Val polymorphisms. Further, less-credible positive results were identified when we evaluated the credibility of positive results of the current and previous meta-analyses.

**Conclusions:**

This meta-analysis indicates that the individual and combined effects of *GSTM1*, *GSTT1* and *GSTP1* polymorphisms may be not associated with increased BC risk.

## Introduction

Breast cancer (BC) is one of the most common diseases and an important public health challenge among women worldwide, although the incidences of BC are not the same in different countries and ethnic groups [1, 2]. Risk factors that have been confirmed including age, family history and several reproductive factors only explain one-third of BC cases [3]. Studies on the pathologic mechanism of BC remain enigmatic, and it is a multifactorial and polygenic disease which may be influenced by both environmental and genetic factors [4, 5]. Therefore, studies on gene polymorphisms have become much more important in the progression of BC worldwide [6, 7].

In recent years, some genes have been confirmed as potential cancer susceptible genes. Glutathione S-transferases (*GSTs*) are overwhelmingly important genes, which play key role in the detoxification of toxic, potentially carcinogenic compounds, and a host of basic physiological processes of the human body [8–11]. In human, five classes of *GST* enzymes have been found (α,μ,π,σ, andθ) [12] and each class is encoded by an independent gene or family genes (such as *GSTA*, *GSTM*, *GSTP*, *GSTO*, and *GSTT* genes). Among these genes, both *GSTM1* and *GSTT1* genes show deletion polymorphisms (null genotype) [13, 14], which cause the absence of expression and enzyme activity loss [15]. They are located on chromosome 1 (1p13.3) and chromosome 22 (22q11.2), respectively [16]. An codon 105 A to G mutation at exon 5 in *GSTP1* polymorphism results in a change isoleucine (IIe) to valine (Val), which also decreases enzymatic activity [17, 18]. Therefore, the three gene mutations may increase BC risk on the basis of their biological effects.

In 1993, the first publication was reported on the association between *GSTM1* null genotype and BC cancer susceptibility [Reference 1 in S1 Appendix]. The first study investigated the association between individual *GSTT1* null genotype and the combined effects of *GSTM1* present/null, *GSTT1* present/null, and *GSTP1* IIe105Val polymorphisms on BC cancer risk was published in 1998 [Reference 5 in S1 Appendix], and it is the first article that was published to explore the association between *GSTP1* IIe105Val polymorphism and BC cancer risk [Reference 110 in S1 Appendix]. So far, 116 publications [References 1–116 in S1 Appendix] have been reported on these themes. Nevertheless, the results of these studies were contradictory. Fourteen previous meta-analyses [19–32] have been published to analyze the individual *GSTM1* present/null, *GSTT1* present/null, and *GSTP1* IIe105Val polymorphisms on BC risk. However, their meta-analyses did not conduct the combined effects of the three genes on BC risk, in addition, they did not evaluate the credibility of statistically significant associations, furthermore, a lot of new studies have been published, and therefore a meta-analysis and re-analysis of previous meta-analyses were carried out to further explore the individual and combined effects of these genes on BC risk.

## Materials and methods

### Search strategy

Literature search was performed using PubMed and CNKI databases in this meta-analysis (update to 18 May, 2018). The following search strategy was applied: (glutathione S-transferase T1 OR *GSTT1* OR glutathione S-transferase P1 OR *GSTP1* OR glutathione S-transferase M1 OR *GSTM1*) AND breast AND (polymorphism OR genotype OR allele OR variant OR mutation). Language was not restricted in the present meta-analysis. It was implemented to identify additional studies manually (references of the original and review studies). Finally, the corresponding authors were contacted via e-mail if necessary.

### Inclusion and exclusion criteria

The eligible publications were selected applying the following criteria: (1) case–control study; (2) detailed genotype frequencies were afforded between case and control groups; (3) studies must assess the association between the individual and combined effects of *GSTM1* present/null, *GSTT1* present/null and *GSTP1* IIe105Val polymorphisms on BC risk. Studies were removed if they were case reports, duplicate data or incomplete data, meta-analysis, and so on.

### Data extraction

Information was carefully collected independently by two investigators from all selected studies. Potential disagreements were judged through the corresponding authors if necessary. The following information was collected: first author’s surname, year of publication, country, ethnicity, source of cases, source of controls, type of controls, matching, single nucleotide polymorphism (SNP), sample size, and genotype frequencies of the individual and combined effects of *GSTM1*, *GSTT1* and *GSTP1* polymorphisms on BC risk.

### Quality score assessment

The quality of the studies were appraised independently by two of all authors. We designed quality assessment criteria on the basis of two previous meta-analyses [33, 34]. S1 Table lists the scale for quality assessment of molecular association studies of BC. S3 and S4 Tables list the quality assessment by included studies of *GSTM1* present/null, *GSTT1* present/null, and *GSTP1* IIe105Val polymorphisms with BC risk. They were considered as low quality studies if quality scores were ≤10, while scores of > 10 were regarded as high quality in this meta-analysis.

### Statistical analysis

We applied crude odds ratios (ORs) and their 95% confidence intervals (CIs) to estimate the association between individual and combined effects of *GSTM1* present/null, *GSTT1* present/null, and *GSTP1* IIe105Val polymorphisms on BC risk. We used null vs. present model to calculate the pooled ORs with their 95% CIs for the *GSTM1* present/null and *GSTT1* present/null polymorphisms. Analysis was conducted employing the following genetic models for *GSTP1* IIe105Val polymorphism: Val/Val vs. IIe/IIe, IIe/Val vs. IIe/IIe, Val/Val vs. IIe/IIe + IIe/Val, Val/Val + IIe/Val vs. IIe/IIe, and Val vs. IIe. For the combined effects of *GSTM1* present/null and *GSTT1* present/null polymorphisms, we applied the following genetic models: + *−* vs. + +, *−* + vs. + +, *− −* vs. + +, (+ *−*) + (*−* +) vs. + +, (− −) + (+ *−*) + (*−* +) vs. + +, and *− −* vs. (+ +) + (+ *−*) + (*−* +). *− −* was *GSTM1* null/*GSTT1* null, + + was *GSTM1* present/*GSTT1* present, + *−* was *GSTM1* present/*GSTT1* null, and *−* + was *GSTM1* null/*GSTT1* present. The following genetic models were used for the combined effects of *GSTM1* present/null and *GSTP1* IIe105Val polymorphisms: *GSTM1* null/*GSTP1* IIe/IIe vs. *GSTM1* present/*GSTP1* IIe/IIe, *GSTM1* present/*GSTP1* Val* vs. *GSTM1* present/*GSTP1* IIe/IIe, all one high risk genotypes vs. *GSTM1* present/*GSTP1* IIe/IIe, *GSTM1* null/*GSTP1* Val* vs. *GSTM1* present/*GSTP1* IIe/IIe, all high risk genotypes vs. *GSTM1* present/*GSTP1* IIe/IIe, and *GSTM1* null/*GSTP1* Val* vs. (*GSTM1* null/*GSTP1* IIe/IIe + *GSTM1* present/*GSTP1* Val* + *GSTM1* present/*GSTP1* IIe/IIe). For the combined effects of *GSTT1* present/null and *GSTP1* IIe105Val polymorphisms, the following genetic models were employed: *GSTT1* null/*GSTP1* IIe/IIe vs. *GSTT1* present/*GSTP1* IIe/IIe, *GSTT1* present/*GSTP1* Val* vs. *GSTT1* present/*GSTP1* IIe/IIe, all one high risk genotypes vs. *GSTT1* present/*GSTP1* IIe/IIe, *GSTT1* null/*GSTP1* Val* vs. *GSTT1* present/*GSTP1* IIe/IIe, all high risk genotypes vs. *GSTT1* present/*GSTP1* IIe/IIe, and *GSTT1* null/*GSTP1* Val* vs. (*GSTT1* null/*GSTP1* IIe/IIe + *GSTT1* present/*GSTP1* Val* + *GSTT1* present/*GSTP1* IIe/IIe). Finally, for the combined effects of *GSTM1* present/null, *GSTT1* present/null, and *GSTP1* IIe105Val polymorphisms, we applied the following ten genetic models: *GSTM1* null/*GSTT1* present/*GSTP1* IIe/IIe vs. *GSTM1* present/*GSTT1* present/*GSTP1* IIe/IIe, *GSTM1* present/*GSTT1* null/*GSTP1* IIe/IIe vs. *GSTM1* present/*GSTT1* present/*GSTP1* IIe/IIe, *GSTM1* present/*GSTT1* present/*GSTP1* Val* vs. *GSTM1* present/*GSTT1* present/*GSTP1* IIe/IIe, all one high-risk genotypes vs. *GSTM1* present/*GSTT1* present/*GSTP1* IIe/IIe, *GSTM1* null/*GSTT1* null/*GSTP1* IIe/IIe vs. *GSTM1* present/*GSTT1* present/*GSTP1* IIe/IIe, *GSTM1* null/*GSTT1* present/*GSTP1* Val* vs. *GSTM1* present/*GSTT1* present/*GSTP1* IIe/IIe, *GSTM1* present/*GSTT1* null/*GSTP1* Val* vs. *GSTM1* present/*GSTT1* present/*GSTP1* IIe/IIe, all two high-risk genotypes vs. *GSTM1* present/*GSTT1* present/*GSTP1* IIe/IIe, *GSTM1* null/*GSTT1* null//*GSTP1* Val* vs. *GSTM1* present/*GSTT1* present/*GSTP1* IIe/IIe, and *GSTM1* null/*GSTT1* null//*GSTP1* Val* vs. (all one high-risk genotypes + all two high-risk genotypes + *GSTM1* present/*GSTT1* present/*GSTP1* IIe/IIe). We employed *Q* test to evaluate heterogeneity among selected studies. A statistically significant heterogeneity was regarded if *P* < 0.10 and *I*^*2*^ > 50% [35]. A fixed-effects model [36] was considered if the heterogeneity was not notable, if not, a random-effects model was used [37]. Subgroup analyses were conducted on the basis of ethnicity, source of controls, type of controls, sample size, quality score, matching, menopausal status, smoking habits, and Hardy-Weinberg equilibrium (HWE). Chi-square goodness-of-fit test was applied to check HWE, and significant deviation was considered in control groups if *P* < 0.05. Heterogeneity sources were estimated according to a meta-regression analysis method. A sensitivity analysis was performed by using two methods: first, a single study was removed each time, second, a dataset was used that the comprised only high-quality studies, matching studies, HWE, and genotyping performed blindly or with quality control [38]. Publication bias was confirmed on the basis of Begg’s funnel plot [39] Egger’s test (significant publication bias was considered if *P* < 0.05) [40]. A nonparametric ‘trim and fill’ method was applied to accredit missing studies [41] if publication bias was observed. To evaluate the credibility of statistically significant associations in the current and previous meta-analyses, we applied the false-positive report probabilities (FPRP) test [42] and the Venice criteria [43]. The FPRP was estimated using an Excel spreadsheet S2 Appendix. All statistical analyses were calculated using STATA version 9.0 (STATA Corporation, College Station, TX).

## Results

### Study characteristics

**Fig 1:**
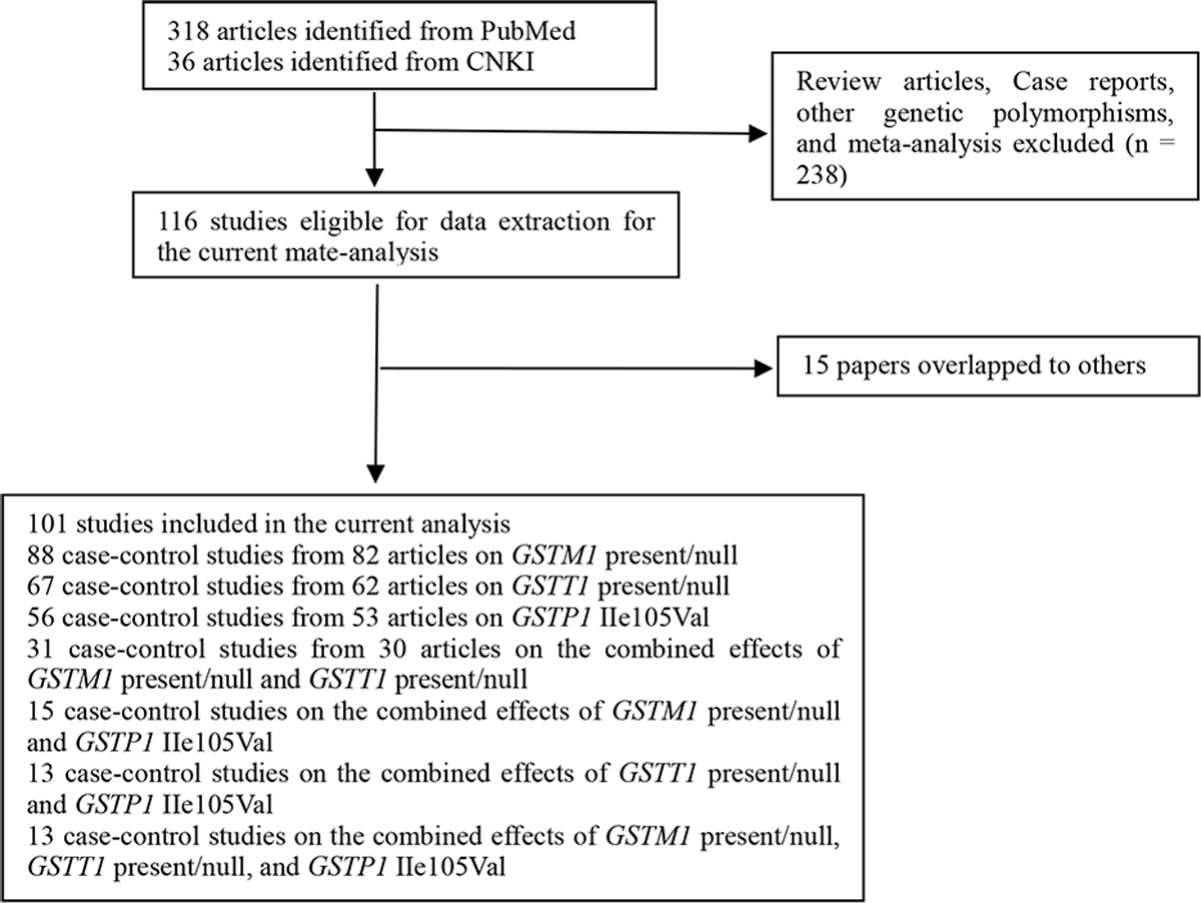
Flow diagram for identifying and including studies in the current meta-analysis.

Fig 1 lists the flow diagram for identifying and including studies in the current meta-analysis. 354 titles met the search criteria. 238 articles were excluded because they were review articles, case reports, other genes, and meta-analyses. In addition, fifteen articles [References 2, 4, 10, 21, 31, 32, 33, 35, 45, 54, 76, 82, 91, 107, 115 in S1 Appendix] were removed because their sample had been overlapped with another eleven studies [8, 9, 17, 23, 41, 47, 55, 64, 71, 88, 105 in S1 Appendix]. In the end, 101 publications were selected to evaluate the individual and combined effects of *GSTM1* present/null, *GSTT1* present/null, and *GSTP1* IIe105Val polymorphisms on BC risk. S2 Table shows the general characteristics of studies included in this meta-analysis. There were 88 case–control studies from 82 publications on *GSTM1* present/null polymorphism (involving 28,676 BC cases and 32,539 controls, S5 Table), 67 case–control studies from 62 publications on *GSTT1* present/null polymorphism (involving 23,092 BC cases and 26,381 controls, S5 Table), 56 case–control studies from 53 articles on *GSTP1* IIe105Val polymorphism (involving 25,331 BC cases and 27,424 controls, S5 Table), 31 case–control studies from 30 articles on the combined effects of both *GSTM1* and *GSTT1* null genotypes (involving 10,497 BC cases and 10,242 controls, S8 Table), 15 case–control studies on the combined effects of *GSTM1* present/null and *GSTP1* IIe105Val polymorphisms (involving 6,272 BC cases and 6,739 controls, S10 Table), 13 case–control studies on the combined effects of *GSTT1* present/null and *GSTP1* IIe105Val polymorphisms (involving 5,413 BC cases and 5,567 controls, S11 Table), and 13 case–control studies on the combined effects of three *GSTM1* present/null, *GSTT1* present/null, and *GSTP1* IIe105Val polymorphisms (involving 5,395 BC cases and 5,544 controls, S12 Table). In addition, twenty, fifteen, ten, and seven case–control studies were conducted to analyze *GSTM1* null genotype (including 7,934 BC cases and 11,059 controls), *GSTT1* null genotype (including 6,786 BC cases and 9,477 controls), *GSTP1* IIe105Val (including 3,448 BC cases and 3,676 controls), and the combined effects of *GSTM1* and *GSTT1* polymorphisms (including 1,916 BC cases and 2,268 controls) among postmenopausal women, and seventeen, twelve, fifteen, and six case–control studies were conducted to analyze *GSTM1* null genotype (including 2,840 BC cases and 3,393 controls), *GSTT1* null genotype (including 1,605 BC cases and 1,830 controls), *GSTP1* IIe105Val (including 8,493 BC cases and 11,040 controls), and the combined effects of *GSTM1* and *GSTT1* polymorphisms (including 981 BC cases and 1,185 controls) among premenopausal women, respectively, as shown in S6–S9 Tables. Furthermore, there were five, three, and zero current smoking studies, seven, six, and one past smoking studies, and eleven, nine, and three no-smoking studies on *GSTM1*, *GSTT1*, and *GSTP1* polymorphisms, respectively, as shown in S7 Table. Finally, there were 31 high-quality studies and 57 low-quality studies on *GSTM1* present/null, 23 high-quality studies and 44 low-quality studies on *GSTT1* present/null, 30 high-quality studies and 26 low-quality studies on *GSTP1* IIe105Val, 13 high-quality studies and 18 low-quality studies on the combined effects of *GSTM1* and *GSTT1*, nine high-quality studies and six low-quality studies on the combined effects of *GSTM1* and *GSTP1*, eight high-quality studies and five low-quality studies on the combined effects of *GSTT1* and *GSTP1*, and eight high-quality studies and five low-quality studies on the combined effects of *GSTM1*, *GSTT1*, and *GSTP1* polymorphisms as determined by quality assessment of molecular association studies (S1 Table).

### Quantitative synthesis

At the overall analysis, the *GSTM1* null genotype was associated with elevated BC risk (OR = 1.12, 95% CI = 1.06–1.09). In addition, significantly elevated BC risk was also observed in a slice of subgroups, such as Asians, population-based studies, healthy women, cancer-free women, cancer-free patients, matching studies, no matching studies, large-sized studies, small-sized studies, high-quality studies, low-quality studies, postmenopausal and premenopausal women, as shown in Table 1.

**Table 1:** Meta-analysis of the association of *GSTM1* polymorphism with risk of breast cancer.

Variable	n	Cases/Controls	Test of association	Test of heterogeneity	
			OR (95% CI)	*P*_h_	*I*^2^ (%)
Overall	88	28,676/32,539	1.12 (1.06–1.19)*	<0.001	59.9
Ethnicity					
African	6	1,177/1,171	0.92 (0.77–1.10)	0.964	0.0
Indian	7	1,597/1,702	1.36 (0.95–1.95)*	<0.001	78.5
Asian	19	5,690/7,536	1.20 (1.05–1.38)*	<0.001	66.5
Caucasian	37	13,357/15,573	1.05 (0.97–1.13)*	0.001	48.3
Source of controls					
HB	41	7,561/7,956	1.07 (0.96–1.19)*	<0.001	55.1
PB	28	17,240/21,204	1.15 (1.06–1.25)*	<0.001	69.1
Type of controls					
Healthy women	28	6,436/7,087	1.21 (1.06–1.39)*	<0.001	67.7
Cancer-free women	47	20,264/23,502	1.07 (1.00–1.13)*	<0.001	46.6
Cancer-free patients	8	893/863	1.50 (1.05–2.14)*	0.007	63.7
Matching					
Yes	46	19,528/23,342	1.07 (1.02–1.13)*	0.039	28.7
No	42	9,148/9,197	1.19 (1.05–1.34)*	<0.001	72.0
Sample size					
≥ 200	68	27,454/31,024	1.09 (1.03–1.16)*	<0.001	62.2
< 200	20	1,222/1,515	1.41 (1.14–1.74)*	0.041	38.6
Quality score					
>10	31	19,002/23,225	**1.07 (1.03–1.11)**	0.555	0.0
≤10	57	9,674/9,314	1.18 (1.05–1.32)*	<0.001	69.4
Menopausal status					
Postmenopausal	20	7,934/11,059	1.17 (1.05–1.30)*	0.006	50.1
Premenopausal	17	2,840/3,393	1.18 (1.01–1.38)*	0.014	48.3
Smoking habits					
Current smoker	5	288/334	1.32 (0.96–1.82)	0.659	0.0
Past smoker	7	940/1,000	1.08 (0.91–1.30)	0.943	0.0
Never	11	1,616/1,877	0.98 (0.86–1.13)	0.293	15.8

HB: hospital-based studies; PB: population-based studies

The characters who carried *GSTT1* null genotype had a significantly elevated BC risk (OR = 1.15, 95% CI = 1.06–1.25) in overall analysis. Significant association was also shown in quite a few subgroups, for instance, Caucasians, hospital-based studies, healthy women, cancer-free patients, matching studies, no matching studies, large-sized studies, small-sized studies, low-quality studies, and premenopausal women, as shown in Table 2.

**Table 2:** Meta-analysis of the association of *GSTT1* polymorphism with risk of breast cancer.

Variable	n	Cases/Controls	Test of association	Test of heterogeneity	
			OR (95% CI)	*P*_h_	*I*^2^ (%)
Overall	67	23,092/26,381	1.15 (1.06–1.25)*	<0.001	63.4
Ethnicity					
African	5	1,166/1,095	1.11 (0.92–1.35)	0.202	32.9
Asian	15	3,751/5,425	1.18 (0.95–1.47)*	<0.001	80.2
Caucasian	27	11,139/12,652	1.20 (1.07–1.34)*	0.006	45.7
Indian	7	1,596/1,702	1.34 (0.94–1.92)*	0.002	70.6
Source of controls					
HB	29	4,947/5,489	1.19 (1.03–1.37)*	0.001	52.3
PB	23	15,291/18,337	1.07 (0.96–1.19)*	<0.001	72.5
Type of controls					
Healthy women	22	4,353/5,048	1.32 (1.09–1.60)*	<0.001	68.5
Cancer-free women	36	17,277/19,860	1.04 (0.96–1.14)*	<0.001	55.5
Cancer-free patients	6	701/729	**1.59 (1.22–2.07)**	0.175	34.9
Matching					
Yes	39	16,541/19,760	1.09 (1.00–1.19)*	<0.001	51.3
No	28	6,551/6,621	**1.24 (1.04**–1.46)*	<0.001	71.9
Sample size					
≥ 200	54	22,326/25,482	1.13 (1.04–1.23)*	<0.001	66.9
< 200	13	766/899	**1.34 (1.08–1.65)**	0.145	30.0
Quality score					
>10	23	15,502/19,098	1.05 (0.97–1.14)*	0.002	51.8
≤10	44	7,590/7,283	1.25 (1.08–1.44)*	<0.001	64.6
Menopausal status					
Postmenopausal	15	6,786/9,477	1.08 (0.99–1.17)	0.166	26.2
Premenopausal	12	1,605/1,830	1.31 (1.02–1.67)*	0.053	43.6
Smoking habits					
Current smoker	3	99/135	2.24 (0.67–7.48)*	0.053	66.0
Past smoker	6	832/876	1.45 (0.93–2.26)*	0.082	48.9
Never	9	1,343/1,569	1.00 (0.84–1.20)	0.150	33.5

HB: hospital-based studies, PB: population-based studies

No significantly raised BC risk was observed for *GSTP1* IIe105Val polymorphism in pooling all studies. However, significantly increased BC risk was yielded in some subgroup analyses, such as Asians, Indians, hospital-based studies, no matching, and low-quality studies, as shown in Table 3.

**Table 3:** Meta-analysis of the association of *GSTP1* polymorphism with risk of breast cancer.

Variable	Sample size	Val/Val vs. IIe/IIe		IIe/Val vs. IIe/IIe		Val/Val vs. IIe/IIe + IIe/Val		Val/Val + IIe/Val vs. IIe/IIe		Val vs IIe	
		OR (95% CI)	*P*_h_/*I*^2^	OR (95% CI)	*P*_h_/*I*^2^	OR (95% CI)	*P*_h_/*I*^2^	OR (95% CI)	*P*_h_/*I*^2^	OR (95% CI)	*P*_h_/*I*^2^
Overall	56 (25,331/27,424)	1.06 (0.91–1.25)*	<0.001/73.7	1.04 (0.95–1.15)*	<0.001/77.2	1.05 (0.92–1.19)*	<0.001/62.3	1.05 (0.95–1.15)*	<0.001/80.0	1.05 (0.97–1.14)*	<0.001/83.7
Ethnicity											
African	3 (776/769)	0.86 (0.64–1.15)	0.321/12.1	0.91 (0.72–1.15)	0.627/0.0	0.89 (0.71–1.12)	0.445/0.0	0.89 (0.71–1.12)	0.445/0.0	0.93 (0.81–1.07)	0.328/10.4
Asian	12 (6,473/7,307)	**1.30 (1.08–1.57)**	0.619/0.0	1.15 (0.99–1.35)*	0.012/63.5	**1.25 (1.04–1.51)**	0.574/0.0	**1.12 (1.04–1.20)**	0.120/33.8	**1.12 (1.05–1.19)**	0.107/42.6
Caucasian	26 (13,015/14,246)	0.96 (0.76–1.21)*	<0.001/80.7	0.99 (0.86–1.15)*	<0.001/83.2	0.96 (0.80–1.16)*	<0.001/70.8	0.99 (0.85–1.16)*	<0.001/86.6	1.00 (0.89–1.13)*	<0.001/87.8
Indian	6 (1,357/1,552)	1.58 (1.01–2.47)*	0.039/57.2	1.20 (0.92–1.56)*	0.042/56.6	**1.45 (1.14–1.85)**	0.164/36.4	1.27 (0.94–1.71)*	0.006/69.0	1.24 (0.97–1.57)*	0.003/72.5
Source of controls											
HB	18 (4,817/4,072)	1.11 (0.93–1.31)	0.295/13.9	1.14 (1.01–1.30)*	0.090/34.0	1.06 (0.90–1.25)	0.316/12.0	**1.11 (1.01–1.21)**	0.118/29.3	1.10 (0.99–1.21)	0.082/35.1
PB	23 (17,477/20,320)	1.03 (0.80–1.32)*	<0.001/86.4	0.99 (0.86–1.15)*	<0.001/87.0	1.03 (0.85–1.26)*	<0.001/79.2	1.02 (0.88–1.18)*	<0.001/89.5	1.02 (0.88–1.18)*	<0.001/91.2
Type of controls											
Healthy women	23 (9,350/10,033)	1.11 (0.73–1.68)*	<0.001/85.9	1.06 (0.84–1.32)*	<0.001/88.8	1.06 (0.77–1.47)*	<0.001/77.2	1.04 (0.83–1.30)*	<0.001/89.9	1.06 (0.86–1.30)*	<0.001/92.2
Cancer-free women	28 (14,378/16,163)	1.07 (0.93–1.24)*	0.004/48.4	1.03 (0.95–1.11)*	0.028/38.2	1.07 (0.94–1.21)*	0.015/42.0	1.04 (0.97–1.12)*	0.010/42.6	1.04 (0.98–1.12)*	<0.001/55.5
Matching											
Yes	26 (14,158/16,510)	0.94 (0.75–1.17)*	<0.001/79.1	0.91 (0.79–1.06)*	<0.001/83.3	0.97 (0.82–1.15)*	<0.001/68.9	0.94 (0.82–1.08)*	<0.001/85.5	0.95 (0.85–1.07)*	<0.001/87.4
No	30 (11,173/10,914)	1.22 (0.97–1.53)*	<0.001/63.6	1.18 (1.06–1.31)*	<0.001/56.1	1.14 (0.94–1.38)*	0.001/52.9	1.16 (1.04–1.30)*	<0.001/65.1	1.15 (1.04–1.27)*	<0.001/73.8
Sample size											
≥ 200	47 (24,759/26,931)	1.07 (0.91–1.26)*	<0.001/76.9	1.03 (0.94–1.14)*	<0.001/80.2	1.05 (0.92–1.20)*	<0.001/66.5	1.04 (0.95–1.15)*	<0.001/82.9	1.05 (0.96–1.14)*	<0.001/85.9
< 200	9 (572/493)	1.02 (0.62–1.66)	0.581/0.0	1.22 (0.91–1.63)	0.888/0.0	0.94 (0.60–1.49)	0.603/0.0	1.11 (0.86–1.45)	0.782/0.0	1.09 (0.88–1.34)	0.649/0.0
Quality score											
>10	30 (21,061/23,195)	1.01 (0.82–1.24)*	<0.001/82.5	0.98 (0.87–1.11)*	<0.001/85.6	1.02 (0.87–1.20)*	<0.001/73.0	1.00 (0.88–1.13)*	<0.001/87.7	1.00 (0.90–1.12)*	<0.001/89.6
≤10	26 (4,270/4,229)	1.17 (0.93–1.45)*	0.041/37.3	**1.15 (1.04–1.27)**	0.464/0.0	1.08 (0.89–1.32)*	0.073/32.3	**1.12 (1.03–1.23)**	0.121/25.2	1.12 (1.01–1.24)*	0.008/47.0
Menopausal status											
Postmenopausal	10 (3,448/3,676)	1.06 (0.56–2.00)*	<0.001/82.2	0.87 (0.63–1.20)*	<0.001/83.0	1.16 (0.71–1.89)*	<0.001/71.3	0.90 (0.67–1.22)*	<0.001/85.2	1.00 (0.76–1.31)*	<0.001/87.2
Premenopausal	15 (8,493/11,040)	0.79 (0.52–1.21)*	<0.001/88.0	0.96 (0.75–1.23)*	<0.001/82.8	0.82 (0.58–1.15)*	<0.001/82.8	0.95 (0.75–1.19)*	<0.001/91.2	0.95 (0.77–1.18)*	<0.001/93.1
HWE											
Yes	40 (15,958/15,474)	1.11 (0.95–1.28)*	<0.001/59.4	1.07 (0.98–1.17)*	<0.001/59.8	1.07 (0.95–1.21)*	0.001/46.4	1.09 (0.99–1.20)*	<0.001/70.6	1.08 (0.99–1.17)*	<0.001/75.3
No	8 (8,561/10,766)	0.85 (0.49–1.46)*	<0.001/91.9	0.90 (0.66–1.22)*	<0.001/87.0	0.89 (0.58–1.36)*	<0.001/87.0	0.89 (0.64–1.23)*	<0.001/94.7	0.92 (0.71–1.19)*	<0.001/94.7

HB: hospital-based studies, PB: population-based studies, HWE: Hardy-Weinberg equilibrium

The pooled estimates showed an significant association between the combined effects of both *GSTM1* and *GSTT1* null genotypes on BC risk (*− + vs*. *+ +*: OR = 1.18, 95% CI = 1.03–1.35, *− − vs*. *+ +*: OR = 1.65, 95% CI = 1.31–2.07, (*− +*) *+* (*+ −*) *vs*. *+ +*: OR = 1.17, 95% CI = 1.05–1.30, (*− +*) *+* (*+ −*) + (*− −*) *vs*. *+ +*: OR = 1.27, 95% CI = 1.12–1.43, *− − vs*. (*− +*) *+* (*+ −*) + (*+ +*): OR = 1.41, 95% CI = 1.19–1.68) across overall analysis. In addition, a significantly increased BC risk was observed in all subgroup analyses, as shown in Table 4.

**Table 4:** Meta-analysis of the combined effects of *GSTM1* present/null and *GSTT1* present/null on breast cancer risk.

Variable	N (Case/Control)	+ − vs. + +		− + vs. + +		− − vs. + +	
		OR (95% CI)	*P*_h_/*I*^2^	OR (95% CI)	*P*_h_/*I*^2^	OR (95% CI)	*P*_h_/*I*^2^
Overall	31 (10,497/10,242)	1.04 (0.90–1.21)*	0.069/34.7	1.18 (1.03–1.35)*	0.001/58.6	1.65 (1.31–2.07)*	<0.001/73.8
Ethnicity							
Asian	6 (1,897/2,592)	1.11 (0.90–1.36)	0.628/0.0	1.05 (0.86–1.28)	0.386/1.3	2.06 (1.10–3.84)*	<0.001/84.4
Caucasian	12 (3,749/2,802)	1.06 (0.74–1.53)*	0.039/54.7	1.19 (0.96–1.48)*	0.025/56.2	1.93 (1.31–2.83)*	0.001/67.2
Indian	3 (671/906)	1.05 (0.77–1.45)	0.943/0.0	1.70 (1.09–2.64)*	0.120/52.9	2.12 (0.91–4.97)*	0.091/58.3
Source of controls							
HB	8 (1,192/1,644)	1.37 (0.99–1.89)	0.409/0.0	0.99 (0.78–1.25)	0.856/0.0	**1.58 (1.21–2.06)**	0.758/0.0
PB	10 (5,677/5,473)	0.92 (0.80–1.05)	0.200/29.9	1.26 (1.01–1.56)*	<0.001/77.9	1.40 (1.08–1.82)*	0.003/65.5
Type of controls							
Healthy women	12 (2,366/2,782)	1.12 (0.88–1.43)	0.192/30.9	1.30 (0.99–1.70)*	0.047/50.8	2.05 (1.28–3.26)*	<0.001/70.2
Cancer-free women	12 (5,753/5,408)	0.91 (0.80–1.25)	0.248/22.0	1.13 (0.94–1.35)*	0.004/64.8	1.68 (1.23–2.29)*	<0.001/74.9
Patients	4 (505/640)	**1.74 (1.03–2.95)**	–	1.03 (0.72–1.48)	–	1.32 (0.80–2.18)	–
Sample size							
≥ 200	25 (10,181/9,942)	1.06 (0.91–1.24)*	0.049/39.3	1.18 (1.03–1.36)*	<0.001/62.7	1.56 (1.24–1.96)*	<0.001/74.7
< 200	5 (309/453)	0.64 (0.28–1.45)	0.973/0.0	1.37 (0.63–2.95)	0.933/0.0	**3.82 (1.76–8.29)**	0.449/0.0
Quality score							
>10	13 (7,183/7,124)	0.98 (0.80–1.20)*	0.056/49.0	1.03 (0.93–1.13)	0.291/17.6	1.23 (1.01–1.50)*	0.007/58.6
≤10	18 (3,307/3,222)	1.12 (0.92–1.36)	0.276/17.6	1.36 (1.07–1.72)*	0.002/62.3	2.45 (1.58–3.81)*	<0.001/72.9
Matching							
Yes	17 (6,048/6,558)	0.97 (0.86–1.10)	0.187/26.2	1.04 (0.95–1.14)	0.542/0.0	1.33 (1.10–1.60)*	0.065/40.3
No	12 (4,241/3,414)	1.19 (0.84–1.67)*	0.070/48.5	1.47 (1.07–2.01)*	<0.001/74.8	2.30 (1.38–3.82)*	<0.001/85.0
Menopausal status							
Postmenopausal	7 (1,916/2,268)	0.88 (0.55–1.41)*	0.077/61.0	1.01 (0.84–1.21)	0.997/0.0	**1.49 (1.14–1.94)**	0.498/0.0
Premenopausal	6 (981/1,185)	1.02 (0.44–2.35)*	0.056/65.4	1.01 (0.77–1.32)	0.934/0.0	1.18 (0.91–1.53)	0.143/39.4
Variable	Sample size	(+ −) + (− +) vs. + +		(+ −) + (− +) + (− −) vs. + +		− − vs. (+ +) + (+ −) + (− +)	
		OR (95% CI)	*P*_h_/*I*^2^	OR (95% CI)	*P*_h_/*I*^2^	OR (95% CI)	*P*_h_/*I*^2^
Overall	31 (10,497/10,242)	1.17 (1.05–1.30)*	<0.001/55.9	**1.27 (1.12–1.43)**	<0.001/68.0	1.41 (1.19–1.68)*	<0.001/65.9
Ethnicity							
Asian	6 (1,897/2,592)	1.13 (0.96–1.33)	0.300/18.0	1.40 (0.99–1.98)*	0.012/68.8	1.53 (1.01–2.31)*	<0.001/82.5
Caucasian	12 (3,749/2,802)	1.23 (0.99–1.51)*	0.002/64.9	1.36 (1.10–1.68)*	<0.001/71.1	1.61 (1.22–2.12)*	0.037/46.7
Indian	3 (671/906)	1.48 (1.19–1.84)*	0.204/37.1	1.54 (1.02–2.32)*	0.082/60.0	1.85 (0.85–4.01)*	0.106/55.4
Source of controls							
HB	8 (1,192/1,644)	1.15 (0.94–1.40)	0.673/0.0	**1.22 (1.01–1.47)**	0.569/0.0	**1.42 (1.16–1.75)**	0.376/7.0
PB	10 (5,677/5,473)	1.17 (0.99–1.38)*	0.001/71.2	1.23 (1.04–1.45)*	<0.001/73.7	1.22 (1.01–1.49)*	0.021/54.0
Type of controls							
Healthy women	12 (2,366/2,782)	1.31 (1.04–1.65)*	0.012/57.2	1.43 (1.10–1.85)*	0.001/67.8	1.67 (1.25–2.23)*	0.030/49.7
Cancer-free women	12 (5,753/5,408)	1.10 (0.95–1.29)*	0.011/58.0	1.23 (1.04–1.46)*	<0.001/70.6	1.46 (1.13–1.88)*	<0.001/72.9
Patients	4 (505/640)	1.16 (0.83–1.64)	–	1.20 (0.86–1.66)	–	1.13 (0.78–1.64)	0.213/33.2
Sample size							
≥ 200	25 (10,181/9,942)	1.15 (1.04–1.28)*	0.001/55.7	1.25 (1.11–1.41)*	<0.001/69.3	1.34 (1.13–1.60)*	<0.001/68.0
< 200	5 (309/453)	1.68 (0.57–4.94)*	0.057/65.0	1.60 (0.62–4.12)*	0.051/61.5	2.77 (1.61–4.78)*	0.697/0.0
Quality score							
>10	13 (7,183/7,124)	1.03 (0.96–1.12)	0.265/18.8	1.06 (0.99–1.14)	0.105/35.7	1.16 (0.97–1.37)*	0.005/59.0
≤10	18 (3,307/3,222)	1.36 (1.09–1.69)*	0.001/62.4	1.53 (1.19–1.96)*	<0.001/72.9	1.82 (1.36–2.43)*	0.002/57.7
Matching							
Yes	17 (6,048/6,558)	1.04 (0.95–1.13)	0.399/4.7	1.07 (0.98–1.16)	0.231/20.4	1.25 (1.05–1.48)*	0.018/46.5
No	12 (4,241/3,414)	1.35 (1.09–1.67)*	<0.001/71.2	1.51 (1.20–1.91)*	<0.001/80.0	1.81 (1.24–2.62)*	<0.001/78.4
Menopausal status							
Postmenopausal	7 (1,916/2,268)	1.05 (0.91–1.22)	0.216/30.9	1.14 (0.99–1.31)	0.216/29.2	**1.25 (1.02–1.53)**	0.247/23.9
Premenopausal	6 (981/1,185)	1.01 (0.81–1.23)	0.495/0.0	1.01 (0.83–1.24)	0.523/0.0	1.18 (0.91–1.53)	0.143/39.4

HB: hospital-based studies, PB: population-based studies; + −: *GSTM1* present/*GSTT1* null; − +: *GSTM1* null/*GSTT1* present; − −: *GSTM1* null/*GSTT1* null; + +: *GSTM1* present/*GSTT1* present

Table 5 lists the results of the combined effects of both *GSTM1* present/null and *GSTP1* IIe105Val polymorphisms on BC risk. Overall, a significant association was found between the combined effects of *GSTM1* present/null and *GSTP1* IIe105Val polymorphisms on BC risk (*GSTM1* null/*GSTP1* IIe/IIe + *GSTM1* present/*GSTP1* Val* vs. *GSTM1* present/*GSTP1* IIe/IIe: OR = 1.14, 95% CI = 1.00–1.31, *GSTM1* null/*GSTP1* Val* vs. *GSTM1* present/*GSTP1* IIe/IIe: OR = 1.58, 95% CI = 1.21–2.06, all risk genotypes vs. *GSTM1* present/*GSTP1* IIe/IIe: OR = 1.28, 95% CI = 1.08–1.52, *GSTM1* null/*GSTP1* Val* vs. (*GSTM1* null/*GSTP1* IIe/IIe + *GSTM1* present/*GSTP1* Val* + *GSTM1* present/*GSTP1* IIe/IIe): OR = 1.40, 95% CI = 1.12–1.75). Furthermore, a statistically significant association was also observed in a slice of subgroups, for example, Asians, Caucasians, Indians, no population-based studies, population-based studies, healthy women, cancer-free women, large-sized studies, small-sized studies, high-quality studies, low-quality studies, no matching studies, and controls in HWE studies.

**Table 5:** Meta-analysis of the combined effects of *GSTM1* present/null and *GSTP1* IIe105Val on breast cancer risk.

**Variable**	**Sample size**	***GSTM1* null/ *GSTP1* IIe/IIe vs. *GSTM1* present/ *GSTP1* IIe/IIe**		***GSTM1* present/*GSTP1* Val** ^**1**^ **vs. *GSTM1* present/ *GSTP1* IIe/IIe**		**(*GSTM1* null/ *GSTP1* IIe/IIe + *GSTM1* present/*GSTP1* Val** ^**1**^**) vs. *GSTM1* present/ *GSTP1* IIe/IIe**	
		**OR (95% CI)**	***P***_**h**_**/*I***^**2**^	**OR (95% CI)**	***P***_**h**_**/*I***^**2**^	**OR (95% CI)**	***P***_**h**_**/*I***^**2**^
Overall	15 (6,272/6,739)	1.10 (0.99–1.22)	0.330/11.5	1.08 (0.92–1.27)	0.030/47.1	1.14 (1.00–1.31)*	0.009/52.8
Ethnicity							
Asian	3 (1,934/2,486)	0.99 (0.86–1.16)	0.483/0.0	1.07 (0.89–1.28)	0.620/0.0	1.02 (0.89–1.17)	0.485/0.0
Caucasian	6 (1,148/1,194)	**1.37 (1.03–1.83)**	0.709/0.0	1.41 (0.90–2.22)*	0.069/54.0	**1.60 (1.29–1.98)**	0.286/19.5
Indian	2 (649/896)	1.25 (0.64–2.41)*	0.064/70.9	1.05 (0.63–1.74)*	0.049/74.2	1.10 (0.62–1.94)*	0.017/82.4
Source of controls							
NPB	8 (2,359/2,474)	1.14 (0.91–1.43)	0.306/16.7	1.02 (0.82–1.25)	0.150/38.4	1.20 (0.94–1.54)*	0.009/62.7
PB	7 (3,913/4,265)	1.09 (0.97–1.23)	0.285/19.0	1.12 (0.91–1.39)*	0.025/58.5	1.07 (0.96–1.19)	0.106/42.8
Type of controls							
Healthy women	8 (2,072/2,859)	1.13 (0.95–1.34)	0.446/0.0	1.14 (0.83–1.57)*	0.012/63.2	1.27 (0.98–1.64)*	0.005/65.5
Cancer-free women	4 (2,614/2,698)	1.17 (0.92–1.50)*	0.097/52.5	1.07 (0.92–1.25)	0.411/0.0	1.07 (0.94–1.22)	0.213/33.2
Sample size							
≥ 200	13 (6122/6529)	1.08 (0.97–1.20)	0.469/0.0	1.09 (0.92–1.29)*	0.025/51.0	1.14 (0.99–1.31)*	0.005/58.0
< 200	2 (150/210)	**2.07 (1.09**–**3.94)**	0.793/0.0	0.90 (0.38–2.16)*	0.150/51.7	1.31 (0.78–2.22)	0.433/0.0
Quality score							
>10	9 (5,008/5,197)	1.07 (0.96–1.20)	0.314/14.8	1.09 (0.89–1.33)*	0.033/54.0	1.07 (0.95–1.20)	0.177/30.2
≤10	6 (1,264/1,542)	1.27 (0.97–1.65)	0.394/2.2	1.05 (0.84–1.32)	0.113/46.4	1.35 (0.94–1.93)*	0.009/67.4
Matching							
Yes	8 (3,947/4,762)	1.04 (0.92–1.16)	0.702/0.0	0.96 (0.85–1.09)	0.614/0.0	0.99 (0.90–1.10)	0.567/0.0
No	7 (2,325/1,977)	**1.48 (1.14–1.91)**	0.605/0.0	1.43 (0.98–2.10)	0.071/53.6	1.46 (1.14–1.87)*	0.038/55.1
HWE							
Yes	9 (3767/4276)	**1.16 (1.02–1.33)**	0.573/0.0	1.13 (0.90–1.42)	0.007/64.0	1.23 (0.99–1.51)*	0.002/68.1
No	2 (193/236)	1.61 (0.90–2.88)	0.394/0.0	1.31 (0.75–2.28)	0.835/0.0	1.43 (0.88–2.32)	0.639/0.0
**Variable**	**Sample size**	***GSTM1* null/ GSTP1 Val** ^**1**^ **vs. *GSTM1* present/ *GSTP1* IIe/IIe**		**all risk genotypes vs. *GSTM1* present/ *GSTP1* IIe/IIe**		***GSTM1* null/ GSTP1 Val** ^**1**^ **vs. (*GSTM1* null/ *GSTP1* IIe/IIe + *GSTM1* present/*GSTP1* Val** ^**1**^ **+ *GSTM1* present/ *GSTP1* IIe/IIe)**	
		**OR (95% CI)**	***P***_**h**_**/*I***^**2**^	**OR (95% CI)**	***P***_**h**_**/*I***^**2**^	**OR (95% CI)**	***P***_**h**_**/*I***^**2**^
Overall	15 (6,272/6,739)	1.58 (1.21–2.06)*	<0.001/82.2	1.28 (1.08–1.52)*	<0.001/73.5	1.40 (1.12–1.75)*	<0.001/82.4
Ethnicity							
Asian	3 (1,934/2,486)	**1.23 (1.03–1.46)**	0.604/0.0	1.07 (0.94–1.22)	0.555/0.0	**1.21 (1.04–1.41)**	0.493/0.0
Caucasian	6 (1,148/1,194)	2.11 (1.21–3.66)*	<0.001/79.6	1.77 (1.26–2.48)*	0.034/58.6	1.55 (0.94–2.56)*	<0.001/85.5
Indian	2 (649/896)	2.14 (0.86–5.34)*	0.003/89.1	1.36 (0.63–2.97)*	<0.001/91.8	2.02 (1.14–3.59)*	0.032/78.3
Source of controls							
NPB	8 (2,359/2,474)	**1.41 (1.17–1.70)**	0.376/7.0	1.24 (1.01–1.54)*	0.029/55.1	**1.19 (1.02–1.38)**	0.146/35.3
PB	7 (3,913/4,265)	1.74 (1.10–2.76)*	<0.001/91.5	1.33 (1.01–1.74)*	<0.001/83.9	1.58 (1.08–2.29)*	<0.001/91.3
Type of controls							
Healthy women	8 (2,072/2,859)	1.86 (1.32–2.62)*	0.001/72.7	1.44 (1.10–1.89)*	0.001/72.6	1.25 (0.80–1.96)*	<0.001/90.1
Cancer-free women	4 (2,614/2,698)	1.39 (0.81–2.37)*	<0.001/90.0	1.23 (0.90–1.68)*	0.002/80.4	1.57 (1.15–2.15)*	<0.001/77.9
Sample size							
≥ 200	13 (6,122/6,529)	1.52 (1.15–2.01)*	<0.001/83.9	1.26 (1.05–1.50)*	<0.001/76.2	1.35 (1.07–1.70)*	<0.001/84.1
< 200	2 (1,50/2,10)	**2.36 (1.32–4.22)**	0.656/0.0	**1.64 (1.01–2.66)**	0.470/0.0	**1.99 (1.24–3.20)**	0.986/0.0
Quality score							
>10	9 (5,008/5,197)	1.61 (1.11–2.35)*	<0.001/88.7	**1.23 (1.00–1.52)**	<0.001/79.0	1.51 (1.11–2.06)*	<0.001/88.5
≤10	6 (1,264/1,542)	**1.47 (1.18–1.83)**	0.236/26.5	**1.39 (1.02–1.89)**	0.026/60.7	1.21 (0.94–1.57)	0.094/46.9
Matching							
Yes	8 (3,947/4,762)	1.16 (0.93–1.43)*	0.014/60.2	1.03 (0.93–1.13)	0.252/22.3	1.16 (0.95–1.41)	0.004/66.1
No	7 (2,325/1,977)	2.30 (1.44–3.69)*	<0.001/81.2	1.74 (1.26–2.39)*	<0.001/76.6	1.76 (1.13–2.76)*	<0.001/86.6
HWE							
Yes	9 (3,767/4,276)	1.74 (1.17–2.60)*	<0.001/88.8	1.41 (1.08–1.83)*	<0.001/82.7	1.47 (1.06–2.04)*	<0.001/88.8
No	2 (193/236)	1.56 (0.56–4.33)*	0.061/71.6	1.44 (0.92–2.26)	0.246/25.6	1.23 (0.50–3.00)*	0.041/76.1

PB: population-based studies, HWE: Hardy-Weinberg equilibrium; NPB: no population-based studies; Val ^1^:IIe/Val + Val/Val

Table 6 lists the results of the combined effects of both *GSTT1* present/null and *GSTP1* IIe105Val polymorphisms on BC risk. The results showed an raised BC risk (*GSTT1* null/*GSTP1* Val* vs. *GSTT1* present/*GSTP1* IIe/IIe: OR = 1.44, 95% CI = 1.10–1.88, all risk genotypes vs. *GSTT1* present/*GSTP1* IIe/IIe: OR = 1.23, 95% CI = 1.03–1.48, *GSTT1* null/*GSTP1* Val* vs. (*GSTT1* null/*GSTP1* IIe/IIe + *GSTT1* present/*GSTP1* Val* + *GSTT1* present/*GSTP1* IIe/IIe): OR = 1.26, 95% CI = 1.03–1.54) in all eligible studies. Analyses of subgroups also showed an increased BC risk in Caucasians, Indians, no population-based studies, population-based studies, healthy women, large-sized studies, small-sized studies, high-quality studies, low-quality studies, and no matching studies.

**Table 6:** Meta-analysis of the combined effects of *GSTT1* present/null and *GSTP1* IIe105Val on breast cancer risk.

**Variable**	**Sample size**	***GSTT1* null/ *GSTP1* IIe/IIe vs. *GSTT1* present/ *GSTP1* IIe/IIe**		***GSTT1* present/*GSTP1* Val** ^**1**^ **vs. *GSTT1* present/ *GSTP1* IIe/IIe**		**(*GSTT1* null/*GSTP1* IIe/IIe + *GSTT1* present/*GSTP1* Val** ^**1**^**) vs. *GSTT1* present/ *GSTP1* IIe/IIe**	
		**OR (95% CI)**	***P***_**h**_**/*I***^**2**^	**OR (95% CI)**	***P***_**h**_**/*I***^**2**^	**OR (95% CI)**	***P***_**h**_**/*I***^**2**^
Overall	13 (5,413/5,567)	1.07 (0.94–1.22)	0.121/33.6	1.22 (0.97–1.53)*	<0.001/77.1	1.18 (0.99–1.40)*	<0.001/71.9
Ethnicity							
Asian	2 (1,321/1,610)	1.04 (0.87–1.25)	0.458/0.0	1.14 (0.92–1.42)	0.388/0.0	1.07 (0.91–1.27)	0.865/0.0
Caucasian	5 (912/885)	1.22 (0.65–2.29)*	0.051/57.6	1.45 (0.79–2.66)*	<0.001/84.4	1.50 (0.86–2.60)*	<0.001/82.6
Indian	2 (649/896)	0.80 (0.54–1.18)	0.540/0.0	1.16 (0.62–2.17)*	0.007/86.2	1.09 (0.63–1.88)*	0.014/83.4
Source of controls							
NPB	7 (2,105/2,165)	1.12 (0.75–1.68)*	0.049/55.0	0.98 (0.82–1.18)	0.845/0.0	1.00 (0.87–1.15)	0.768/0.0
PB	6 (3,308/3,402)	1.08 (0.93–1.25)	0.365/8.0	1.48 (1.02–2.15)*	<0.001/88.5	1.40 (1.02–1.93)*	<0.001/86.6
Type of controls							
Healthy women	6 (1,223/1,674)	1.05 (0.68–1.64)*	0.046/55.6	1.31 (0.80–2.14)*	<0.001/84.0	1.35 (0.87–2.08)*	<0.001/82.8
Cancer-free women	4 (2,622/2,711)	1.05 (0.89–1.23)	0.694/0.0	1.15 (0.92–1.45)*	0.062/59.0	1.09 (0.97–1.22)	0.286/20.7
Sample size							
≥ 200	11 (5,263/5,357)	1.05 (0.92–1.20)	0.381/6.6	1.23 (0.96–1.58)*	<0.001/81.2	1.17 (0.97–1.41)*	<0.001/76.2
< 200	2 (150/210)	1.88 (0.51–6.92)*	0.050/74.1	1.11 (0.65–1.90)	0.594/0.0	1.33 (0.81–2.19)	0.685/0.0
Quality score							
>10	8 (4,385/4,334)	1.09 (0.95–1.26)	0.461/0.0	1.39 (0.99–1.95)*	<0.001/86.4	**1.28 (1.00–1.62)**	<0.001/81.9
≤10	5 (1,028/1,233)	1.12 (0.66–1.93)*	0.032/62.2	0.99 (0.82–1.20)	0.744/0.0	0.99 (0.83–1.19)	0.563/0.0
Matching							
Yes	7 (3,342/3,899)	1.09 (0.95–1.26)	0.335/12.4	1.00 (0.84–1.19)*	0.091/45.0	1.01 (0.88–1.18)	0.126/39.8
No	6 (2,071/1,668)	1.08 (0.63–1.86)*	0.053/57.2	1.63 (0.98–2.71)*	<0.001/82.9	1.44 (0.99–2.11)*	<0.001/81.9
HWE							
Yes	7 (2,926/3,104)	1.04 (0.85–1.26)	0.193/30.8	1.35 (0.96–1.90)*	<0.001/87.1	1.30 (0.96–1.78)*	<0.001/85.3
No	2 (193/236)	1.18 (0.62–2.24)	0.574/0.0	1.02 (0.65–1.61)	0.481/0.0	1.05 (0.68–1.61)	0.725/0.0
Only studies with high quality, matching, HWE, and genotyping examination done bindly or quality control							
Yes	3 (1,643/1,661)	1.29 (0.99–1.67)	0.350/4.7	1.06 (0.75–1.51)*	0.023/73.5	1.11 (0.79–1.57)*	0.021/74.2
**Variable**	**Sample size**	***GSTT1* null/ GSTP1 Val** ^**1**^ **vs. *GSTT1* present/ *GSTP1* IIe/IIe**		**all risk genotypes vs. *GSTT1* present/ *GSTP1* IIe/IIe**		***GSTT1* null/ GSTP1 Val** ^**1**^ **vs. (*GSTT1* null/ *GSTP1* IIe/IIe + *GSTT1* present/*GSTP1* Val** ^**1**^ **+ *GSTT1* present/ *GSTP1* IIe/IIe)**	
		**OR (95% CI)**	***P***_**h**_**/*I***^**2**^	**OR (95% CI)**	***P***_**h**_**/*I***^**2**^	**OR (95% CI)**	***P***_**h**_**/*I***^**2**^
Overall	13 (5,413/5,567)	1.44 (1.10–1.88)*	<0.001/68.4	1.23 (1.03–1.48)*	<0.001/76.3	1.26 (1.03–1.54)*	0.013/52.7
Ethnicity							
Asian	2 (1,321/1,610)	1.34 (0.85–2.11)*	0.099/63.3	1.11 (0.95–1.30)*	0.481/0.0	1.29 (0.83–1.98)*	0.073/68.8
Caucasian	5 (912/885)	2.09 (1.15–3.80)*	0.037/60.8	1.60 (0.92–2.79)*	<0.001/83.9	**1.50 (1.11–2.02)**	0.358/8.4
Indian	2 (649/896)	1.62 (0.92–2.87)*	0.114/60.0	1.16 (0.66–2.05)*	0.008/85.6	**1.55 (1.12–2.15)**	0.478/0.0
Source of controls							
NPB	7 (2,105/2,165)	**1.51 (1.18–1.93)**	0.582/0.0	1.06 (0.92–1.21)	0.540/0.0	**1.48 (1.18–1.85)**	0.761/0.0
PB	6 (3,308/3,402)	1.36 (0.89–2.09)*	<0.001/82.6	1.42 (1.02–1.99)*	<0.001/88.9	1.10 (0.83–1.47)*	0.011/66.4
Type of controls							
Healthy women	6 (1,223/1,674)	1.85 (1.21–2.82)*	0.041/56.8	1.46 (0.95–2.25)*	<0.001/84.0	**1.52 (1.21–1.92)**	0.444/0.0
Cancer-free women	4 (2,622/2,711)	1.21 (0.80–1.82)*	0.005/76.3	1.12 (0.93–1.36)*	0.071/57.3	1.11 (0.80–1.54)*	0.023/68.4
Sample size							
≥ 200	11 (5,263/5,357)	**1.36 (1.03–1.79)**	<0.001/69.9	1.21 (0.99–1.47)*	<0.001/79.4	1.21 (0.98–1.48)*	0.019/53.1
< 200	2 (150/210)	**2.30 (1.16–4.54)**	0.265/19.4	1.51 (0.93–2.44)	0.558/0.0	**1.87 (1.05–3.31)**	0.272/17.2
Quality score							
> 10	8 (4,385/4,334)	1.44 (0.99–2.08)*	<0.001/78.8	1.32 (1.02–1.69)*	<0.001/84.8	1.21 (0.93–1.57)*	<0.001/65.6
≤10	5 (1,028/1,233)	**1.39 (1.04–1.87)**	0.434/0.0	1.05 (0.88–1.25)	0.406/0.0	**1.38 (1.06–1.81)**	0.632/0.0
Matching							
Yes	7 (3,342/3,899)	1.09 (0.85–1.41)*	0.040/54.5	1.03 (0.89–1.20)*	0.075/47.6	1.06 (0.86–1.32)*	0.075/47.6
No	6 (2,071/1,668)	2.12 (1.37–3.28)*	0.066/51.6	1.54 (1.05–2.28)*	<0.001/83.7	**1.60 (1.25–2.04)**	0.461/0.0
HWE							
Yes	7 (2,926/3,104)	1.38 (0.91–2.10)*	<0.001/79.4	1.33 (0.97–1.84)*	<0.001/87.3	1.17 (0.87–1.56)*	0.013/63.0
No	2 (193/236)	1.47 (0.75–2.87)	0.829/0.0	1.10 (0.73–1.68)	0.659/0.0	1.39 (0.78–2.49)	0.964/0.0
Only studies with high quality, matching, HWE, and genotyping examination done bindly or quality control							
Yes	3 (1643/1661)	0.83 (0.66–1.04)*	0.125/52.0	1.08 (0.76–1.53)*	0.015/76.1	0.82 (0.66–1.02)	0.601/0.0

* A random-effect model was used when *P* < 0.10 and/or I^2^ > 50%; otherwise, a fixed-effects model was used, PB: population-based studies, HWE: Hardy-Weinberg equilibrium; NPB: no population-based studies; Val ^1^:IIe/Val + Val/Val

Finally, we also performed a pooled analysis to investigate the combined effects of *GSTM1* present/null, *GSTT1* present/null, and *GSTP1* IIe105Val polymorphisms on BC risk. The results indicated that a significantly increased BC risk was found in overall populations (Table 7); respective OR was 1.44 (95% CI = 1.00–2.06) for *GSTM1* null/*GSTT1* null/*GSTP1* IIe/IIe vs. *GSTM1* present/*GSTT1* present/*GSTP1* IIe/IIe, OR was 1.54 (95% CI = 1.08–2.18) for *GSTM1* null/*GSTT1* present/*GSTP1* Val* vs. *GSTM1* present/*GSTT1* present/*GSTP1* IIe/IIe, OR was 1.41 (95% CI = 1.08–1.83) for all two high-risk genotypes vs. *GSTM1* present/*GSTT1* present/*GSTP1* IIe/IIe, OR was 1.79 (95% CI = 1.19–2.67) for *GSTM1* null/*GSTT1* null/*GSTP1* Val* vs. *GSTM1* present/*GSTT1* present/*GSTP1* IIe/IIe, and OR was 1.51 (95% CI = 1.10–2.06) for *GSTM1* null/*GSTT1* null/*GSTP1* Val* vs. (all one high-risk genotypes + all two high-risk genotypes + *GSTM1* present/*GSTT1* present/*GSTP1* IIe/IIe). The results of subgroups indicated that significant association was also observed in Caucasians, Indians, no population-based studies, population-based studies, healthy women, large-sized studies, small-sized studies, high-quality studies, low-quality studies, no matching studies, and controls in HWE studies, as shown in Table 7.

**Table 7:** Meta-analysis of the combined effects of *GSTM1* present/null, *GSTT1* present/null and *GSTP1* present/null on breast cancer risk.

**Variable**	**Sample size**	***M1* null/*T1* present/*P1* IIe/IIe vs. *M1* present/*T1* present/*P1* IIe/IIe**		***M1* present/*T1* null/*P1* IIe/IIe vs. *M1* present/*T1* present/*P1* IIe/IIe**		***M1* present/*T1* present/*P1* Val** ^**1**^ **vs. *M1* present/*T1* present/*P1* IIe/IIe**		**all one high-risk genotype vs. vs. *M1* present/*T1* present/*P1* IIe/IIe**		**M1 null/T1 null/P1 IIe/IIe vs. *M1* present/*T1* present/*P1* IIe/IIe**	
		**OR (95% CI)**	***P***_**h**_**/*I***^**2**^	**OR (95% CI)**	***P***_**h**_**/*I***^**2**^	**OR (95% CI)**	***P***_**h**_**/*I***^**2**^	**OR (95% CI)**	***P***_**h**_**/*I***^**2**^	**OR (95% CI)**	***P***_**h**_**/*I***^**2**^
Overall	13 (5,395/5,544)	1.05 (0.91–1.21)	0.682/0.0	0.96 (0.80–1.15)	0.695/0.0	1.04 (0.85–1.27)*	0.065/41.5	1.03 (0.92–1.14)	0.356/8.9	1.44 (1.00–2.06)*	0.016/52.9
Ethnicity											
Asian	2 (1,321/1,610)	0.87 (0.67–1.13)	0.342/0.0	0.96 (0.73–1.26)	0.768/0.0	0.91 (0.65–1.26)	0.387/0.0	0.91 (0.72–1.14)	0.421/0.0	0.96 (0.74–1.25)	0.834/0.0
Caucasian	5 (912/885)	1.31 (0.95–1.82)	0.992/0.0	1.06 (0.64–1.73)	0.513/0.0	**1.46 (1.07–1.99)**	0.108/47.3	**1.36 (1.03–1.77)**	0.678/0.0	1.92 (0.71–5.17)*	0.029/62.9
Indian	2 (649/896)	1.26 (0.86–1.86)	0.371/0.0	0.76 (0.47–1.21)	0.526/0.0	0.90 (0.68–1.20)	0.187/42.5	0.95 (0.73–1.23)	0.239/28.0	0.56 (0.03–11.41)*	0.038/76.9
Source of controls											
NPB	7 (2,099/2,161)	1.11 (0.84–1.45)	0.564/0.0	0.95 (0.68–1.33)	0.763/0.0	0.96 (0.75–1.23)	0.229/27.4	1.03 (0.88–1.20)	0.524/0.0	1.52 (0.69–3.37)*	0.016/64.2
PB	6 (3,296/3,383)	1.03 (0.87–1.22)	0.511/0.0	0.96 (0.77–1.20)	0.316/15.4	1.12 (0.85–1.47)*	0.040/57.2	1.02 (0.89–1.17)	0.155/37.7	1.42 (0.96–2.12)*	0.094/46.9
Type of controls											
Healthy women	6 (1,223/1,674)	1.11 (0.85–1.46)	0.560/0.0	0.96 (0.68–1.35)	0.629/0.0	1.12 (0.69–1.84)*	0.008/68.3	1.07 (0.87–1.31)	0.104/45.2	1.27 (0.54–2.99)*	0.018/63.2
Cancer-free women	4 (2,610/2,692)	1.03 (0.86–1.24)	0.477/0.0	0.91 (0.73–1.14)	0.757/0.0	1.02 (0.84–1.23)	0.962/0.0	1.00 (0.86–1.17)	0.911/0.0	1.45 (0.91–2.30)*	0.059/59.7
Sample size											
≥ 200	11 (5,245/5,334)	1.04 (0.90–1.20)	0.559/0.0	0.95 (0.79–1.14)	0.771/0.0	1.06 (0.85–1.30)*	0.041/48.7	1.02 (0.92–1.14)	0.207/23.8	1.15 (0.94–1.40)	0.102/38.4
< 200	2 (150/210)	1.45 (0.63–3.37)	0.953/0.0	1.27 (0.54–2.96)	0.156/50.2	0.88 (0.41–-1.88)	0.287/11.6	1.11 (0.58–2.12)	0.948/0.0	**5.58 (1.96–15.89)**	0.171/46.6
Quality score											
> 10	8 (4,367/4,311)	1.00 (0.85–1.18)	0.413/1.5	0.95 (0.78–1.17)	0.441/0.0	1.07 (0.82–1.39)*	0.046/53.2	1.02 (0.91–1.15)	0.219/26.3	1.20 (0.97–1.48)	0.137/38.2
≤10	5 (1,028/1,233)	1.24 (0.92–1.69)	0.946/0.0	0.98 (0.67–1.45)	0.643/0.0	0.99 (0.77–1.29)	0.203/32.8	1.05 (0.83–1.31)	0.458/0.0	1.74 (0.56–5.43)*	0.010/70.0
Matching											
Yes	7 (3,330/3,880)	0.97 (0.82–1.14)	0.721/0.0	0.98 (0.80–1.20)	0.733/0.0	0.91 (0.77–1.06)	0.689/0.0	0.94 (0.82–1.07)	0.584/0.0	1.32 (0.89–1.94)*	0.067/49.0
No	6 (2,065/1,664)	**1.39 (1.03–1.86)**	0.995/0.0	0.90 (0.56–1.46)	0.309/16.4	1.39 (0.91–2.12)*	0.085/51.1	**1.18 (1.00–1.39)**	0.562/0.0	1.81 (0.76–4.32)	0.031/62.4
HWE											
Yes	7 (2,914/3,085)	1.12 (0.94–1.34)	0.642/0.0	0.94 (0.72–1.21)	0.445/0.0	1.13 (0.86–1.49)*	0.012/63.1	1.09 (0.89–1.34)*	0.083/46.3	**1.40 (1.03–1.90)**	0.156/35.7
No	2 (193/236)	1.20 (0.60–2.41)	0.795/0.0	0.78 (0.32–1.91)	0.747/0.0	1.14 (0.60–2.18)	0.727/0.0	1.07 (0.61–1.88)	0.989/0.0	2.38 (0.93–6.05)	0.825/0.0
Only studies with high quality, matching, HWE, and genotyping examination done bindly or quality control											
Yes	3 (1,631/1,642)	1.16 (0.85–1.58)	0.222/33.6	1.05 (0.74–1.48)	0.201/37.6	0.95 (0.76–1.17)	0.355/3.5	0.98 (0.81–1.19)	0.218/34.3	**1.84 (1.22–2.77)**	0.644/0.0
**Variable**	**Sample size**	**M1 null/T1 present/P1 Val** ^**1**^ **vs. *M1* present/*T1* present/*P1* IIe/IIe**		**M1 present/T1 null/P1 Val** ^**1**^ **vs. *M1* present/*T1* present/*P1* IIe/IIe**		**all two high-risk genotype *vs*. *M1* present/*T1* present/*P1* IIe/IIe**		**M1 null/T1 null/P1 Val** ^**1**^ **vs. *M1* present/*T1* present/*P1* IIe/IIe**		**M1 null/T1 null/P1 Val** ^**1**^ **vs. (all one high-risk genotypes + all two high-risk genotypes + *M1* present/*T1* present/*P1* IIe/IIe)**	
		**OR (95% CI)**	***P***_**h**_**/*I***^**2**^	**OR (95% CI)**	***P***_**h**_**/*I***^**2**^	**OR (95% CI)**	***P***_**h**_**/*I***^**2**^	**OR (95% CI)**	***P***_**h**_**/*I***^**2**^	**OR (95% CI)**	***P***_**h**_**/*I***^**2**^
Overall	13 (5,395/5,544)	1.54 (1.08–2.18)*	<0.001/81.1	1.06 (0.88–1.28)	0.167/29.3	1.41 (1.08–1.83)*	<0.001/77.7	1.79 (1.19–2.67)*	<0.001/72.1	1.51 (1.10–2.06)*	0.001/63.3
Ethnicity											
Asian	2 (1,321/1,610)	1.18 (0.87–1.61)	0.219/33.9	1.14 (0.82–1.59)	0.445/0.0	1.06 (0.84–1.34)	0.582/0.0	1.37 (0.61–3.06)	0.042/75.9	1.52 (0.57–-4.10)*	0.002/89.4
Caucasian	5 (912/885)	1.98 (0.92–4.27)*	<0.001/81.3	1.62 (0.95–2.77)	0.584/0.0	2.07 (1.06–4.04)*	0.001/78.4	2.64 (1.23–5.66)*	0.035/61.4	**1.63 (1.13–2.36)**	0.133/43.3
Indian	2 (649/896)	1.87 (0.78–4.48)*	0.010/85.1	1.32 (0.85–2.05)	0.242/26.8	1.56 (0.66–3.72)*	0.003/88.4	**3.10 (1.77–5.42)**	0.173/46.1	**2.58 (1.51–4.40)**	0.543/0.0
Source of controls											
NPB	7 (2,099/2,161)	1.17 (0.90–1.51)	0.624/0.0	1.21 (0.84–1.74)	0.538/0.0	1.12 (0.92–1.35)	0.338/11.9	**1.83 (1.30–2.58)**	0.432/0.0	**1.75 (1.29–2.36)**	0.206/29.2
PB	6 (3,296/3,383)	1.94 (1.08–3.48)*	<0.001/90.7	1.05 (0.73–1.51)*	0.051/57.5	1.71 (1.06–2.75)*	<0.001/89.1	1.72 (0.89–3.33)8	<0.001/84.8	1.28 (0.84–1.97)*	0.004/71.0
Type of controls											
Healthy women	6 (1,223/1,674)	1.73 (0.93–3.20)*	<0.001/79.5	1.15 (0.79–1.66)	0.613/0.0	1.69 (0.98–2.92)*	<0.001/79.9	**2.30 (1.62–3.26)**	0.125/42.0	**1.99 (1.46–2.70)**	0.230/27.3
Cancer-free women	4 (2,610/2,692)	1.37 (0.82–2.28)*	<0.001/83.9	1.17 (0.69–1.96)*	0.019/69.7	1.31 (0.87–1.96)*	0.001/80.8	1.38 (0.74–2.56)*	0.004/77.9	1.22 (0.77–1.92)*	0.027/67.4
Sample size											
≥ 200	11 (5,245/5,334)	1.49 (1.02–2.19)*	<0.001/84.2	1.05 (0.87–1.27)	0.121/37.2	1.34 (1.02–1.76)*	<0.001/79.6	1.60 (1.06–2.41)*	<0.001/72.5	1.37 (1.00–1.88)*	0.003/63.0
< 200	2 (150/210)	1.97 (0.93–4.15)	0.719/0.0	1.35 (0.49–3.74)	0.281/13.8	**2.27 (1.16–4.45)**	0.272/17.0	**4.37 (1.75–10.92)**	0.752/0.0	**3.14 (1.47–6.72)**	0.968/0.0
Quality score											
> 10	8 (4,367/4,311)	1.72 (1.01–2.93)*	<0.001/89.3	1.02 (0.74–1.40)*	0.089/47.6	1.45 (1.01–2.09)*	<0.001/85.5	1.72 (1.00–2.94)*	<0.001/80.3	1.43 (0.95–2.14)*	0.001/72.3
≤10	5 (1,028/1,233)	1.24 (0.94–1.63)	0.704/0.0	1.33 (0.88–2.02)	0.524/0.0	1.24 (0.96–1.59)	0.258/24.6	**1.79 (1.19–2.70)**	0.248/26.0	**1.56 (1.08–2.24)**	0.197/33.6
Matching											
Yes	7 (3,330/3,880)	1.10 (0.82–1.47)*	0.021/59.8	0.95 (0.77–1.17)	0.220/27.3	1.03 (0.89–1.19)	0.107/42.6	1.18 (0.80–1.75)*	0.025/58.3	1.19 (0.83–1.72)*	0.012/63.3
No	6 (2,065/1,664)	2.50 (1.29–4.84)*	<0.001/81.0	**1.57 (1.04–2.37)**	0.733/0.0	2.00 (1.18–3.38)*	<0.001/83.1	2.99 (1.66–5.41)*	0.049/55.1	**2.08 (1.35–3.21)**	0.176/34.7
HWE											
Yes	7 (2,914/3,085)	1.78 (1.06–3.01)*	<0.001/89.0	1.06 (0.74–1.51)*	0.079/49.4	1.61 (1.03–2.50)*	<0.001/87.0	**1.87 (1.00–3.50)**	<0.001/80.3	1.42 (0.93–2.18)*	0.009/64.7
No	2 (193/236)	1.13 (0.61–2.10)	0.395/0.0	1.48 (0.55–4.03)	0.226/31.8	1.30 (0.73–2.31)	0.694/0.0	1.79 (0.75–4.29)*	0.131/56.3	1.64 (0.46–5.81)*	0.101/62.8
Only studies with high quality, matching, HWE, and genotyping examination done bindly or quality control											
Yes	3 (1,631/1,642)	1.24 (0.65–2.35)*	0.002/83.5	0.76 (0.56–1.04)	0.271/23.4	1.20 (0.71–2.04)*	0.007/79.6	1.08 (0.48–2.45)*	0.017/75.5	0.92 (0.67–1.26)	0.161/45.3

* A random-effect model was used when *P* < 0.10 and/or I^2^ > 50%; otherwise, a fixed-effects model was used, PB: population-based studies, HWE: Hardy-Weinberg equilibrium; NPB: no population-based studies; Val ^1^:IIe/Val + Val/Val

### Heterogeneity and sensitivity analyses

Heterogeneity was observed in the current meta-analysis, as shown in Tables 1–7. Then, we evaluated heterogeneity source by applying a meta-regression analysis method. The results suggested that source of controls (*P* = 0.027 for *+ − vs*. *+ +*), type of controls (*P* = 0.005 for *− −* vs. *+ +*), and quality score of articles (*− +* vs. *+ +*: *P* = 0.045 for *− −* vs. *+ +*) were source of heterogeneity between the combined effects of *GSTM1* present/null and GSTT1 present/null polymorphisms with BC risk. For the combined effects of *GSTM1* present/null and *GSTP1* IIe105Val polymorphisms, matching (*GSTM1* present/*GSTP1* Val* vs. *GSTM1* present/*GSTP1* IIe/IIe: *P* = 0.041; all one risk genotypes vs. *GSTM1* present/*GSTP1* IIe/IIe: *P* = 0.018) was source of heterogeneity.

Sensitivity analysis was estimated by applying two methods in this meta-analysis. First, results did not change when removing a single study each time to appraise the robustness in the current meta-analysis. However, when we restrained only high-quality studies, HWE, matching, and genotyping examination performed blindly or with quality control, significantly increased BC risk was found in the overall analysis for the combined effects of *GSTM1*, *GSTT1*, and *GSTP1* IIe105Val polymorphisms (*GSTM1* null/*GSTT1* null/*GSTP1* null vs. *GSTM1* present/*GSTT1* present/*GSTP1* IIe/IIe: OR = 1.84, 95% CI = 1.22–2.77), *GSTM1* null genotype (OR = 1.06, 95% CI = 1.02–1.11), all races (*− −* vs. *+ +*: OR = 1.27, 95% CI = 1.02–1.59), Caucasians (*− −* vs. *+ +*: OR = 1.58, 95% CI = 1.10–2.29, *− −* vs. (*+ +*) *+* (*− +*) *+* (*+ −*): OR = 1.58, 95% CI = 1.11–2.24), and postmenopausal women (*− − vs*. *+ +*: OR = 1.50, 95% CI = 1.13–2.00, *− − vs*. (*+ +*) *+* (*− +*) *+* (*+ −*): OR = 1.29, 95% CI = 1.03–1.61), and the combined effects of *GSTM1* and *GSTT1* polymorphisms, and, as shown in Tables 7–9, respectively; no significant association was observed for the combined of *GSTT1* and *GSTP1* IIe105Val polymorphisms, *GSTT1*,*GSTP1*, and the combined effects of *GSTM1* and *GSTP1* IIe105Val polymorphisms, and, as shown in Tables 6, 10, 11 and 12, respectively.

**Table 8:** Pooled estimates of association of *GSTM1* polymorphism and breast cancer risk, only studies with high quality, matching, and genotyping examination done bindly or quality control.

Variable	n	Cases/Controls	Test of association	Test of heterogeneity	
			OR (95% CI)	*P*_h_	*I*^2^ (%)
Overall	21	14,524/17,745	**1.06 (1.02–1.11)**	0.578	0.0
Ethnicity					
African	2	733/701	0.93 (0.74–1.18)	0.658	0.0
Asian	5	2,483/3,539	1.04 (0.94–1.15)	0.603	0.0
Caucasian	7	7,065/9,184	1.05 (0.99–1.12)	0.695	0.0
Menopausal status					
Postmenopausal	12	6,524/9,463	0.95 (0.89–1.01)	0.272	17.5
Premenopausal	9	1,419/1,910	1.02 (0.88–1.17)	0.535	0.0
Smoking habits					
Current smoker	2	81/80	0.61 (0.33–1.15)	0.849	0.0
Past smoker	2	240/229	0.95 (0.66–1.36)	0.397	0.0
Never	2	231/249	1.27 (0.88–1.82)	0.936	0.0

**Table 9:** Pooled estimates of association of the combined effects of *GSTM1* present/null and *GSTT1* present/null and breast cancer risk, only studies with high quality, matching, and genotyping examination done bindly or quality control.

Variable	N (Case/Control)	+ − vs. + +		− + vs. + +		− − vs. + +	
		OR (95% CI)	*P*_h_/*I*^2^	OR (95% CI)	*P*_h_/*I*^2^	OR (95% CI)	*P*_h_/*I*^2^
Overall	9 (5,175/5,055)	0.98 (0.78–1.23)*	0.034/56.1	1.03 (0.94–1.14)	0.269/21.1	1.27 (1.02–1.59)*	0.038/53.0
Ethnicity							
Asian	3 (1,512/1,948)	1.07 (0.84–1.37)	–	1.05 (0.83–1.32)	–	1.31 (0.66–2.61)*	0.023/80.7
Caucasian	1 (1,235/659)	0.73 (0.51–1.25)	–	1.06 (0.86–1.31)	–	**1.58 (1.10–2.29)**	–
Menopausal status							
Postmenopausal	5 (1,487/1,749)	0.88 (0.55–1.41)*	0.077/61.0	1.01 (0.84–1.21)	0.997/0.0	**1.50 (1.13–2.00)**	0.343/10.0
Premenopausal	4 (621/742)	1.02 (0.44–2.35)*	0.056/65.4	1.01 (0.77–1.32)	0.934/0.0	1.07 (0.61–1.86)*	0.110/50.2
Variable	Sample size	(+ −) + (− +) vs. + +		(+ −) + (− +) + (− −) vs. + +		− − vs. (+ +) + (+ −) + (− +)	
		OR (95% CI)	*P*_h_/*I*^2^	OR (95% CI)	*P*_h_/*I*^2^	OR (95% CI)	*P*_h_/*I*^2^
Overall	9 (5,175/5,055)	1.02 (0.94–1.12)	0.122/38.6	1.08 (0.96–1.23)*	0.069/46.6	1.16 (0.98–1.38)*	0.067/45.2
Ethnicity							
Asian	3 (1,512/1,948)	1.12 (0.93–1.36)	0.184/43.3	1.23 (0.79–1.90)*	0.073/68.8	0.98 (0.84–1.14)	0.165/44.5
Caucasian	1 (1,235/659)	1.00 (0.82–1.22)	–	1.07 (0.88–1.30)	–	**1.58 (1.11–2.24)**	–
Menopausal status							
Postmenopausal	5 (1,487/1,749)	0.98 (0.83–1.16)	0.513/0.0	1.04 (0.89–1.22)	0.464/0.0	**1.29 (1.03–1.61)**	0.115/46.1
Premenopausal	4 (621/742)	1.06 (0.84–1.34)	0.485/0.0	1.05 (0.84–1.32)	0.423/0.0	0.99 (0.60–1.65)*	0.088/54.2

* A random-effect model was used when *P* < 0.10 and/or I^2^ > 50%; otherwise, a fixed-effects model was used, + −: *GSTM1* present/*GSTT1* null; − +: *GSTM1* null/*GSTT1* present; − −: *GSTM1* null/*GSTT1* null; + +: *GSTM1* present/*GSTT1* present

**Table 10:** Pooled estimates of association of *GSTT1* polymorphism and breast cancer risk, only studies with high quality, matching, and genotyping examination done bindly or quality control.

Variable	n	Cases/Controls	Test of association	Test of heterogeneity	
			OR (95% CI)	*P*_h_	*I*^2^ (%)
Overall	17	12,980/15,456	1.05 (0.96–1.14)*	0.007	51.5
Ethnicity					
African	2	742/707	1.01 (0.79–1.30)	0.277	15.2
Asian	4	1,869/2,666	1.01 (0.83–1.23)*	0.098	52.3
Caucasian	5	6,605/8,242	1.12 (0.97–1.30)*	0.060	55.8
Menopausal status					
Postmenopausal	9	5,912/8,518	0.93 (0.85–1.02)*	0.064	45.8
Premenopausal	6	946/1,034	0.98 (0.79–1.21)	0.522	0.0
Smoking habits					
Current smoker	1	51/58	0.84 (0.36–1.99)	–	–
Past smoker	1	135/130	0.57 (0.32–1.01)	–	–
Never	1	131/146	0.69 (0.41–1.18)	–	–

* A random-effect model was used when *P* < 0.10 and/or I^2^ > 50%; otherwise, a fixed-effects model was used,

**Table 11:** Pooled estimates of association of *GSTP1* polymorphism and breast cancer risk, only studies with controls in Hardy-Weinberg equilibrium, high quality, matching, and genotyping examination done bindly or quality control.

Variable	Sample size	Val/Val vs. IIe/IIe		IIe/Val vs. IIe/IIe		Val/Val vs. IIe/IIe + IIe/Val		Val/Val + IIe/Val vs. IIe/IIe		Val vs IIe	
		OR (95% CI)	*P*_h_/*I*^2^	OR (95% CI)	*P*_h_/*I*^2^	OR (95% CI)	*P*_h_/*I*^2^	OR (95% CI)	*P*_h_/*I*^2^	OR (95% CI)	*P*_h_/*I*^2^
Overall	12 (7,282/6,774)	0.95 (0.84–1.06)	0.521/0.0	0.96 (0.90–1.03)	0.209/23.9	0.97 (0.87–1.08)	0.650/0.0	0.96 (0.90–1.03)	0.182/26.7	0.97 (0.92–1.02)	0.245/20.2
Ethnicity											
African	2 (720/692)	0.81 (0.59–1.09)	0.776/0.0	0.88 (0.68–1.12)	0.916/0.0	0.88 (0.68–1.14)	0.656/0.0	0.86 (0.68–1.08)	0.987/0.0	0.90 (0.78–1.05)	0.765/0.0
Asian	2 (784/1,047)	0.96 (0.57–1.61)	0.702/0.0	1.09 (0.89–1.33)	0.136/55.0	0.93 (0.55–1.56)	0.839/0.0	1.08 (0.89–1.30)	0.134/55.5	1.05 (0.89–1.23)	0.175/45.7
Caucasian	5 (3,725/2,959)	0.99 (0.85–1.17)	0.744/0.0	0.95 (0.85–1.05)	0.197/33.7	1.02 (0.88–1.19)	0.925/0.0	0.96 (0.87–1.05)	0.190/34.6	0.98 (0.91–1.05)	0.333/12.7

**Table 12:** Pooled estimates of association of the combined effects of *GSTM1* present/null and *GSTP1* IIe105Val and breast cancer risk, only studies with high quality, matching, HWE, and genotyping examination done bindly or quality control.

**Variable**	**Sample size**	***GSTM1* null/ *GSTP1* IIe/IIe vs. *GSTM1* present/ *GSTP1* IIe/IIe**		***GSTM1* present/GSTP1 Val** ^**1**^ **vs. *GSTM1* present/ *GSTP1* IIe/IIe**		**(*GSTM1* null/ *GSTP1* IIe/IIe + *GSTM1* present/*GSTP1* Val** ^**1**^**) vs. *GSTM1* present/*GSTP1* IIe/IIe**	
		**OR (95% CI)**	***P***_**h**_**/*I***^**2**^	**OR (95% CI)**	***P***_**h**_**/*I***^**2**^	**OR (95% CI)**	***P***_**h**_**/*I***^**2**^
Overall	4 (2,488/2,524)	1.12 (0.96–1.32)	0.677/0.0	0.95 (0.81–1.12)	0.417/0.0	1.03 (0.90–1.19)	0.370/4.6
Ethnicity							
Asian	1 (613/876)	1.13 (0.87–1.47)	–	1.12 (0.83–1.52)	–	1.13 (0.89–1.43)	–
**Variable**	**Sample size**	*GSTM1* null/ GSTP1 Val* **vs. *GSTM1* present/ *GSTP1* IIe/IIe**		**all risk genotypes vs. *GSTM1* present/ *GSTP1* IIe/IIe**		*GSTM1* null/ GSTP1 Val*vs. (*GSTM1* null/ *GSTP1* IIe/IIe + *GSTM1* present/*GSTP1* Val*+ *GSTM1* present/ *GSTP1* IIe/IIe)	
		**OR (95% CI)**	***P***_**h**_**/*I***^**2**^	**OR (95% CI)**	***P***_**h**_**/*I***^**2**^	**OR (95% CI)**	***P***_**h**_**/*I***^**2**^
Overall	4 (2,488/2,524)	1.17 (0.77–1.77)*	0.001/80.9	1.07 (0.84–1.36)*	0.041/63.7	1.14 (0.83–1.57)*	0.002/79.1
Ethnicity							
Asian	1 (613/876)	1.33 (0.99–1.80)	–	1.18 (0.95–1.48)	–	1.24 (0.95–1.61)	–

Val ^1^:IIe/Val + Val/Val,

* A random-effect model was used when *P* < 0.10 and/or I^2^ > 50%; otherwise, a fixed-effects model was used,

### Evaluation of publication bias

There was no evidence of publication bias for *GSTM1* (*P* = 0.223, S1 Fig), *GSTT1* (*P* = 0.079, S2 Fig), and *GSTP1* IIe105Val (Val/Val vs. IIe/IIe: *P* = 0.884, IIe/Val vs. IIe/IIe: *P* = 0.153; Val/Val vs. IIe/IIe +IIe/Val: *P* = 0.596; Val vs. IIe: *P* = 0.505; Val/Val + IIe/Val vs. IIe/IIe: *P* = 0.478, S3–S7 Figs) on BC risk. However, there was significant evidence of publication bias for the combined effects of both *GSTM1* and *GSTT1* polymorphisms (*− −* vs. *+ +*: *P* < 0.001, (*+ −*) + (*− +*) vs. (*+ +*): *P* = 0.005, (*− +*) *+* (*+ −*) + (*− −*) vs. *+ +*: *P =* 0.002, *− −* vs. (*− +*) *+* (*+ −*) + (*+ +*): *P =* 0.001), the combined effects of *GSTM1* and *GSTP1* IIe105Val polymorphisms (*GSTM1* null/*GSTP1* Val* vs. *GSTM1* present/*GSTP1* IIe/IIe: *P =* 0.038, all risk genotypes vs. *GSTM1* present/*GSTP1* IIe/IIe: *P =* 0.028), the combined effects of *GSTT1* and *GSTP1* IIe105Val polymorphisms (*GSTT1* null/*GSTP1* val* vs. G*STT1* present/*GSTP1* IIe/IIe: *P* = 0.014, all risk genotypes vs. *GSTT1* present/*GSTP1* IIe/IIe: *P* = 0.045, *GSTT1* null/*GSTP1* val* vs. *GSTT1* null/*GSTP1* IIe/IIe *+ GSTT1* present/GSTP1 Val* + GSTT1 present/*GSTP1* IIe/IIe: *P =* 0.017), and the combined effects of *GSTM1*, *GSTT1*, and *GSTP1* IIe105Val polymorphisms (all two high-risk genotype vs. *GSTM1* present/*GSTT1* present/*GSTP1* IIe/IIe: *P* = 0.043, *GSTM1* null/*GSTT1* null/*GSTP1* Val * vs. *GSTM1* present/*GSTT1* present/*GSTP1* IIe/IIe: *P* = 0.019, *GSTM1* null/*GSTT1* null/*GSTP1* Val* vs. *GSTM1* present/*GSTT1* present/*GSTP1* IIe/IIe + all one high risk genotypes + all two high risk genotypes: *P* = 0.036). S8–S19 Figs list the funnel plots of the nonparametric ‘trim and fill’ method. No significant association was observed for the combined effects of *GSTM1* and *GSTT1* polymorphisms (*− −* vs. *+ +*: OR = 1.19, 95% CI = 0.92–1.52, (*+ −*) + (*− +*) vs. (*+ +*): OR = 1.09, 95% CI = 0.96–1.23, (*− +*) *+* (*+ −*) + (*− −*) vs. *+ +*: OR = 1.12, 95% CI = 0.97–1.29, *− − vs*. (*− +*) *+* (*+ −*) + (*+ +*): OR = 1.14, 95% CI = 0.95–1.37), the combined effects of *GSTM1* and *GSTP1* IIe105Val (*GSTM1* null/*GSTP1* Val* vs. *GSTM1* present/*GSTP1* IIe/IIe: OR = 1.16, 95% CI = 0.86–1.56, all risk genotypes vs. *GSTM1* present/*GSTP1* IIe/IIe: OR = 1.05, 95% CI = 0.87–1.27), the combined effects of *GSTT1* and *GSTP1* IIe105Val (*GSTT1* null/*GSTP1* val* vs. *GSTT1* present/*GSTP1* IIe/IIe: OR = 1.03, 95% CI = 0.77–1.36, all risk genotypes vs. *GSTT1* present/*GSTP1* IIe/IIe: OR = 1.06, 95% CI = 0.86–1.31, *GSTT1* null/*GSTP1* Val* vs. *GSTT1* null/*GSTP1* IIe/IIe + *GSTT1* present/*GSTP1* Val* + *GSTT1* present/*GSTP1* IIe/IIe: OR = 1.02, 95% CI = 0.83–1.26), and the combined effects of *GSTM1*, *GSTT1*, and *GSTP1* IIe105Val polymorphisms (all two high-risk genotype vs. *GSTM1* present/*GSTT1* present/*GSTP1* IIe/IIe: OR = 1.19, 95% CI = 0.88–1.61, *GSTM1* null/*GSTT1* null/*GSTP1* Val * vs. *GSTM1* present/*GSTT1* present/*GSTP1* IIe/IIe: OR = 1.11, 95% CI = 0.72–1.72, *GSTM1* null/*GSTT1* null/*GSTP1* Val* vs. *GSTM1* present/*GSTT1* present/*GSTP1* IIe/IIe + all one high risk genotypes + all two high risk genotypes: OR = 1.04, 95% CI = 0.74–1.47) on BC risk in all populations.

### Credibility of the current and previous meta-analyses

Statistically significant associations were considered as “positive results” when they met the following criteria [44]: (1) *P* value < 0.05 was observed in at least one of the two genetic model (individual *GSTM1* and *GSTT1* polymorphisms with BC risk (there was no need to meet this condition between *GSTM1* and *GSTT1* polymorphisms and BC risk because they only used null vs. present); (2) FPRP < 0.2; (3) statistical power > 0.8; and (4) *I*^2^ < 50%. Associations were considered to be “less-credible positive results” if they did not meet the above criteria. Tables 13 and 14 list the statistically significant association, *I*^2^ value, statistical power and FPRP value for the current and previous meta-analyses, respectively. We identified “less-credible positive results” for the current and previous meta-analyses on the basis of above criteria.

**Table 13:** False-positive report probability values for the current meta-analysis.

Variables	OR (95% CI)	*I*^2^ (%)	Statistical power		Prior probability of 0.001	
			0R = 1.2	OR = 1.5	0R = 1.2	OR = 1.5
*GSTM1* (null vs. present)						
Overall	1.12 (1.06–1.19)	**59.9**	0.987	1.000	0.201	**0.199**
Asian	1.20 (1.05–1.38)	66.5	0.500	0.999	0.955	0.914
PB	1.15 (1.06–1.25)	69.1	0.841	1.000	0.547	0.504
Healthy women	1.21 (1.06–1.39)	67.7	0.453	0.999	0.940	0.870
Cancer-free women	1.07 (1.00–1.13)	46.6	1.000	1.000	0.938	0.938
Cancer-free patients	1.50 (1.05–2.14)	63.7	0.109	0.500	0.996	0.981
Matching	1.07 (1.02–1.13)	28.7	1.000	1.000	0.938	0.938
Non-matching	1.19 (1.05–1.34)	72.0	0.555	1.000	0.880	0.803
≥ 200	1.09 (1.03–1.16)	62.2	0.999	1.000	0.869	0.869
< 200	1.41 (1.14–1.74)	38.6	0.066	0.718	0.954	0.655
> 10	1.07 (1.03–1.11)	0.0	1.000	1.000	0.232	0.232
≤10	1.18 (1.05–1.32)	69.4	0.616	1.000	0.861	0.792
Postmenopausal	1.17 (1.05–1.30)	50.1	0.681	1.000	0.837	0.777
Premenopausal	1.18 (1.01–1.38)	48.3	0.583	0.999	0.985	0.975
Only studies with high quality, matching, and genotyping examination done bindly or quality control						
Yes	1.06 (1.02–1.11)	0.0	1.000	1.000	0.930	0.930
*GSTT1* (null vs. present)						
Overall	1.15 (1.06–1.25)*	63.4	0.841	1.000	0.547	0.504
Caucasian	1.20 (1.07–1.34)	45.7	0.500	1.000	0.766	0.546
HB	1.19 (1.03–1.37)	52.3	0.546	0.999	0.966	0.939
Healthy women	1.32 (1.09–1.60)	68.5	0.166	0.904	0.966	0.838
Cancer-free patients	1.59 (1.22–2.07)	34.9	0.018	0.333	0.969	0.631
Matching	1.09 (1.00–1.19)	51.3	0.984	1.000	0.982	0.982
Non-matching	1.24 (1.04–1.46)	71.9	0.401	0.927	0.996	0.991
≥ 200	1.13 (1.04–1.23)	66.9	0.918	1.000	0.982	0.982
< 200	1.34 (1.08–1.65)	30.0	0.149	0.856	0.975	0.872
≤10	1.25 (1.08–1.44)	64.6	0.286	0.994	0.875	0.667
Premenopausal	1.31 (1.02–1.67)	43.6	0.239	0.863	0.992	0.971
*GSTP1* (Val/Val vs. IIe/IIe)						
Asian	1.30 (1.08–1.57)	0.0	0.203	0.931	0.969	0.873
Indian	1.58 (1.01–2.47)	57.2	0.114	0.410	0.997	0.991
*GSTP1* (IIe/Val vs. IIe/IIe)						
HB	1.14 (1.01–1.30)	34.0	0.778	1.000	0.985	0.981
Matching						
Non-matching	1.18 (1.06–1.31)	56.1	0.624	1.000	0.754	0.656
≤10	1.15 (1.04–1.27)	0.0	0.800	1.000	0.878	0.852
*GSTP1* (Val/Val vs. IIe/IIe + IIe/Val)						
Asian	1.25 (1.04–1.51)	0.0	0.336	0.971	0.984	0.955
Indian	1.45 (1.14–1.85)	36.4	0.064	0.607	0.978	0.821
*GSTP1* (Val/Val + IIe/Val vs. IIe/IIe)						
Asian	1.12 (1.04–1.20)	33.8	0.975	1.000	0.568	0.562
HB	1.11 (1.01–1.21)	29.3	0.962	1.000	0.948	0.947
Non-matching	1.16 (1.04–1.30)	65.1	0.720	1.000	0.937	0.914
≤10	1.12 (1.03–1.23)	25.2	0.926	1.000	0.950	0.947
*GSTP1* (Val vs. IIe)						
Asian	1.12 (1.05–1.19)	**42.6**	0.987	1.000	0.201	**0.199**
Non-matching	1.15 (1.04–1.27)	73.8	0.800	1.000	0.878	0.852
≤10	1.12 (1.01–1.24)	47.0	0.968	1.000	0.970	0.967
The combined effects of *GSTM1* and *GSTT1* polymorphisms						
+ − vs. + +						
Patients	1.74 (1.03–2.95)	–	0.084	0.291	0.998	0.993
− + vs. + +						
Overall	1.18 (1.03–1.35)	58.6	0.597	1.000	0.964	0.941
Indian	1.70 (1.09–2.64)	52.9	0.060	0.289	0.997	0.984
PB	1.26 (1.01–1.56)	77.9	0.327	0.945	0.990	0.973
≥ 200	1.18 (1.03–1.36)	62.7	0.592	1.000	0.974	0.957
≤10	1.36 (1.07–1.72)	62.3	0.148	0.793	0.986	0.928
Non-matching	1.47 (1.07–2.01)	74.8	0.102	0.550	0.994	0.966
− − vs. + +						
Overall	1.65 (1.31–2.07)	73.8	0.003	0.205	0.835	0.068
Asian	2.06 (1.10–3.84)	84.4	0.044	0.159	0.998	0.993
Caucasian	1.93 (1.31–2.83)	67.2	0.007	0.098	0.990	0.885
HB	1.58 (1.21–2.06)	0.0	0.021	0.351	0.972	0.674
PB	1.40 (1.08–1.82)	65.5	0.125	0.697	0.990	0.945
Healthy women	2.05 (1.28–3.26)	70.2	0.012	0.093	0.995	0.963
Cancer-free women	1.68 (1.23–2.29)	74.9	0.017	0.237	0.984	0.813
≥ 200	1.56 (1.24–1.96)	74.7	0.012	0.368	0.917	0.267
< 200	3.82 (1.76–8.29)	0.0	0.002	0.009	0.998	0.987
> 10	1.23 (1.01–1.50)	58.6	0.404	0.975	0.990	0.977
≤10	2.45 (1.58–3.81)	72.9	0.001	0.015	0.989	0.825
Matching	1.33 (1.10–1.60)	40.3	0.138	0.899	0.948	0.735
Non-matching	2.30 (1.38–3.82)	85.0	0.006	0.049	0.995	0.963
Postmenopausal	1.49 (1.14–1.94)	0.0	0.054	0.520	0.983	0.855
(+ −) + (− +) vs. + +						
Overall	1.17 (1.05–1.30)	55.9	0.681	1.000	0.837	0.777
Indian	1.48 (1.19–1.84)	37.1	0.030	0.548	0.934	0.432
Healthy women	1.31 (1.04–1.65)	57.2	0.228	0.875	0.990	0.961
≥ 200	1.15 (1.04–1.28)	55.7	0.782	1.000	0.931	0.913
≤10	1.36 (1.09–1.69)	62.4	0.129	0.812	0.977	0.872
Non-matching	1.35 (1.09–1.67)	71.2	0.139	0.834	0.976	0.872
(+ −) + (− +) + (− −) vs. + +						
Overall	1.27 (1.12–1.43)	68.0	0.175	0.997	0.311	0.073
Caucasian	1.36 (1.10–1.68)	71.1	0.123	0.818	0.972	0.841
Indian	1.54 (1.02–2.32)	60.0	0.116	0.450	0.997	0.989
HB	1.22 (1.01–1.47)	0.0	0.431	0.985	0.988	0.974
PB	1.23 (1.04–1.45)	73.7	0.384	0.991	0.973	0.932
Healthy women	1.43 (1.10–1.85)	67.8	0.091	0.642	0.986	0.910
Cancer-free women	1.23 (1.04–1.46)	70.6	0.389	0.988	0.979	0.948
≥ 200	1.25 (1.11–1.41)	69.3	0.253	0.998	0.527	0.220
≤10	1.53 (1.19–1.96)	72.9	0.027	0.438	0.966	0.636
Non-matching	1.51 (1.20–1.91)	80.0	0.028	0.478	0.955	0.551
− − vs. (+ +) + (+ −) + (− +)						
Overall	1.41 (1.19–1.68)	65.9	0.036	0.756	0.773	0.138
Asian	1.53 (1.01–2.31)	82.5	0.124	0.462	0.997	0.989
Caucasian	1.61 (1.22–2.12)	46.7	0.018	0.307	0.974	0.693
HB	1.42 (1.16–1.75)	7.0	0.057	0.696	0.946	0.590
PB	1.22 (1.01–1.49)	54.0	0.436	0.979	0.992	0.981
Healthy women	1.67 (1.25–2.23)	49.7	0.013	0.l233	0.976	0.686
Cancer-free women	1.46 (1.13–1.88)	72.9	0.064	0.583	0.981	0.852
≥ 200	1.34 (1.13–1.60)	68.0	0.111	0.894	0.916	0.576
< 200	2.77 (1.61–4.78)	0.0	0.001	0.014	0.995	0.948
≤10	1.82 (1.36–2.43)	57.7	0.002	0.095	0.954	0.340
Matching	1.25 (1.05–1.48)	46.5	0.318	0.983	0.968	0.907
Non-matching	1.81 (1.24–2.62)	78.4	0.015	0.160	0.991	0.912
Postmenopausal	1.25 (1.02–1.53)	23.9	0.346	0.961	0.989	0.969
Only studies with high quality, matching, and genotyping examination done bindly or quality control						
− − vs. + +						
Overall	1.27 (1.02–1.59)	53.0	0.310	0.927	0.992	0.976
Caucasian	1.58 (1.10–2.29)	–	0.073	0.392	0.995	0.976
Postmenopausal	1.50 (1.13–2.00)	10.0	0.064	0.500	0.989	0.920
− − vs. (+ +) + (+ −) + (− +)						
Caucasian	1.58 (1.11–2.24)	–	0.061	0.385	0.994	0.964
Postmenopausal	1.29 (1.03–1.61)	46.1	0.261	0.909	0.989	0.964
The combined effects of *GSTM1* and *GSTP1* IIe/Val polymorphisms						
*GSTM1* Null/ *GSTP1* IIe/IIe vs. *GSTM1* Present/ *GSTP1* IIe/IIe						
Caucasian	1.37 (1.03–1.83)	0.0	0.185	0.730	0.994	0.978
< 200	2.07 (1.09–3.94)	0.0	0.048	0.163	0.998	0.994
Non-matching	1.48 (1.14–1.91)	0.0	0.054	0.541	0.980	0.827
Yes (HWE)	1.16 (1.02–1.33)	0.0	0.686	1.000	0.980	0.971
(*GSTM1* Null/ *GSTP1* IIe/IIe + *GSTM1* Present/*GSTP1* Val*) vs. *GSTM1* Present/ *GSTP1* IIe/IIe						
Overall	1.14 (1.00–1.31)	52.8	0.765	1.000	0.988	0.985
Caucasian	1.60 (1.29–1.98)	19.5	0.004	0.276	0.791	0.053
Non-matching	1.46 (1.14–1.87)	55.1	0.060	0.585	0.978	0.823
*GSTM1* Null/ GSTP1 Val* vs. *GSTM1* Present/ *GSTP1* IIe/IIe						
Overall	1.58 (1.21–2.06)	82.2	0.021	0.351	0.972	0.674
Asian	1.23 (1.03–1.46)	0.0	0.389	0.988	0.979	0.948
Caucasian	2.11 (1.21–3.66)	79.6	0.022	0.112	0.997	0.986
NPB	1.41 (1.17–1.70)	7.0	0.046	0.742	0.875	0.300
PB	1.74 (1.10–2.76)	91.5	0.057	0.264	0.997	0.986
Healthy women	1.86 (1.32–2.62)	72.7	0.006	0.109	0.984	0.779
≥ 200	1.52 (1.15–2.01)	83.9	0.049	0.463	0.986	0.877
< 200	2.36 (1.32–4.22)	0.0	0.011	0.063	0.997	0.984
> 10	1.61 (1.11–2.35)	88.7	0.064	0.357	0.995	0.974
≤10	1.47 (1.18–1.83)	26.5	0.035	0.572	0.942	0.497
Non-matching	2.30 (1.44–3.69)	81.2	0.003	0.038	0.994	0.935
Yes (HWE)	1.74 (1.17–2.60)	88.8	0.035	0.234	0.995	0.967
All risk genotypes vs. *GSTM1* Present/ *GSTP1* IIe/IIe						
Overall	1.28 (1.08–1.52)	73.5	0.231	0.965	0.955	0.835
Caucasian	1.77 (1.26–2.48)	58.6	0.012	0.168	0.987	0.843
NPB	1.24 (1.01–1.54)	55.1	0.383	0.957	0.993	0.982
PB	1.33 (1.01–1.74)	83.9	0.227	0.810	0.994	0.979
Healthy women	1.44 (1.10–1.89)	72.6	0.094	0.616	0.989	0.933
≥ 200	1.26 (1.05–1.50)	76.2	0.292	0.975	0.970	0.906
< 200	1.64 (1.01–2.66)	0.0	0.103	0.359	0.998	0.992
> 10	1.23 (1.00–1.52)	79.0	0.410	0.967	0.993	0.983
≤10	1.39 (1.02–1.89)	60.7	0.174	0.686	0.995	0.981
Non-matching	1.74 (1.26–2.39)	76.6	0.011	0.180	0.983	0.777
Yes (HWE)	1.41 (1.08–1.83)	82.7	0.113	0.679	0.989	0.935
*GSTM1* Null/ GSTP1 Val* vs. (*GSTM1* Null/ *GSTP1* IIe/IIe + *GSTM1* Present/*GSTP1* Val* + *GSTM1* Present/ *GSTP1* IIe/IIe)						
Overall	1.40 (1.12–1.75)	82.4	0.088	0.728	0.973	0.811
Asian	1.21 (1.04–1.41)	0.0	0.458	0.997	0.970	0.936
Indian	2.02 (1.14–3.59)	78.3	0.038	0.155	0.998	0.991
NPB	1.19 (1.02–1.38)	35.3	0.544	0.999	0.975	0.955
PB	1.58 (1.08–2.29)	91.3	0.073	0.392	0.995	0.976
Cancer-free women	1.57 (1.15–2.15)	77.9	0.047	0.388	0.991	0.927
≥ 200	1.35 (1.07–1.70)	84.1	0.158	0.815	0.985	0.929
< 200	1.99 (1.24–3.20)	0.0	0.018	0.122	0.996	0.974
> 10	1.51 (1.11–2.06)	88.5	0.074	0.483	0.992	0.951
Non-matching	1.76 (1.13–2.76)	86.6	0.048	0.243	0.997	0.983
Yes (HWE)	1.47 (1.06–2.04)	88.8	0.112	0.548	0.995	0.975
The combined effects of *GSTT1* and *GSTP1* IIe/Val polymorphisms						
GSTT1 Present/GSTP1 Val* vs. *GSTT1* Present/ *GSTP1* IIe/IIe						
PB	1.48 (1.02–2.15)	88.5	0.136	0.528	0.997	0.987
(*GSTT1* Null/ *GSTP1* IIe/IIe + *GSTT1* Present/*GSTP1* Val*) vs. *GSTT1* Present/ *GSTP1* IIe/IIe						
PB	1.40 (1.02–1.93)	86.6	0.173	0.663	0.996	0.984
> 10	1.28 (1.00–1.62)	81.9	0.296	0.907	0.993	0.978
*GSTT1* Null/ GSTP1 Val* vs. *GSTT1* Present/ *GSTP1* IIe/IIe						
Overall	1.44 (1.10–1.88)	68.4	0.090	0.618	0.988	0.922
Caucasian	2.09 (1.15–3.80)	60.8	0.034	0.138	0.998	0.991
NPB	1.51 (1.18–1.93)	0.0	0.033	0.479	0.968	0.675
Healthy women	1.85 (1.21–2.82)	56.8	0.022	0.165	0.995	0.962
≥ 200	1.36 (1.03–1.79)	69.9	0.186	0.758	0.993	0.974
< 200	2.30 (1.16–4.54)	19.4	0.030	0.109	0.998	0.993
≤10	1.39 (1.04–1.87)	0.0	0.166	0.693	0.994	0.977
Non-matching	2.12 (1.37–3.28)	51.6	0.005	0.060	0.993	0.925
All risk genotypes vs. *GSTT1* Present/ *GSTP1* IIe/IIe						
Overall	1.23 (1.03–1.48)	76.3	0.397	0.982	0.986	0.966
PB	1.42 (1.02–1.99)	88.9	0.164	0.625	0.996	0.985
> 10	1.32 (1.02–1.69)	84.8	0.225	0.845	0.992	0.970
Non-matching	1.54 (1.05–2.28)	83	0.106	0.448	0.997	0.986
*GSTT1* Null/ GSTP1 Val* vs. (*GSTT1* null/ *GSTP1* IIe/IIe + *GSTT1* Present/*GSTP1* Val* + *GSTT1* Present/ *GSTP1* IIe/IIe)						
Overall	1.26 (1.03–1.54)	52.7	0.317	0.956	0.987	0.962
Caucasian	1.50 (1.11–2.02)	8.4	0.071	0.500	0.991	0.938
Indian	1.55 (1.12–2.15)	0.0	0.063	0.422	0.993	0.953
NPB	1.48 (1.18–1.85)	0.0	0.136	0.528	0.997	0.987
Healthy women	1.52 (1.21–1.92)	0.0	0.024	0.456	0.949	0.493
< 200	1.87 (1.05–3.31)	17.2	0.064	0.225	0.998	0.993
≤10	1.38 (1.06–1.81)	0.0	0.156	0.727	0.992	0.965
Non-matching	1.60 (1.25–2.04)	0.0	0.010	0.301	0.936	0.331
The combined effects of *GSTM1* present/null, *GSTT1* present/null and *GSTP1* present/null						
*M1* Null/*T1* Present/*P1* IIe/IIe vs. *M1* Present/*T1* Present/*P1* IIe/IIe						
Non-matching	1.39 (1.03–1.86)	0.0	0.161	0.696	0.994	0.975
*M1* Present/*T1* Present/*P1* Val ^1^ vs. *M1* Present/*T1* Present/*P1* IIe/IIe						
Caucasian	1.46 (1.07–1.99)	47.3	0.107	0.568	0.994	0.967
All one high-risk genotype vs. vs. *M1* Present/*T1* Present/*P1* IIe/IIe						
Caucasian	1.36 (1.03–1.77)	0.0	0.176	0.767	0.992	0.967
Non-matching	1.18 (1.00–1.39)	0.0	0.580	0.998	0.988	0.979
M1 Null/T1 Null/P1 IIe/IIe vs. *M1* Present/*T1* Present/*P1* IIe/IIe						
Overall	1.44 (1.00–2.06)	52.9	0.159	0.588	0.997	0.987
< 200	5.58 (1.96–15.89)	46.6	0.002	0.007	0.998	0.995
Yes (HWE)	1.40 (1.03–1.90)	35.7	0.161	0.671	0.995	0.979
Only studies with high quality, matching, HWE, and genotyping examination done bindly or quality control						
Yes	1.84 (1.22–2.77)	0.0	0.020	0.164	0.994	0.955
M1 Null/T1 Present/P1 Val ^1^ vs. *M1* Present/*T1* Present/*P1* IIe/IIe						
Overall	1.54 (1.08–2.18)	81.1	0.080	0.441	0.995	0.971
PB	1.94 (1.08–3.48)	90.7	0.054	0.194	0.998	0.993
≥ 200	1.49 (1.02–2.19)	84.2	0.135	0.514	0.997	0.988
> 10	1.72 (1.01–2.93)	89.3	0.093	0.307	0.998	0.993
Non-matching	2.50 (1.29–4.84)	81.0	0.015	0.065	0.998	0.990
Yes (HWE)	1.78 (1.06–3.01)	89.0	0.071	0.262	0.998	0.992
M1 Present/T1 Null/P1 Val ^1^ vs. *M1* Present/*T1* Present/*P1* IIe/IIe						
Non-matching	1.57 (1.04–2.37)	0.0	0.100	0.414	0.997	0.987
All two high-risk genotype *vs*. *M1* Present/*T1* Present/*P1* IIe/IIe						
Overall	1.41 (1.08–1.83)	77.7	0.113	0.679	0.989	0.935
Caucasian	2.07 (1.06–4.04)	78.4	0.055	0.173	0.99	0.995
PB	1.71 (1.06–2.75)	89.1	0.072	0.294	0.997	0.989
≥ 200	1.34 (1.02–1.76)	79.6	0.214	0.791	0.994	0.978
< 200	2.27 (1.16–4.45)	17.0	0.032	0.114	0.998	0.993
> 10	1.45 (1.01–2.09)	85.5	0.155	0.572	0.997	0.988
Non-matching	2.00 (1.18–3.38)	83.1	0.028	0.141	0.997	0.986
Yes (HWE)	1.61 (1.03–2.50)	87.0	0.095	0.376	0.997	0.989
M1 Null/T1 Null/P1 Val ^1^ vs. *M1* Present/*T1* Present/*P1* IIe/IIe						
Overall	1.79 (1.19–2.67)	72.1	0.025	0.193	0.994	0.957
Caucasian	2.64 (1.23–5.66)	61.4	0.021	0.073	0.998	0.994
Indian	3.10 (1.77–5.42)	46.1	<0.001	0.005	0.994	0.930
NPB	1.83 (1.30–2.58)	0.0	0.008	0.128	0.986	0.815
Healthy women	2.30 (1.62–3.26)	42.0	<0.001	0.008	0.957	0.260
≥ 200	1.60 (1.06–2.41)	72.5	0.084	0.379	0.997	0.985
< 200	4.37 (1.75–10.92)	0.0	0.003	0.011	0.998	0.993
> 10	1.72 (1.00–2.94)	80.3	0.094	0.308	0.998	0.994
≤10	1.79 (1.19–2.70)	26.0	0.028	0.200	0.995	0.965
Non-matching	2.99 (1.66–5.41)	55.1	0.001	0.011	0.996	0.963
Yes (HWE)	1.87 (1.00–3.50)	80.3	0.083	0.245	0.998	0.995
M1 Null/T1 Null/P1 Val ^1^ vs. (All one high-risk genotypes + All two high-risk genotype + *M1* Present/*T1* Present/*P1* IIe/IIe)						
Overall	1.51 (1.10–2.06)	63.3	0.074	0.483	0.992	0.951
Caucasian	1.63 (1.13–2.36)	43.3	0.052	0.330	0.995	0.967
Indian	2.58 (1.51–4.40)	0.0	0.002	0.023	0.995	0.956
NPB	1.75 (1.29–2.36)	29.2	0.007	0.156	0.973	0.610
Healthy women	1.99 (1.46–2.70)	27.3	0.001	0.035	0.944	0.221
≥ 200	1.37 (1.00–1.88)	63.0	0.206	0.713	0.996	0.986
< 200	3.14 (1.47–6.72)	0.0	0.007	0.029	0.998	0.991
≤10	1.56 (1.08–2.24)	33.6	0.078	0.410	0.995	0.975
Non-matching	2.08 (1.35–3.21)	34.7	0.006	0.070	0.993	0.931

Val ^1^:IIe/Val + Val/Val, PB: population-based studies; HB: hospital-based studies; HWE: Hardy-Weinberg equilibrium; NPB: no population-based studies

**Table 14:** False-positive report probability values for the previous meta-analyses on *GSTM1*, *GSTT1*, and *GSTP1* IIe105Val polymorphisms with breast cancer risk.

Author	Gene	Model	n	Case/Control	Variable	OR (95% CI)	*I*^2^ (%)	Statistical power		Prior probability of 0.001	
								0R = 1.2	OR = 1.5	0R = 1.2	OR = 1.5
Xue [15] 2016	*GSTM1*	null vs. present	17	5,323/7,196	Chinese	1.28 (1.09–1.51)	NA	0.222	0.970	0.939	0.778
Xue [15] 2016	*GSTM1*	null vs. present	8	NA	HB	1.55 (1.20–2.00)	NA	0.025	0.400	0.968	0.652
Xue [15] 2016	*GSTM1*	null vs. present	11	NA	Mainland China	1.42 (1.12–1.81)	NA	0.087	0.671	0.982	0.873
Kuang [16] 2016	*GSTP1*	Val/Val vs. IIe/IIe	15	NA	HB	1.28 (1.10–1.48)	NA	0.192	0.984	0.818	0.466
Kuang [16] 2016	*GSTP1*	Val/Val + IIe/Val vs. IIe/IIe	15	NA	HB	1.10 (1.02–1.18)	NA	0.992	1.000	0.887	0.886
Kuang [16] 2016	*GSTP1*	Val/Val vs. IIe/IIe + IIe/Val	15	NA	HB	1.22 (1.06–1.41)	NA	0.411	0.997	0.945	0.877
Kuang [16] 2016	*GSTP1*	Val/Val vs. IIe/IIe	12	NA	Asian	1.41 (1.06–1.88)	NA	0.136	0.663	0.993	0.967
Kuang [16] 2016	*GSTP1*	IIe/Val vs. IIe/IIe	12	NA	Asian	1.08 (1.00–1.16)	NA	0.998	1.000	0.972	0.972
Kuang [16] 2016	*GSTP1*	Val/Val + IIe/Val vs. IIe/IIe	12	NA	Asian	1.11 (1.04–1.19)	NA	0.986	1.000	0.769	0.767
Song [17] 2016	*GSTM1*	null vs. present	7	NA	Asian	1.17 (1.04–1.32)	41.4	0.660	1.000	0.942	0.915
Song [17] 2016	*GSTT1*	null vs. present	6	NA	Asian	1.19 (1.01–1.41)	43.3	0.555	1.000	0.880	0.803
Song [17] 2016	*GSTP1*	Val/Val vs. IIe/IIe + IIe/Val	6	NA	Caucasian	1.16 (1.01–1.34)	25.4	0.677	1.000	0.985	0.978
Tang [18] 2015	*GSTT1*	null vs. present	9	2,770/3,775	East Asian	1.20 (1.00–1.45)	62	0.500	0.990	0.992	0.983
Tang [18] 2015	*GSTT1*	null vs. present	5	531/611	Premenopausal	1.45 (1.10–1.93)	0	0.097	0.592	0.991	0.948
Tang [18] 2015	*GSTT1*	null vs. present	15	2,580/2,587	HB	1.30 (1.07–1.59)	53	0.218	0.918	0.980	0.921
Tang [18] 2015	*GSTM1*	null vs. present	27	7,409/9,301	Asian	1.18 (1.04–1.33)	65	0.608	1.000	0.917	0.870
Tang [18] 2015	*GSTM1*	null vs. present	13	4,699/5,881	East Asian	1.14 (1.01–1.27)	41	0.824	1.000	0.955	0.946
Tang [18] 2015	*GSTM1*	null vs. present	7	1,459/1,689	Premenopausal	1.51 (1.23–1.86)	40	0.015	0.475	0.874	0.183
Tang [18] 2015	*GSTM1*	null vs. present	17	3,856/3,719	HB	1.32 (1.11–1.56)	64	0.132	0.933	0.895	0.546
Tang [18] 2015	*GSTP1*	Val/Val vs. IIe/IIe + IIe/Val	20	8,557/9,544	Asian	1.23 (1.07–1.41)	70	0.362	0.998	0.891	0.748
Tang [18] 2015	*GSTP1*	Val/Val vs. IIe/IIe + IIe/Val	7	6,108/6,514	East Asian	1.15 (1.03–1.28)	35	0.782	1.000	0.931	0.913
Tang [18] 2015	*GSTP1*	Val/Val vs. IIe/IIe + IIe/Val	12	2,884/2,591	HB	1.38 (1.03–1.84)	78	0.170	0.715	0.994	0.975
Tang [18] 2015	*GSTP1*	Val/Val vs. IIe/IIe + IIe/Val	8	5,673/6,953	PB	1.10 (1.02–1.19)	0	0.985	1.000	0.947	0.946
Tang [18] 2015	*GSTP1*	Val vs. IIe	15	15,754/17,036	Asian	1.30 (1.12–1.51)	82	0.147	0.969	0.801	0.380
Tang [18] 2015	*GSTP1*	Val vs. IIe	5	11,738/12,156	East Asian	1.14 (1.04–1.26)	42	0.842	1.000	0.924	0.911
Tang [18] 2015	*GSTP1*	Val vs. IIe	5	2,440/2,858	South Asian	1.44 (1.00–2.07)	87	0.162	0.587	0.997	0.988
Tang [18] 2015	*GSTP1*	Val vs. IIe	8	4,750/4,008	HB	1.58 (1.14–2.19)	88	0.049	0.378	0.992	0.941
Tang [18] 2015	*GSTP1*	Val vs. IIe	7	11,004/13,028	PB	1.11 (1.04–1.19)	0	0.986	1.000	0.769	0.767
Xiao [19] 2015	*GSTT1*	null vs. present	13	3,387/5,085	Chinese	1.31 (1.02–1.67)	NA	0.239	0.863	0.992	0.971
Xiao [19] 2015	*GSTT1*	null vs. present	5	NA	HB	1.90 (1.44–2.49)	NA	<0.001	0.043	0.883	0.070
Xiao [19] 2015	*GSTT1*	null vs. present	3	NA	Northern Chinese	2.67 (1.81–3.94)	NA	<0.001	0.002	0.964	0.291
Wan [20] 2014	*GSTM1*	null vs. present	15	5,176/5,890	Chinese	1.34 (1.12–1.60)	77	0.111	0.894	0.916	0.576
Wan [20] 2014	*GSTM1*	null vs. present	12	NA	Southern Chinese	1.14 (1.01–1.28)	39	0.807	1.000	0.971	0.964
Wan [20] 2014	*GSTM1*	null vs. present	3	NA	Northern Chinese	2.65 (2.04–3.34)	39	0.001	0.001	0.999	0.999
Wan [20] 2014	*GSTM1*	null vs. present	11	NA	HB	1.34 (1.12–1.60)	50	0.113	0.892	0.921	0.596
Liu [21] 2013	*GSTP1*	Val vs. IIe	9	NA	Asian	1.10 (1.04–1.17)	NA	0.997	1.000	0.712	0.711
Liu [21] 2013	*GSTP1*	Val/Val vs. IIe/IIe + IIe/Val	9	NA	Asian	1.36 (1.14–1.62)	NA	0.080	0.864	0.876	0.398
Liu [21] 2013	*GSTP1*	Val/Val vs. IIe/IIe + IIe/Val	8	NA	Asian	1.28 (1.02–1.62)	NA	0.296	0.907	0.993	0.978
Liu [21] 2013	*GSTP1*	Val vs. IIe	13	NA	HB	1.11 (1.05–1.19)	NA	0.986	1.000	0.769	0.767
Liu [21] 2013	*GSTP1*	Val/Val vs. IIe/IIe + IIe/Val	13	NA	HB	1.32 (1.12–1.55)	NA	0.122	0.941	0.852	0.428
Chen [22] 2011	*GSTT1*	null vs. present	48	17,254/21,163	Overall	1.14 (1.05–1.23)	NA	0.907	1.000	0.444	0.420
Chen [22] 2011	*GSTT1*	null vs. present	32	NA	Caucasian	1.19 (1.08–1.31)	NA	0.568	1.000	0.405	0.279
Chen [22] 2011	*GSTT1*	null vs. present	19	NA	HB	1.18 (1.06–1.32)	NA	0.616	1.000	0.861	0.792
Chen [22] 2011	*GSTT1*	null vs. present	27	NA	PB	1.12 (1.01–1.24)	NA	0.908	1.000	0.970	0.967
Economopoulos and Lu [23] 2010	*GSTP1*	Val/Val vs. IIe/IIe + IIe/Val	NA	NA	Asian	1.36 (1.13–1.63)	NA	0.088	0.856	0.909	0.505
Economopoulos and Lu [23] 2010	*GSTP1*	Val/Val vs. IIe/IIe	NA	NA	HB	1.32 (1.07–1.63)	NA	0.188	0.883	0.981	0.918
Economopoulos and Lu [23] 2010	*GSTP1*	Val/Val vs. IIe/IIe + IIe/Val	NA	NA	HB	1.24 (1.01–1.51)	NA	0.372	0.971	0.989	0.971
Economopoulos and Lu [23] 2010	*GSTP1*	Val/Val + IIe/Val vs. IIe/IIe	NA	NA	HB	1.14 (1.01–1.27)	NA	0.824	1.000	0.955	0.946
Lu [24] 2011	*GSTP1*	Val/Val vs. IIe/IIe	8	NA	Asian	1.27 (1.02–1.83)	NA	0.380	0.814	0.998	0.996
Lu [24] 2011	*GSTP1*	Val/Val vs. IIe/IIe + IIe/Val	8	NA	Asian	1.42 (1.20–1.69)	NA	0.029	0.731	0.730	0.097
Lu [24] 2011	*GSTP1*	Val/Val vs. IIe/IIe	13	NA	HB	1.38 (1.16–1.63)	NA	0.050	0.837	0.750	0.152
Lu [24] 2011	*GSTP1*	Val/Val vs. IIe/IIe + IIe/Val	13	NA	HB	1.31 (1.12–1.55)	NA	0.153	0.943	0.915	0.637
Lu [24] 2011	*GSTP1*	Val/Val + IIe/Val vs. IIe/IIe	13	NA	HB	1.10 (1.02–1.19)	NA	0.985	1.000	0.947	0.946
Qiu [25] 2010	*GSTM1*	null vs. present	59	20,993/25,288	Overall	1.10 (1.04–1.16)	NA	0.999	1.000	0.303	0.303
Qiu [25] 2010	*GSTM1*	null vs. present	32	NA	Caucasian	1.05 (1.00–1.10)	NA	1.000	1.000	0.975	0.975
Qiu [25] 2010	*GSTM1*	null vs. present	15	NA	Asian	1.21 (1.08–1.35)	NA	0.441	1.000	0.593	0.391
Qiu [25] 2010	*GSTM1*	null vs. present	27	NA	PB	1.11 (1.03–1.20)	NA	0.975	1.000	0.899	0.897
Qiu [25] 2010	*GSTM1*	null vs. present	17	NA	Postmenopausal	1.15 (1.04–1.28)	NA	0.782	1.000	0.931	0.913
Sergentanis [26] 2010	*GSTT1*	null vs. present	41	16,589/19,995	Overall	1.11 (1.04–1.20)	NA	0.975	1.000	0.654	0.639
Sergentanis [26] 2010	*GSTT1*	null vs. present	33	14,139/16,465	Non-Chinese	1.13 (1.04–1.22)	29.5	0.938	1.000	0.654	0.639
Sergentanis [26] 2010	*GSTP1*	Val/Val vs. IIe/IIe	5	4,256/5,173	Chinese	1.30 (1.02–1.65)	NA	0.255	0.880	0.992	0.972
Sergentanis [26] 2010	*GSTP1*	Val/Val vs. IIe/IIe + IIe/Val	5	4,256/5,173	Chinese	1.27 (1.01–1.61)	NA	0.320	0.915	0.993	0.981
Sull [27] 2004	*GSTM1*	null vs. present	10	2,005/2,282	Postmenopausal	1.19 (1.05–1.34)	NA	0.555	1.000	0.880	0.803
Sull [27] 2004	*GSTM1*	null vs. present	15	2,682/2,813	*GSTM1* deficiency (%) ^1^ < 50.4	1.20 (1.08–1.34)	NA	0.500	1.000	0.706	0.546
Egan [29] 2004	*GSTM1*	null vs. present	11	2,521/2,963	Postmenopausal	1.14 (1.02–1.27)	NA	0.824	1.000	0.955	0.946
Egan [29] 2004	*GSTT1*	null vs. present	15	4,873/5,245	All women	1.11 (1.01–1.22)	NA	0.947	1.000	0.970	0.968

^1^ Median value was used to dichotomize the characteristics; PB: population-based studies; HB: hospital-based studies; NA: not available

## Discussion

A meta-analysis involving 101 publications was done to evaluate the relationship between individual and combined effects of *GSTM1*, *GSTT1*, and *GSTP1* polymorphisms on BC risk. We also used FPRP test and Venice criteria to re-analyze the previously published systematic meta-analyses. As far as we know, this is the first meta-analysis to investigate whether there was an increased BC risk for the combined effects of *GSTM1* present/null, *GSTT1* present/null, and *GSTP1* IIe105Val polymorphisms.

Among these genes, both *GSTM1* and *GSTT1* genes show deletion polymorphisms (null genotype), which cause the absence of expression and enzyme activity loss. *GSTP1* IIe105Val polymorphism also decreases enzymatic activity. Given the involvement of *GSTs* in deactivating and detoxifying carcinogens, deletions in *GSTM1* and *GSTT1* and IIe105Val polymorphism in *GSTP1* resulting in no enzyme activity may compromise an individual’s ability to deactivate carcinogens, thus increasing risk of cancer. The exact mechanism involved are still mysterious, these combined effects might be due to the involvement of *GSTM1*, *GSTT1*, and *GSTP1* in metabolism. Moreover, each gene expresses an increased risk genotype (*GSTM1* null, *GSTT1* null and *GSTP1* Val/Val), which may be involved in breast cancer susceptibility when more than one are expressed in each individual. Overall, statistically significant increased BC risk was found in any individual and combined effects of the *GSTM1*, *GSTT1* and *GSTP1* polymorphisms. In addition, significant association was also observed in some subgroups for these genes on BC risk. However, when we restrained only high-quality studies, HWE, matching, and genotyping examination performed blindly or with quality control, significantly increased BC risk was found in the overall analysis for *GSTM1* null genotype, all populations, Caucasians, and postmenopausal women for the combined effects of *GSTM1* and *GSTT1* polymorphisms, and overall analysis for the combined effects of *GSTM1*, *GSTT1*, and *GSTP1* IIe105Val polymorphisms. This was an attempt to avoid random errors and confounding bias that sometimes distorted the results of molecular epidemiological studies [45–47]. Furthermore, the current meta-analysis were analyzed by applying several subgroups and different genetic models at the expense of multiple comparisons, under these circumstances, the pooled *P*-value must be adjusted [48]. With regard to the Venice criteria, statistical power and *I*^2^ were important indicator by Ioannidis et al. [49]. Hence, we used FPRP test and Venice criteria to assess positive results. Finally, we identified “less-credible positive results” for the current and previous meta-analyses when we evaluated the credibility of significant associations in the current and previous meta-analyses. Heterogeneity was also observed in the current meta-analysis. The results of meta-regression analysis suggested that source of controls, type of controls and quality score of articles were source of heterogeneity between the combined effects of *GSTM1* and *GSTT1* polymorphisms and BC risk. For the combined effects of *GSTM1* and *GSTP1* IIe105Val polymorphisms, matching was source of heterogeneity in this meta-analysis. Therefore, we should perform subgroup analyses to reduce heterogeneity, because HB, patients, low quality studies and no-matching studies were important confounding bias. In addition, random error and bias were common in the studies with small sample sizes, and the results were unreliable, especially in molecular epidemiological studies [48]. Furthermore, small sample studies were easier to accept if there was a positive report as they tend to yield false-positive results because they may be not rigorous and are often of low-quality. S8–S19 Figs indicates that the asymmetry of the funnel plot was caused by a study with low-quality small samples.

A total of fourteen previous meta-analyses [19–32] between 2004 and 2016 have been published to analyze the individual *GSTM1* present/null, *GSTT1* present/null, and/or *GSTP1* IIe105Val polymorphisms on breast cancer (BC) risk. Table 14 lists the statistically significant association, *I*^2^ value, statistical power and FPRP value for the previous meta-analyses. Xue et al. [19] performed an association of 17 studies involving 5,323 cases and 7,196 controls in Chinese population, and suggested that the *GSTM1* null genotype contributed to an increased CRC risk in Chinese population. Kuang et al. [20] examined 36 studies including 20,615 cases and 20,481 controls to show that the *GSTP1* IIe105Val polymorphism was associated with an increased BC risk in Asians. The examination of 17 studies of *GSTM1* (including 4,046 cases and 5,344 controls), 14 studies of *GSTT1* (including 2,788 cases and 3,686 controls), and 10 studies of *GSTP1* (including 3,233 cases and 3,246 controls) by Song et al. [21] indicated that the *GSTM1* and *GSTT1* null genotypes were associated with an increased BC risk in Asians and the *GSTP1* IIe105Val polymorphism was associated with an increased BC risk in Caucasians. The examination of 27 studies of *GSTM1*, 23 studies of *GSTT1*, and 20 studies of *GSTP1* by Tang et al. [22] indicated that the *GSTM1* and *GSTP1* polymorphisms were associated with an increased BC risk in Asian population, especially in East Asian, while the *GSTT1* polymorphism may be not associated with BC risk. Xiao et al. [23] conducted an association of 13 studies involving 3,387 cases and 5,085 controls in Chinese population, and suggested that the *GSTT1* null genotype contributed to an increased BC risk in Chinese population. Wan et al. [24] identified 15 studies of 5,176 cases and 5,890 controls in Chinese population, and demonstrated that the *GSTM1* null genotype was associated with an increased BC risk in the Chinese population. Liu et al. [25] conducted an association of 35 investigations including 18,665 BC cases and 21,682 controls, and demonstrated that *GSTP1* IIe105Val polymorphism was associated with increased BC risk in Asians. Chen et al. [26] selected 48 studies involving 17,254 cases and 21,163 controls to suggest that the *GSTT1* null genotype may contribute to an increased BC risk in Asians and Caucasians. Economopoulos and Sergentanis [27] assessed the meta-analysis of Lu et al. [28], the results indicated that the *GSTP1* IIe105Val polymorphism was associated with an increased BC risk in Asians. Lu et al. [28] evaluated the association of the *GSTP1* IIe105Val polymorphism with BC risk in all races in 30 published studies (including 15,901 cases and 18,757 controls) indicated that the *GSTM1* null genotype may be associated with an increased risk of BC in Asians. Qiu et al. [29] identified 59 studies of 20,993 cases and 25,288 controls in all populations, and demonstrated that the *GSTM1* null genotype was associated with an increased BC risk in Caucasians and postmenopausal women. The examination of 41 studies of *GSTT1* (16,589 cases and 19,995 controls) and 30 studies of *GSTP1* (16,908 cases and 20,016 controls) by Sergentanis and Economopoulos [30] indicated that the *GSTT1* null genotype and *GSTP1* IIe105Val polymorphisms seemed to be associated with an increased BC risk in a race-specific manner. The finding on *GSTP1* IIe105Val polymorphisms was further investigated because of the small number of Chinese studies. Sull et al. [31] examined 30 studies (including 5,904 cases and 6,459 controls) to assess the *GSTM1* null genotype association with BC risk they found that the *GSTM1* null genotype was associated with an increased BC risk in postmenopausal women. The examination of 19 studies of *GSTM1* (5,950 BC cases and 6,601 controls), 15 studies of *GSTT1* (4,873 BC cases and 5,245 controls), and 10 studies of *GSTP1* (2,136 BC cases and 2,282 controls) by Egan et al. [32] suggested that the *GSTM1* and *GSTT1* null genotypes were associated with an increased BC risk in postmenopausal and all women, respectively. However, quality assessment of the eligible studies was not assessed in 12 previous meta-analyses [19–21, 23, 25–32], source of heterogeneity was not explored in 13 previous meta-analyses [19–32] on the basis of meta-regression analysis, the false-positive report probabilities of statistically significant association and statistical power was not evaluated in all previous meta-analyses [19–32], and *I*^2^ value was not showed in 11 previous meta-analyses [19, 20, 23, 25–32]. Therefore, results of their meta-analyses may be not credible.

This meta-analysis has several advantages over previous meta-analyses [19–32]. First, the sample size was much larger, with 88 studies involving 28,676 BC cases and 32,539 controls assessed for the *GSTM1* null genotype, 67 studies involving 23,092 BC cases and 26,381 controls for the *GSTT1* null genotype, and 56 studies involving 25,331 BC cases and 27,424 controls in all populations. Second, this is the first meta-analysis to investigate the combined effects of these genes in overall population. Third, we evaluated quality assessment of the eligible studies. Forth, we used meta-regression analysis method to explore the source of heterogeneity. Fifth, we collected more detailed data. Sixth, an important sensitivity analysis was conducted on studies that were high-quality, matching, HWE, and or in which genotyping was performed blindly or with quality control. Seventh, we applied FPRP and Venice criteria to investigate the significant association with BC risk. The current meta-analysis also has several limitations. First, only published articles were included in the current meta-analysis, therefore, publication bias may be exist as shown in S8–S19 Figs. Positive results are known to be published more readily than negative ones. If negative results were included, an underestimation of the *GSTM1* null effect may be observed. Second, we did not consider whether the genotype distribution in the controls was in HWE for *GSTM1* and *GSTT1* polymorphism because we cannot calculate the HWE on the both genes. Third, no data were extracted on other risk factors, such as hormonal readiness, obesity, smoking, and so on.

## Conclusions

In summary, this meta-analysis indicates that individual and combined effects of *GSTM1*, *GSTT1* and *GSTP1* polymorphisms may be not associated with increased BC risk.

## Supporting information

S1 Table Scale for quality assessment of molecular association studies of breast cancer. (PDF)

S2 Table General characteristics of studies included in pooling gene effects. (PDF)

S3 Table Quality assessment by included studies of *GSTM1* and *GSTT1* polymorphisms with breast cancer risk. (PDF)

S4 Table Quality assessment by included studies of *GSTP1* polymorphisms with breast cancer risk. (PDF)

S5 Table Genotype frequencies of the *GSTM1*, *GSTT1*, and *GSTP1* IIe105Val polymorphisms between breast cancer and control groups. (PDF)

S6 Table Genotype frequencies of the *GSTM1*, *GSTT1*, and *GSTP1* IIe105Val polymorphisms between postmenopausal and premenopausal breast cancer and control groups. (PDF)

S7 Table Genotype frequencies of the *GSTM1*, *GSTT1*, and *GSTP1* IIe105Val polymorphisms between and breast cancer and control groups by smoking status. (PDF)

S8 Table Genotype frequencies of the combined effects of *GSTM1* present/null and *GSTT1* present/null between breast cancer and control groups. (PDF)

S9 Table Genotype frequencies of the combined effects of GSTM1 and GSTT1 between postmenopausal and premenopausal breast cancer and control groups. (PDF)

S10 Table Genotype frequencies of the combined effects of *GSTM1* present/null and *GSTP1* IIe105Val between breast cancer and control groups. (PDF)

S11 Table Genotype frequencies of the combined effects of *GSTT1* present/null and *GSTP1* IIe105Val between breast cancer and control groups. (PDF)

S12 Table Genotype frequencies of the combined effects of *GSTM1* present/null, *GSTT1* present/null, and *GSTP1* IIe105Val between breast cancer and control groups. (PDF)

S1 Fig Begg’s funnel plot to assess publication bias on *GSTM1* polymorphism in overall population. (PDF)

S2 Fig Begg’s funnel plot to assess publication bias on *GSTT1* polymorphism in overall population. (PDF)

S3 Fig Begg’s funnel plot to assess publication bias on *GSTP1* polymorphism in overall population (Val/Val vs. IIe/IIe). (PDF)

S4 Fig Begg’s funnel plot to assess publication bias on *GSTP1* polymorphism in overall population (IIe/Val vs. IIe/IIe). (PDF)

S5 Fig Begg’s funnel plot to assess publication bias on *GSTP1* polymorphism in overall population (Val/Val vs. IIe/IIe +IIe/Val). (PDF)

S6 Fig Begg’s funnel plot to assess publication bias on *GSTP1* polymorphism in overall population (Val vs. IIe). (PDF)

S7 Fig Begg’s funnel plot to assess publication bias on GSTP1 polymorphism in overall population (Val/Val + IIe/Val vs. IIe/IIe). (PDF)

S8 Fig “trim and fill” plots for the publication bias evaluation between the combined effects of *GSTM1* and *GSTT1* polymorphisms and breast cancer risk (*− − vs*. (*+ +*)). (PDF)

S9 Fig “trim and fill” plots for the publication bias evaluation between the combined effects of *GSTM1* and *GSTT1* polymorphisms and breast cancer risk ((+ *−*) + (*−* +) vs. (+ +)). (PDF)

S10 Fig “trim and fill” plots for the publication bias evaluation between the combined effects of *GSTM1* and *GSTT1* polymorphisms and breast cancer risk ((*−* +) + (+ *−*) + (*− −*) vs. + +). (PDF)

S11 Fig “trim and fill” plots for the publication bias evaluation between the combined effects of *GSTM1* and *GSTT1* polymorphisms and breast cancer risk (*− −* vs. (*−* +) + (+ *−*) + (+ +)). (PDF)

S12 Fig “trim and fill” plots for the publication bias evaluation between the combined effects of *GSTM1* and *GSTP1* polymorphisms and breast cancer risk (*GSTM1* null/*GSTP1* Val* vs. *GSTM1* present/GSTP1 IIe/IIe). (PDF)

S13 Fig “trim and fill” plots for the publication bias evaluation between the combined effects of *GSTM1* and *GSTP1* polymorphisms and breast cancer risk (All risk genotypes vs. *GSTM1* present/*GSTP1* IIe/IIe). (PDF)

S14 Fig “trim and fill” plots for the publication bias evaluation between the combined effects of *GSTT1* and *GSTP1* polymorphisms and breast cancer risk (*GSTT1* null/*GSTP1* val* vs. *GSTT1* present/*GSTP1* IIe/IIe). (PDF)

S15 Fig “trim and fill” plots for the publication bias evaluation between the combined effects of *GSTT1* and *GSTP1* polymorphisms and breast cancer risk (All risk genotypes vs. *GSTT1* present/*GSTP1* IIe/IIe). (PDF)

S16 Fig “trim and fill” plots for the publication bias evaluation between the combined effects of *GSTT1* and *GSTP1* polymorphisms and breast cancer risk (*GSTT1* null/*GSTP1* Val* vs. *GSTT1* null/*GSTP1* IIe/IIe + *GSTT1* present/*GSTP1* Val* + *GSTT1* present/*GSTP1* IIe/IIe). (PDF)

S17 Fig “trim and fill” plots for the publication bias evaluation between the combined effects of *GSTT1*, *GSTM1* and *GSTP1* polymorphisms and breast cancer risk (All two high-risk genotype vs. M1 present/T1 present/P1 IIe/IIe). (PDF)

S18 Fig “trim and fill” plots for the publication bias evaluation between the combined effects of GSTT1, GSTM1 and GSTP1 polymorphisms and breast cancer risk (M1 null/T1 null/P1 Val * vs.M1 present/T1 present/P1 IIe/IIe). (PDF)

S19 Fig “trim and fill” plots for the publication bias evaluation between the combined effects of *GSTT1*, *GSTM1* and *GSTP1* polymorphisms and breast cancer risk (M1 null/T1 null/P1 Val* vs. M1 present/T1 present/P1 IIe/IIe + all one high risk + all two high risk). (PDF)

S1 Appendix References. (PDF)

S2 Appendix Prototype Excel spreadsheet showing input and output for false positive report probability (FPRP) calculations. (XLS)

S1 File Prisma checklist. (DOC)

S2 File Meta analysis on genetic association studies form. (DOCX)
