# Supplementary material for: Individual and combined effects of GSTM1, GSTT1, and GSTP1 polymorphisms on breast cancer risk: A meta-analysis and re-analysis of systematic meta-analyses
Source: PLoS One. 2020 Mar 10;15(3):e0216147. doi: 10.1371/journal.pone.0216147 (PMC7064184; doi:10.1371/journal.pone.0216147)
Supplement: S1 Table — (PDF) [file pone.0216147.s001.pdf]

Criterion	Score
Source of case	
Selected from population or cancer registry	3
Selected from hospital	2
Selected from pathology archives, but without description	1
Not described	0
Source of control	
Population-based	3
Blood donors or volunteers	2
Hospital-based	1
Not described	0
Ascertainment of cancer	
Histological or pathological confirmation	2
Diagnosis of lung cancer by patient medical record	1
Not described	0
Ascertainment of control	
Controls were tested to screen out breast cancer	2
Controls were subjects who did not report breast cancer, no objective testing	1
Not described	0
Matching	
Controls matched with cases only by age	1
Not matched or not described	0
Genotyping examination	
Genotyping done blindly and quality control	2
Only genotyping done blindly or quality control	1
Unblinded and without quality control	0
Specimens used for determining genotypes	
Blood cells or normal tissues	1
Tumor tissues or exfoliated cells of tissue	0
Hardy-Weinberg equilibrium	
Hardy-Weinberg equilibrium in the control group	1
Hardy-Weinberg disequilibrium in the control group	0
Total sample size	
>1000	3
500-1000	2
200-500	1
<200	0
