# Supplementary material for: Individual and combined effects of GSTM1, GSTT1, and GSTP1 polymorphisms on breast cancer risk: A meta-analysis and re-analysis of systematic meta-analyses
Source: PLoS One. 2020 Mar 10;15(3):e0216147. doi: 10.1371/journal.pone.0216147 (PMC7064184; doi:10.1371/journal.pone.0216147)
Supplement: S2 Table — (PDF) [file pone.0216147.s002.pdf]

First author/Year	Country	Race	SC	Control source	Type of control	Matching	Material used for assessment of genotype	SNP
Zhong [1] 1993	UK	Caucasian	HB	HB and Volunteers	ND	ND	Blood	*GSTM1*
Kelsey [3] 1997	USA	Mixed	PB	PB	Cancer-free women	Age	Blood	*GSTM1*
Harries [110] 1997	UK	Caucasian	HB	HB	Cancer-free women	ND	Whole blood	*GSTP1*
Helzlsouer [5] 1998	USA	Mixed	PB	PB	ND	Age	Blood	*GSTM1*, *GSTT1*, and *GSTP1*
Bailey [6] 1998	USA	African	HB	HB	Cancer-free women	Age	Blood	*GSTM1* and *GSTT1*
Bailey [6] 1998	USA	Caucasian	HB	HB	Cancer-free women	Age	Blood	*GSTM1* and *GSTT1*
García-Closas [7] 1999	USA	Mixed	PB	PB	Cancer-free women	Age	Blood	*GSTM1* and *GSTT1*
Ambrosone [8] 1999	USA	Caucasian	HB	PB	Healthy women	Age	Blood	*GSTM1*
Charrier [9] 1999	France	Caucasian	PB	PB	Cancer-free women	ND	Blood	*GSTM1*
Curran [11] 2000	Australia	Caucasian	HB	Volunteers	Cancer-free women	Age	Blood	*GSTM1*, *GSTT1*, and *GSTP1*
Millikan [12] 2000	USA	African	PB	PB	ND	Age	Peripheral blood	*GSTM1*, *GSTT1*, and *GSTP1*
Millikan [12] 2000	USA	Caucasian	PB	PB	ND	Age	Peripheral blood	*GSTM1*, *GSTT1*, and *GSTP1*
Rundle [13] 2000	USA	Mixed	HB	HB	Benign breast disease	ND	Blood leukocyte	*GSTM1*
Xiong [14] 2001	USA	Caucasian	HB	HB	Cancer-free women	Age	Blood	*GSTM1* and *GSTT1*
Gudmundsdottir [15] 2001	Iceland	Caucasian	ND	ND	Healthy women	ND	Blood and tumor tissue (case), blood (control)	*GSTM1*, *GSTT1*, and *GSTP1*
Dialyna [16] 2001	Greece	Caucasian	HB	HB	Healthy women	Age	Blood and tumor (case), blood (control)	*GSTM1* and *GSTT1*
Mitrunen [17] 2001	Finland	Caucasian	HB	PB	Health women	ND	Blood	*GSTM1*, *GSTT1*, and *GSTP1*
Krajinovic [18] 2001	Canada	Caucasian	HB	HB	Healthy women	ND	Blood	*GSTM1*, *GSTT1*, and *GSTP1*
Maugard [19] 2001	France	Caucasian	HB	Blood donors and HB	Cancer-free women	Age	Blood	*GSTM1* and *GSTP1*
Zhao [20] 2001	USA	Caucasian	PB	PB	Cancer-free women	ND	Blood	*GSTP1*
Matheson [22] 2002	Australia	Caucasian	CR	NR	Cancer-free women	Age	Blood	*GSTM1* and *GSTT1*
Zheng T [23] 2002	USA	Mixed	HB	HB	Cancer-free women	Age	Blood	*GSTM1* and *GSTT1*
da Fonte de Amorim [24] 2002	Brazil	Caucasian	HB	HB	Out-patients	Age	Blood	*GSTM1* and *GSTT1*
da Fonte de Amorim [24] 2002	Brazil	Mixed	HB	HB	Out-patients	Age	Blood	*GSTM1* and *GSTT1*
Zheng W [25] 2002	USA	Caucasian	PB	PB	Cancer-free women	ND	Blood	*GSTM1* and *GSTT1*
Wu [26] 2002	China	Asian	HB	NR	Cancer-free women	Age	Blood	*GSTM1* and *GSTT1*
Siegelmann-Danieli [27] 2002	USA	Caucasian	HB	HB	Healthy women	ND	Blood	*GSTM1* and *GSTT1*
Li [28] 2002	USA	Mixed	HB	HB	Cancer-free women	ND	Normal breast tissues	*GSTM1*
Wang X F [104] 2002	China	Asian	HB	HB	Healthy women	ND	Blood	*GSTM1*
Khedhaier [30] 2003	Tunisia	African	HB	Blood donors	Healthy populations	ND	Peripheral blood leucocytes	*GSTM1* and *GSTT1*
Zhu [34] 2003	USA	Mixed	HB	HB	Cancer-free patients	ND	Normal breast tissue	*GSTM1*
Roodi [36] 2004	USA	Caucasian	HB	HB	Cancer-free women	Age	Blood	*GSTM1*
McCready [37] 2004	USA	Caucasian	HB	HB	Cancer-free patients	Age	Blood	*GSTM1* and *GSTT1*

Sarmanová [38] 2004	Czech Republic	Caucasian	HB	HB	Healthy women	ND	Blood	*GSTM1*, *GSTT1*, and *GSTP1*
Gago-Dominguez [39] 2004	Singapore	Asian	CR	PB	Cancer-free women	Age	Blood	*GSTM1*, *GSTT1*, and *GSTP1*
Egan [40] 2004	China	Asian	PB	PB	Cancer-free women	Age	Blood	*GSTM1*, *GSTT1*, and *GSTP1*
Park [41] 2004	Korea	Asian	HB	HB	Cancer-free women	Age	Blood	*GSTM1* and *GSTT1*
Kim [42] 2004	Korea	Asian	HB	HB	Cancer-free women	Age	Blood	*GSTP1*
Vogl [43] 2004	Multiple	Mixed	HB	HB	ND	ND	Blood	*GSTM1*
Medeiros [44] 2004	Portugal	Caucasian	ND	ND	Cancer-free women	ND	Blood	*GSTM1*
Linhares [46] 2005	Brazil	Caucasian	HB	HB	Cancer-free women	ND	Blood	*GSTM1*
Linhares [46] 2005	Brazil	African	HB	HB	Cancer-free women	ND	Blood	*GSTM1*
van der Hel [47] 2005	The Netherlands	Caucasian	PB	PB	Cancer-free women	Age	Blood	*GSTM1* and *GSTT1*
Ceschi [48] 2005	Singapore	Asian	PB	PB	Cancer-free women	ND	Peripheral blood and buccal cell	*GSTM1*, *GSTT1*, and *GSTP1*
Chacko [49] 2005	India	Indian	HB	HB	Cancer-free women	Age	Blood	*GSTM1* and *GSTT1*
Cheng [50] 2005	China	Asian	HB	HB	Healthy women	ND	Blood	*GSTM1* and *GSTT1*
Wu [51] 2006	China	Asian	HB	HB	Cancer-free women	ND	Blood	*GSTM1*
Chang [52] 2006	China	Asian	HB	HB	Healthy women	Age	Peripheral blood	*GSTM1*, *GSTT1*, and *GSTP1*
Onay [53] 2006	Canada	Caucasian	PB	PB	Cancer-free women	Age	Blood	*GSTP1*
Steck [55] 2007	USA	Mixed	HB	PB	Cancer-free women	Age	Blood	*GSTM1*, *GSTT1*, and *GSTP1*
Spurdle [56] 2007	USA	Caucasian	CR	PB	Cancer-free women	Age	Blood	*GSTM1*, *GSTT1*, and *GSTP1*
Edvardsen [57] 2007	Norway	Caucasian	HB	ND	Cancer-free women	ND	Blood	*GSTM1*, *GSTT1*, and *GSTP1*
Nordgard [58] 2007	Norway	Caucasian	HB	ND	Healthy women	ND	Peripheral venous blood	*GSTM1*, *GSTT1*, and *GSTP1*
Li SF [109] 2007	China	Asian	HB	HB	Cancer-free women	Age	Peripheral venous blood	*GSTM1* and *GSTT1*
Justenhoven [59] 2008	Germany	Caucasian	PB	PB	Cancer-free women	Age	Blood	*GSTP1*
Torresan [60] 2008	Brazil	Mixed	HB	PB	Healthy women	Age	Peripheral venous blood	*GSTM1*, *GSTT1*, and *GSTP1*
Kadouri [61] 2008	Israel	Caucasian	HB	HB	Cancer-free women	ND	Blood	*GSTM1*, *GSTT1*, and *GSTP1*
Van Emburgh [62] 2008	USA	Caucasian	HB	HB	Cancer-free women	Age	Blood	*GSTM1*, *GSTT1*, and *GSTP1*
Van Emburgh [62] 2008	USA	African	HB	HB	Cancer-free women	Age	Blood	*GSTM1*, *GSTT1*, and *GSTP1*
Syamala [63] 2008	India	Indian	HB	HB	Outpatients	ND	Blood	*GSTM1*, *GSTT1*, and *GSTP1*
Rajkumar [64] 2008	India	Indian	ND	ND	Healthy women	Age	Blood	*GSTM1*, *GSTT1*, and *GSTP1*
Sakoda [65] 2008	China	Asian	PB	PB	Healthy women	Age	Blood	*GSTM1* and *GSTP1*
Lee [66] 2008	China	Asian	PB	PB	Healthy women	ND	Blood	*GSTP1*
Unlu [67] 2008	Turkey	Caucasian	HB	ND	Healthy women	ND	Blood	*GSTM1*, *GSTT1*, and *GSTP1*
Li JY [97] 2008	China	Asian	HB	HB	Cancer-free women	ND	Blood	*GSTM1* and *GSTT1*
Morais [113] 2008	Portugal	Caucasian	HB	HB	Cancer-free women	ND	Blood	*GSTM1* and *GSTT1*
Chang YL [114] 2008	China	Asian	HB	HB	Cancer-free patients	ND	Blood	*GSTT1*

Kostrykina [68] 2009	Russia	Caucasian	HB	ND	Cancer-free women	ND	Blood	*GSTM1* and *GSTT1*
McCarty [69] 2009	USA	Mixed	PB	PB	Cancer-free women	Age	Blood	*GSTM1, GSTT1* and *GSTP1*
Reding [70] 2009	USA	Mixed	PB	PB	Cancer-free women	Age	Blood	*GSTM1*, *GSTT1*, and *GSTP1*
Yu [71] 2009	China	Asian	HB	HB	Cancer-free women	Age	Blood	*GSTM1*
Saxena [72] 2009	India	Indian	HB	PB	Cancer-free women	ND	Blood	*GSTM1*, *GSTT1*, and *GSTP1*
Antognelli [73] 2009	Italy	Caucasian	HB	PB	Healthy women	Age	Heparinized peripheral whole blood	*GSTP1*
Pongtheerat [74] 2009	Thailand	Asian	HB	ND	Healthy women	ND	Breast cancer tissues (case)	*GSTM1*, *GSTT1*, and *GSTP1*
Kaushal [75] 2010	India	Indian	HB	HB	Cancer-free women	ND	Blood	*GSTM1*, *GSTT1*, and *GSTP1*
Masoudi [77] 2010	Iran	Caucasian	HB	HB	Healthy women	ND	Blood	*GSTM1*
MARIE-GENICA [78] 2010	German	Caucasian	PB	PB	Cancer-free women	Age	Blood	*GSTM1*, *GSTT1*, and *GSTP1*
Delort [79] 2010	France	Caucasian	HB	PB	Healthy women	ND	Whole blood	*GSTP1*
Sangrajrang [80] 2010	Thailand	Asian	HB	HB	Healthy women	ND	Blood	*GSTP1*
Geng Y [29] 2010	China	Asian	HB	ND	Healthy women	ND	Normal tissues (case)	*GSTP1*
Cui Z [105] 2010	China	Asian	HB	PB	Healthy women	ND	Blood	*GSTM1* and *GSTT1*
Li J [108] 2010	China	Asian	HB	HB	Cancer-free patients	ND	Blood	*GSTM1*
Ermolenko [112] 2010	Russian	Caucasian	HB	HB	Cancer-free women	ND	Blood	*GSTP1*
Nosheen [81] 2011	Pakistan	Middle East	HB	HB	Healthy women	Age	Blood	*GSTM1* and *GSTT1*
Cribb [83] 2011	Canada	Caucasian	HB	PB	Cancer-free women	Age	Blood	*GSTM1*
Naushad [84] 2011	India	Indian	HB	HB	Healthy women	ND	Blood	*GSTM1* and *GSTT1*
Cerne [85] 2011	Slovenia	Caucasian	HB	HB	Outpatient	Age	Normal breast tissues (case)	*GSTP1*
Reding [86] 2012	USA	Caucasian	PB	PB	Cancer-free women	Age	Blood	*GSTM1*, *GSTT1*, and *GSTP1*
Reding [86] 2012	USA	African	PB	PB	Cancer-free women	Age	Blood	*GSTM1*, *GSTT1*, and *GSTP1*
Hashemi [87] 2012	Iran	Caucasian	HB	PB	Health women	ND	Blood	*GSTM1*, *GSTT1*, and *GSTP1*
Ramalhinho [88] 2012	Portugal	Caucasian	HB	Blood donors	Health women	ND	Blood	*GSTM1*, *GSTT1*, and *GSTP1*
Luo [89] 2012	China	Asian	PB	PB	Cancer-free women	Age	Blood or exfoliated buccal cell	*GSTM1* and *GSTT1*
Saxena [90] 2012	India	Indian	HB	Blood donors	Health women	ND	breast cancer tissue (case)	*GSTP1*
Fan B [106] 2012	China	Asian	HB	HB	Cancer-free patients	ND	Blood	*GSTM1* and *GSTT1*
Sohail [92] 2013	Pakistan	Middle East	HB	Volunteers	Health women	Age	Venous blood	*GSTM1*, *GSTT1*, and *GSTP1*
Zgheib [93] 2013	Lebanon	Asian	HB	HB	Cancer-free women	ND	Blood	*GSTM1*, *GSTT1*, and *GSTP1*
Possuelo [94] 2013	Brazil	Mixed	HB	HB	Health women	Age	Peripheral blood	*GSTM1* and *GSTT1*
Ge [95] 2013	China	Asian	HB	ND	Health women	ND	Peripheral blood	*GSTP1*
Chirilă [96] 2014	Romania	Caucasian	HB	ND	Health women	ND	Blood	*GSTM1*, *GSTT1*, and *GSTP1*
Khabaz [98] 2014	Jordan	ME	HB	HB	Cancer-free women	ND	Blood	*GSTP1*
Khabaz [99] 2015	Saudi Arabia	ME	HB	HB	Cancer-free women	ND	Blood	*GSTP1*

Soto-Quintana [100] 2015	México	Mixed	HB	Volunteers	Health women	ND	Peripheral blood	*GSTM1*
Jaramillo-Rangel [101] 2015	México	Mixed	HB	HB	Cancer-free women	ND	Peripheral blood	*GSTM1*, *GSTT1*, and *GSTP1*
Kimi [102] 2016	India	Indian	HB	Healthy volunteers	Health women	Age	Whole blood	*GSTM1*, *GSTT1*, and *GSTP1*
Kong Z [116] 2016	China	Asian	HB	HB	Cancer-free women	ND	Peripheral blood	*GSTP1*
García-Martínez [103] 2017	México	Mixed	HB	PB	Cancer-free women	Age	Blood	*GSTM1* and *GSTT1*

HB: hospital-based study, PB: population-based study, CR: cancer registry, ND: not described
