# Supplementary material for: Individual and combined effects of GSTM1, GSTT1, and GSTP1 polymorphisms on breast cancer risk: A meta-analysis and re-analysis of systematic meta-analyses
Source: PLoS One. 2020 Mar 10;15(3):e0216147. doi: 10.1371/journal.pone.0216147 (PMC7064184; doi:10.1371/journal.pone.0216147)
Supplement: S3 Table — (PDF) [file pone.0216147.s003.pdf]

First author/Year	Source of case	Source of control	Ascertainment of cancer	Ascertainment of control	Matching	Genotyping examination	Specimens used for determining genotypes	Sample size	Quality score
Zhong [1] 1993	2	1.5	0	0	0	0	1	1	5.5
Kelsey [3] 1997	3	3	0	2	1	2	1	1	13
Helzlsouer [5] 1998	3	3	2	0	1	1	1	1	12
Bailey [6] 1998	2	1	2	1	1	1	1	0	9
Bailey [6] 1998	2	1	2	1	1	1	1	1	10
García-Closas [7] 1999	3	3	1	1	1	2	1	2	14
Ambrosone [8] 1999	2	3	2	0	1	1	1	2	12
Charrier [9] 1999	3	3	2	1	0	0	1	2	12
Curran [11] 2000	2	2	2	1	1	0	1	1	10
Millikan [12] 2000	3	3	2	1	1	1	1	2	14
Millikan [12] 2000	3	3	2	1	1	1	1	2	14
Rundle [13] 2000	2	1	2	1	0	0	1	0	7
Xiong [14] 2001	2	1	2	1	1	0	1	1	9
Gudmundsdottir [15] 2001	0	0	0	1	0	0	0	2	3
Dialyna [16] 2001	2	1	1	1	1	0	0	1	7
Mitrunen [17] 2001	2	3	2	1	0	1	1	2	12
Krajinovic [18] 2001	2	1	2	1	0	0	1	1	8
Maugard [19] 2001	2	1.5	2	1	1	0	1	1	9.5
Matheson [22] 2002	3	0	0	1	1	0	1	1	7
Zheng T [23] 2002	2	1	2	2	1	2	1	2	13
da Fonte de Amorim [24] 2002	2	1	0	1	1	0	1	1	7
da Fonte de Amorim [24] 2002	2	1	0	1	1	0	1	0	6
Zheng W [25] 2002	3	3	0	1	1	0	1	2	11
Wu [26] 2002	2	0	2	1	1	0	1	0	7
Siegelmann-Danieli [27] 2002	2	1	2	2	0	1	1	2	11
Li [28] 2002	2	1	0	1	0	2	1	0	7
Wang XF [104] 2002	2	1	0	1	0	0	1	0	5
Khedhaier [30] 2003	2	2	2	1	0	0	1	2	10
Zhu [34] 2003	2	1	0	1	0	1	1	0	6
Roodi [36] 2004	2	1	2	1	1	1	1	1	10
McCready [37] 2004	2	1	1	2	1	0	1	0	8

Sarmanová [38] 2004	2	1	2	1	0	0	1	2	9
Gago-Dominguez [39] 2004	3	3	2	1	1	1	1	2	14
Egan [40] 2004	3	3	2	1	1	1	1	3	15
Park [41] 2004	2	1	2	1	1	1	1	2	11
Vogl [43] 2004	2	2	2	1	0	0	1	3	11
Medeiros [44] 2004	0	0	2	1	0	0	1	0	4
Linhares [46] 2005	2	1	2	2	0	0	1	1	9
Linhares [46] 2005	2	1	2	2	0	0	1	0	8
van der Hel [47] 2005	3	3	1	1	1	1	1	3	14
Ceschi [48] 2005	3	3	2	1	0	1	1	2	13
Chacko [49] 2005	2	1	2	2	1	0	1	1	10
Cheng [50] 2005	2	1	2	2	0	1	1	3	12
Wu [51] 2006	2	1	2	2	0	0	1	1	9
Chang [52] 2006	2	1	2	2	1	0	1	2	11
Steck [55] 2007	2	3	2	1	1	1	1	3	14
Spurdle [56] 2007	3	3	1	1	1	1	1	3	14
Edvardsen [57] 2007	2	0	2	1	0	1	1	2	9
Nordgard [58] 2007	2	0	0	2	0	0	1	1	6
Li SF [109] 2007	2	1	2	1	1	0	1	1	9
Torresan [60] 2008	2	3	0	1	1	0	1	1	9
Kadouri [61] 2008	2	1	0	1	0	0	1	1	6
Van Emburgh [62] 2008	2	1	2	1	1	0	1	2	10
Van Emburgh [62] 2008	2	1	2	1	1	0	1	0	8
Syamala [63] 2008	2	1	2	1	0	1	1	2	10
Rajkumar [64] 2008	0	0	2	1	1	0	1	2	7
Sakoda [65] 2008	3	3	2	2	1	1	0	3	15
Unlu [67] 2008	2	0	2	0	0	0	1	0	5
Li JY [97] 2008	2	1	2	1	0	0	1	0	7
Morais [113] 2008	2	1	2	1	0	0	1	1	8
Chang YL [114] 2008	2	1	2	1	0	0	1	0	7
Kostrykina [68] 2009	2	0	2	1	0	0	1	1	7
McCarty [69] 2009	3	3	2	1	1	0	1	3	14
Reding [70] 2009	3	3	2	1	1	1	1	3	15
Yu [71] 2009	2	1	2	2	1	0	1	3	12

Saxena [72] 2009	2	3	1	0	0	1	1	2	10
Pongtheerat [74] 2009	2	0	0	1	0	0	0	0	3
Kaushal [75] 2010	2	1	2	1	0	0	1	1	8
Masoudi [77] 2010	2	1	2	1	0	1	1	1	9
MARIE-GENICA [78] 2010	3	3	2	1	1	1	1	3	15
Cui Z [105] 2010	2	1	2	1	0	0	1	2	9
Li J [108] 2010	2	1	2	1	0	0	1	0	7
Nosheen [81] 2011	2	1	2	1	1	0	1	1	9
Cribb [83] 2011	2	3	2	2	1	1	1	2	14
Naushad [84] 2011	2	1	2	1	0	0	1	2	9
Reding [86] 2012	3	3	2	1	1	1	1	3	15
Reding [86] 2012	3	3	2	1	1	1	1	2	14
Hashemi [87] 2012	2	3	2	1	0	0	1	1	10
Ramalhinho [88] 2012	2	2	2	1	0	1	1	1	10
Luo [89] 2012	3	3	2	1	1	1	1	3	15
Fan B [106] 2012	2	1	2	1	0	0	1	0	7
Sohail [92] 2013	2	2	2	1	1	0	1	1	10
Zgheib [93] 2013	2	1	0	1	0	1	1	1	7
Possuelo [94] 2013	2	1	2	1	1	0	1	0	8
Chirilă [96] 2014	2	0	0	0	0	0	1	0	3
Soto-Quintana [100] 2015	2	2	2	1	0	0	1	2	10
Jaramillo-Rangel [101] 2015	2	1	2	1	0	0	1	1	8
Kimi [102] 2016	2	2	2	1	1	0	1	0	9
García-Martínez [103] 2017	2	3	2	1	1	1	1	3	14
