# Supplementary material for: Individual and combined effects of GSTM1, GSTT1, and GSTP1 polymorphisms on breast cancer risk: A meta-analysis and re-analysis of systematic meta-analyses
Source: PLoS One. 2020 Mar 10;15(3):e0216147. doi: 10.1371/journal.pone.0216147 (PMC7064184; doi:10.1371/journal.pone.0216147)
Supplement: S4 Table — (PDF) [file pone.0216147.s004.pdf]

First author/Year	Source of case	Source of control	Ascertainment of cancer	Ascertainment of control	Matching	Genotyping examination	Specimens used for determining genotypes	Sample size	HWE	Quality score
Harries [110] 1997	2	1	1	1	0	0	1	1	1	8
Helzlsouer [5] 1998	3	3	2	0	1	1	1	1	1	13
Curran [11] 2000	2	2	2	1	1	0	1	1	0	10
Millikan [12] 2000	3	3	2	1	1	1	1	2	1	15
Millikan [12] 2000	3	3	2	1	1	1	1	2	1	15
Gudmundsdottir [15] 2001	0	0	0	1	0	0	0	2	1	4
Mitrunen [17] 2001	2	3	2	1	0	1	1	2	1	13
Krajinovic [18] 2001	2	1	2	1	0	0	1	1	1	9
Maugard [19] 2001	2	1.5	2	1	1	0	1	1	1	10.5
Zhao [20] 2001	3	3	0	1	0	0	1	1	1	10
Sarmanová [38] 2004	2	1	2	1	0	0	1	2	1	10
Gago-Dominguez [39] 2004	3	3	2	1	1	1	1	2	0	14
Kim [42] 2004	2	1	2	1	1	1	1	1	1	11
Ceschi [48] 2005	3	3	2	1	0	1	1	2	1	14
Chang [52] 2006	2	1	2	2	1	0	1	2	0	11
Onay [53] 2006	3	3	2	1	1	1	1	2	1	15
Steck [55] 2007	2	3	2	1	1	1	1	3	1	15
Spurdle [56] 2007	3	3	1	1	1	1	1	3	1	15
Edvardsen [57] 2007	2	0	2	1	0	1	1	2	1	10
Nordgard [58] 2007	2	0	0	2	0	0	1	1	1	7
Justenhoven [59] 2008	3	3	1	1	1	1	1	3	1	15
Torresan [60] 2008	2	3	0	1	1	0	1	1	1	10
Kadouri [61] 2008	2	1	0	1	0	0	1	1	1	7
Van Emburgh [62] 2008	2	1	2	1	1	0	1	2	1	11
Van Emburgh [62] 2008	2	1	2	1	1	0	1	0	1	9
Syamala [63] 2008	2	1	2	1	0	1	1	2	1	11
Rajkumar [64] 2008	0	0	2	1	1	0	1	2	1	8
Sakoda [65] 2008	3	3	2	2	1	1	0	3	1	16
Lee [66] 2008	3	3	2	1	0	0	1	3	0	13
Unlu [67] 2008	2	0	2	0	0	0	1	0	0	5
McCarty [69] 2009	3	3	2	1	1	0	1	3	1	15

Reding [70] 2009	3	3	2	1	1	1	1	3	1	16
Saxena [72] 2009	2	3	1	0	0	1	1	2	1	11
Antognelli [73] 2009	2	3	2	1	1	1	1	3	0	14
Pongtheerat [74] 2009	2	0	0	1	0	0	0	0	0	3
Kaushal [75] 2010	2	1	2	1	0	0	1	1	1	9
MARIE-GENICA [78] 2010	3	3	2	1	1	1	1	3	0	15
Delort [79] 2010	2	3	2	1	0	1	1	3	0	13
Sangrajrang [80] 2010	2	1	2	2	1	1	1	3	1	14
Geng Y [29] 2010	2	0	2	1	0	0	1	0	0	6
Ermolenko [112] 2010	2	1	2	1	0	0	1	3	1	11
Cerne [85] 2011	2	1	0	1	1	1	1	2	0	9
Reding [86] 2012	3	3	2	1	1	1	1	3	1	16
Reding [86] 2012	3	3	2	1	1	1	1	2	1	15
Hashemi [87] 2012	2	3	2	1	0	0	1	1	1	11
Ramalhinho [88] 2012	2	2	2	1	0	1	1	1	0	10
Saxena [90] 2012	2	2	2	1	0	1	0	1	0	9
Sohail [92] 2013	2	2	2	1	1	0	1	1	1	11
Zgheib [93] 2013	2	1	0	1	0	1	1	1	0	7
Ge [95] 2013	2	0	2	1	0	1	1	3	1	11
Chirilă [96] 2014	2	0	0	0	0	0	1	0	1	4
Khabaz [98] 2014	2	1	2	1	0	0	1	0	1	8
Khabaz [99] 2015	2	1	2	1	0	0	1	0	1	8
Jaramillo-Rangel [101] 2015	2	1	2	1	0	0	1	1	1	9
Kimi [102] 2016	2	2	2	1	1	0	1	0	0	9
Kong Z [116] 2016	2	1	2	2	0	1	1	1	1	11

HWE: Hardy-Weinberg equilibrium
