# Supplementary material for: Individual and combined effects of GSTM1, GSTT1, and GSTP1 polymorphisms on breast cancer risk: A meta-analysis and re-analysis of systematic meta-analyses
Source: PLoS One. 2020 Mar 10;15(3):e0216147. doi: 10.1371/journal.pone.0216147 (PMC7064184; doi:10.1371/journal.pone.0216147)
Supplement: S5 Table — (PDF) [file pone.0216147.s005.pdf]

First author/Year	Sample size	*GSTM1*genotype distribution				*GSTT1* genotype distribution				*GSTP1* IIe105Val genotype distribution						HWE for *GSTP1*
		Case		Control		Case		Control		Case			Control			
		present	null	present	null	present	null	present	null	IIe/IIe	IIe/Val	Val/Val	IIe/IIe	IIe/Val	Val/Val	
Zhong [1] 1993	197/225	103	94	131	94	NA	NA	NA	NA	NA	NA	NA	NA	NA	NA	NA
Kelsey [3] 1997	484/484	224	260	243	241	NA	NA	NA	NA	NA	NA	NA	NA	NA	NA	NA
Harries [110] 1997	62/234	NA	NA	NA	NA	NA	NA	NA	NA	25	32	5	113	101	20	0.700
Helzlsouer [5] 1998	115/115	39	71	60	52	80	30	88	24	41	54	15	56	48	9	0.772
Bailey [6] 1998	59/59	39	20	35	24	47	12	42	17	NA	NA	NA	NA	NA	NA	NA
Bailey [6] 1998	164/162	73	91	62	100	117	47	118	44	NA	NA	NA	NA	NA	NA	NA
García-Closas [7] 1999	466/466	233	232	237	227	396	70	386	80	NA	NA	NA	NA	NA	NA	NA
Ambrosone [8] 1999	282/339	137	145	167	172	NA	NA	NA	NA	NA	NA	NA	NA	NA	NA	NA
Charrier [9] 1999	361/437	160	201	213	224	NA	NA	NA	NA	NA	NA	NA	NA	NA	NA	NA
Curran [11] 2000	129/129	56	73	57	72	101	27	108	20	63	55	11	59	64	6	0.028
Millikan [12] 2000	278/271	197	66	187	72	210	52	216	43	61	131	56	54	135	58	0.142
Millikan [12] 2000	410/392	189	194	177	192	331	58	312	61	178	155	35	141	169	38	0.229
Rundle [13] 2000	95/87	43	40	41	43	NA	NA	NA	NA	NA	NA	NA	NA	NA	NA	NA
Xiong [14] 2001	100/105	52	48	57	48	84	16	86	19	NA	NA	NA	NA	NA	NA	NA
Gudmundsdottir [15] 2001	500/395	227	273	181	214	401	99	314	81	202	225	73	177	172	46	0.669
Dialyna [16] 2001	207/171	99	108	82	89	177	30	154	17	NA	NA	NA	NA	NA	NA	NA
Mitrunen [17] 2001	483/482	260	221	278	200	411	70	415	63	283	178	22	266	181	34	0.673
Krajinovic [18] 2001	149/207	67	80	98	109	114	34	161	28	69	55	5	91	73	13	0.753
Maugard [19] 2001	220/196	175	45	137	59	NA	NA	NA	NA	99	101	20	81	90	15	0.144
Zhao [20] 2001	156/332	NA	NA	NA	NA	NA	NA	NA	NA	87	58	10	170	133	29	0.682
Matheson [22] 2002	157/157	66	91	80	77	113	44	139	18	NA	NA	NA	NA	NA	NA	NA
Zheng T [23] 2002	338/345	152	165	160	173	223	95	259	74	NA	NA	NA	NA	NA	NA	NA
da Fonte de Amorim [24] 2002	79/123	46	33	58	65	64	15	92	31	NA	NA	NA	NA	NA	NA	NA
da Fonte de Amorim [24] 2002	49/133	32	17	95	38	32	17	99	34	NA	NA	NA	NA	NA	NA	NA
Zheng W [25] 2002	273/657	102	100	232	249	113	39	263	62	NA	NA	NA	NA	NA	NA	NA
Wu [26] 2002	60/60	26	34	35	25	33	27	34	26	NA	NA	NA	NA	NA	NA	NA
Siegelmann-Danieli [27] 2002	402/238	177	225	104	134	NA	NA	NA	NA	NA	NA	NA	NA	NA	NA	NA
Li [28] 2002	73/58	44	29	43	15	NA	NA	NA	NA	NA	NA	NA	NA	NA	NA	NA
Wang XF [104] 2002	42/108	18	24	56	52	NA	NA	NA	NA	NA	NA	NA	NA	NA	NA	NA
Khedhaier [30] 2003	309/242	143	166	104	138	206	103	179	63	NA	NA	NA	NA	NA	NA	NA

Zhu [34] 2003	102/46	58	44	37	9	NA	NA	NA	NA	NA	NA	NA	NA	NA	NA	NA
Roodi [36] 2004	203/202	86	117	77	125	NA	NA	NA	NA	NA	NA	NA	NA	NA	NA	NA
McCready [37] 2004	70/70	21	49	33	35	50	13	55	9	NA	NA	NA	NA	NA	NA	NA
Sarmanová [38] 2004	238/313	105	133	156	155	201	33	266	44	95	111	30	146	132	31	0.884
Gago-Dominguez [39] 2004	180/466	98	82	248	218	114	66	262	204	115	65		304	162		NA
Egan [40] 2004	1,144/1,221	497	638	523	683	579	557	614	596	NA	NA	NA	NA	NA	NA	NA
Park [41] 2004	202/299	84	116	137	152	99	101	168	121	NA	NA	NA	NA	NA	NA	NA
Kim [42] 2004	189/233	NA	NA	NA	NA	NA	NA	NA	NA	122	44	5	113	52	6	0.995
Vogl [43] 2004	130/122	79	51	58	64	NA	NA	NA	NA	NA	NA	NA	NA	NA	NA	NA
Medeiros [44] 2004	41/123	20	21	51	72	NA	NA	NA	NA	NA	NA	NA	NA	NA	NA	NA
van der Hel [45] 2004	229/263	96	133	134	129	193	36	213	50	NA	NA	NA	NA	NA	NA	NA
Linhares [46] 2005	85/196	61	24	122	74	NA	NA	NA	NA	NA	NA	NA	NA	NA	NA	NA
Linhares [46] 2005	20/82	12	8	52	30	NA	NA	NA	NA	NA	NA	NA	NA	NA	NA	NA
van der Hel [47] 2005	676/704	355	362	396	371	443	274	542	225	NA	NA	NA	NA	NA	NA	NA
Ceschi [48] 2005	258/670	137	119	369	298	169	87	385	282	161	87	9	442	199	27	0.443
Chacko [49] 2005	112/112	72	40	84	28	83	29	102	10	NA	NA	NA	NA	NA	NA	NA
Cheng [50] 2005	469/740	231	234	371	362	238	223	400	336	NA	NA	NA	NA	NA	NA	NA
Wu [51] 2006	262/225	139	123	122	103	NA	NA	NA	NA	NA	NA	NA	NA	NA	NA	NA
Chang [52] 2006	189/420	82	107	193	227	78	111	210	210	123	66		288	133		NA
Onay [53] 2006	398/372	NA	NA	NA	NA	NA	NA	NA	NA	208	149	41	175	161	36	0.907
Steck [55] 2007	1052/1098	547	505	604	494	831	221	856	242	536	421	95	528	472	88	0.219
Spurdle [56] 2007	1246/664	571	675	333	331	1,032	214	555	109	539	545	148	283	286	80	0.557
Edvardsen [57] 2007	272/272	152	120	151	118	228	44	224	45	119	123	30	105	118	45	0.232
Nordgard [58] 2007	93/109	39	52	55	53	76	16	90	18	44	41	7	50	46	12	0.773
Li SF [109] 2007	104/152	37	57	60	70	40	51	72	56	NA	NA	NA	NA	NA	NA	NA
Justenhoven [59] 2008	688/724	NA	NA	NA	NA	NA	NA	NA	NA	259	271	67	276	268	67	0.872
Torresan [60] 2008	102/102	46	56	46	56	71	31	69	33	54	35	13	77	22	3	0.367
Kadouri [61] 2008	211/109	106	105	46	63	158	53	84	24	121	74	16	76	29	3	0.907
Van Emburgh [62] 2008	391/466	185	206	198	268	322	69	384	82	160	183	42	179	179	35	0.301
Van Emburgh [62] 2008	56/87	37	19	59	28	41	15	71	16	14	29	13	25	39	13	0.737
Syamala [63] 2008	347/250	228	119	187	63	291	56	227	23	186	140	21	125	109	16	0.225
Rajkumar [64] 2008	250/500	185	65	390	110	206	44	416	84	118	103	29	230	219	51	0.915
Sakoda [65] 2008	615/879	294	321	450	428	NA	NA	NA	NA	378	215	20	569	277	30	0.600
Lee [66] 2005	3026/3037	NA	NA	NA	NA	NA	NA	NA	NA	1950	953	123	2003	949	85	0.029

Unlu [67] 2008	65/108	31	34	61	47	39	26	76	32	28	26	11	51	37	20	0.009
Li JY [97] 2008	78/78	47	31	41	37	43	35	34	44	NA	NA	NA	NA	NA	NA	NA
Morais [113] 2008	100/169	60	40	94	75	80	20	136	33	NA	NA	NA	NA	NA	NA	NA
Chang YL [114] 2008	70/70	NA	NA	NA	NA	32	38	40	30	NA	NA	NA	NA	NA	NA	NA
Kostrykina [68] 2009	695/263	376	328	140	123	563	133	213	50	NA	NA	NA	NA	NA	NA	NA
McCarty [69] 2009	975/1001	505	470	547	454	774	209	795	221	510	517		551	518		0.38
Reding [70] 2009	891/878	421	467	415	460	744	147	738	139	382	417	92	366	390	119	0.353
Yu [71] 2009	1017/903	395	622	393	510	NA	NA	NA	NA	NA	NA	NA	NA	NA	NA	NA
Saxena [72] 2009	413/410	192	215	269	134	310	96	315	88	147	193	66	200	171	32	0.586
Antognelli [73] 2009	547/544	NA	NA	NA	NA	NA	NA	NA	NA	315	215	15	128	340	76	<0.001
Pongtheerat [74] 2009	43/56	26	14	32	24	25	18	28	25	30	13		32	21		NA
Kaushal [75] 2010	117/174	94	23	122	52	84	33	105	69	62	48	7	108	62	4	0.152
Masoudi [77] 2010	181/181	90	91	70	111	NA	NA	NA	NA	NA	NA	NA	NA	NA	NA	NA
MARIE-GENICA [78] 2010	3,149/5,489	1,498	1,614	2,639	2,807	2,578	543	4,585	871	1,379	1,371	391	2,400	2,502	565	0.019
Delort [79] 2010	911/1000	NA	NA	NA	NA	NA	NA	NA	NA	853	52	5	932	59	5	0.0003
Sangrajrang [80] 2010	570/497	NA	NA	NA	NA	NA	NA	NA	NA	307	219	41	262	196	31	0.480
Geng Y [29] 2010	50/15	NA	NA	NA	NA	NA	NA	NA	NA	47	3		14	1		NA
Cui Z [105] 2010	315/360	159	156	270	90	168	147	282	78	NA	NA	NA	NA	NA	NA	NA
Li J [108] 2010	70/70	35	35	42	28	NA	NA	NA	NA	NA	NA	NA	NA	NA	NA	NA
Ermolenko [112] 2010	932/470	NA	NA	NA	NA	NA	NA	NA	NA	448	390	94	213	209	48	0.755
Nosheen [81] 2011	150/150	147	3	138	12	137	13	122	28	NA	NA	NA	NA	NA	NA	NA
Cribb [83] 2011	207/621	94	113	284	337	NA	NA	NA	NA	NA	NA	NA	NA	NA	NA	NA
Naushad [84] 2011	342/253	228	114	168	85	255	87	205	48	NA	NA	NA	NA	NA	NA	NA
Cerne [85] 2011	530/270	NA	NA	NA	NA	NA	NA	NA	NA	233	243	54	130	101	39	0.010
Reding [86] 2012	1151/995	509	609	477	501	927	205	805	177	478	498	154	416	439	124	0.626
Reding [86] 2012	493/456	343	127	320	122	366	114	335	113	147	241	84	124	230	91	0.406
Hashemi [87] 2012	134/152	48	86	81	71	116	18	140	12	36	72	26	97	52	3	0.183
Ramalhinho [88] 2012	101/121	35	66	76	45	54	47	97	24	54	47		57	64		NA
Luo [89] 2012	352/701	146	207	286	414	167	186	337	364	NA	NA	NA	NA	NA	NA	NA
Saxena [90] 2012	215/215	NA	NA	NA	NA	NA	NA	NA	NA	81	89	45	101	75	39	0.0005
Fan B [106] 2012	93/89	34	59	54	35	42	51	59	30	NA	NA	NA	NA	NA	NA	NA
Sohail [92] 2013	100/102	57	43	57	45	73	27	70	32	90	10	0	67	28	7	0.105
Zgheib [93] 2013	227/99	115	111	51	47	183	43	78	20	117	110		49	49		NA
Possuelo [94] 2013	49/49	20	29	31	18	39	10	42	7	NA	NA	NA	NA	NA	NA	NA

Ge [95] 2013	920/783	NA	NA	NA	NA	NA	NA	NA	NA	540	325	55	519	230	34	0.190
Chirilă [96] 2014	59/39	24	35	22	17	45	14	35	4	29	26	3	21	17	1	0.253
Khabaz [98] 2014	100/48	NA	NA	NA	NA	NA	NA	NA	NA	58	40	2	28	18	2	0.670
Khabaz [99] 2015	86/35	NA	NA	NA	NA	NA	NA	NA	NA	40	45	1	19	14	2	0.781
Soto-Quintana [100] 2015	558/276	306	252	172	104	NA	NA	NA	NA	NA	NA	NA	NA	NA	NA	NA
Jaramillo-Rangel [101] 2015	243/118	124	117	79	34	211	32	92	22	58	105	79	35	53	30	0.277
Kimi [102] 2016	22/10	2	20	4	6	8	14	6	4	15	5	2	10	0	0	NA
Kong Z [116] 2016	230/230	NA	NA	NA	NA	NA	NA	NA	NA	137	86	7	167	54	9	0.093
García-Martínez [103] 2017	952/998	472	455	533	422	682	245	662	293	NA	NA	NA	NA	NA	NA	NA

NA: not available; HWE: Hardy-Weinberg equilibrium
