# Supplementary material for: Individual and combined effects of GSTM1, GSTT1, and GSTP1 polymorphisms on breast cancer risk: A meta-analysis and re-analysis of systematic meta-analyses
Source: PLoS One. 2020 Mar 10;15(3):e0216147. doi: 10.1371/journal.pone.0216147 (PMC7064184; doi:10.1371/journal.pone.0216147)
Supplement: S6 Table — (PDF) [file pone.0216147.s006.pdf]

| **First author/Year** | **Ethnicity** | ***GSTM1*genotype distribution** | | | | ***GSTT1* genotype distribution** | | | | ***GSTP1* IIe105Val genotype distribution** | | | | | |
|---|---|---|---|---|---|---|---|---|---|---|---|---|---|---|---|
| | | **Case** | | **Control** | | **Case** | | **Control** | | **Case** | | | **Control** | | |
| | | **present** | **null** | **present** | **null** | **present** | **null** | **present** | **null** | **IIe/IIe** | **IIe/Val** | **Val/Val** | **IIe/IIe** | **IIe/Val** | **Val/Val** |
| Postmenopausal | | | | | | | | | | | | | | | |
| Ambrosone [8] 1999 | Caucasian | 84 | 93 | 116 | 117 | NA | NA | NA | NA | NA | NA | NA | NA | NA | NA |
| Helzlsouer [5] 1998 | Mixed | 30 | 56 | 51 | 36 | 63 | 23 | 68 | 19 | 47 | 35 | 6 | 35 | 37 | 14 |
| García-Closas [7] 1999 | Mixed | 179 | 178 | 177 | 169 | 301 | 57 | 288 | 60 | NA | NA | NA | NA | NA | NA |
| Millikan [12] 2000 | Mixed | 193 | 129 | 203 | 131 | 263 | 58 | 279 | 60 | 119 | 144 | 48 | 107 | 159 | 60 |
| Mitrunen [17] 2001 | Caucasian | 171 | 146 | 173 | 104 | 272 | 45 | 239 | 38 | 185 | 121 | 13 | 153 | 106 | 19 |
| Zhao [20] 2001 | Caucasian | NA | NA | NA | NA | NA | NA | NA | NA | 87 | 58 | 10 | 170 | 133 | 29 |
| Zheng T [23] 2002 | Mixed | 108 | 125 | 103 | 106 | 163 | 71 | 169 | 40 | NA | NA | NA | NA | NA | NA |
| Zheng W [25] 2002 | Caucasian | 102 | 100 | 232 | 249 | 113 | 39 | 263 | 62 | NA | NA | NA | NA | NA | NA |
| van der Hel [31] 2003 | Caucasian | 39 | 63 | 67 | 61 | NA | NA | NA | NA | NA | NA | NA | NA | NA | NA |
| Gago-Dominguez [39] 2004 | Asian | 98 | 82 | 248 | 218 | 114 | 66 | 262 | 204 | 115 | 65 | | 304 | 162 | |
| Park [41] 2004 | Asian | 39 | 41 | 57 | 65 | 40 | 40 | 76 | 46 | NA | NA | NA | NA | NA | NA |
| Kim [42] 2004 | Asian | NA | NA | NA | NA | NA | NA | NA | NA | 55 | 12 | 3 | 43 | 25 | 2 |
| Chacko [49] 2005 | Indian | 48 | 10 | 49 | 9 | 52 | 6 | 51 | 7 | NA | NA | NA | NA | NA | NA |
| Steck [55] 2007 | Mixed | 326 | 315 | 341 | 273 | 509 | 132 | 478 | 136 | 319 | 311 | | 286 | 316 | |
| Sakoda [65] 2008 | Asian | 153 | 168 | 185 | 165 | NA | NA | NA | NA | 197 | 115 | 9 | 216 | 121 | 12 |
| Lee [66] 2005 | Asian | NA | NA | NA | NA | NA | NA | NA | NA | 789 | 374 | 37 | 907 | 396 | 37 |
| Reding [70] 2009 | Mixed | 421 | 467 | 415 | 460 | 744 | 147 | 738 | 139 | 382 | 417 | 92 | 366 | 390 | 119 |
| Yu [71] 2009 | Asian | 168 | 215 | 159 | 194 | NA | NA | NA | NA | NA | NA | NA | NA | NA | NA |
| Saxena [72] 2009 | Indian | 108 | 118 | 147 | 82 | 164 | 62 | 165 | 64 | 77 | 101 | 48 | 149 | 65 | 18 |
| Antognelli [73] 2009 | Caucasian | NA | NA | NA | NA | NA | NA | NA | NA | 214 | 141 | 5 | 70 | 200 | 44 |
| MARIE-GENICA [78] 2010 | Caucasian | 1498 | 1614 | 2639 | 2807 | 2578 | 543 | 4585 | 871 | 1379 | 1371 | 391 | 2400 | 2502 | 565 |
| Cribb [83] 2011 | Caucasian | 55 | 72 | 184 | 197 | NA | NA | NA | NA | NA | NA | NA | NA | NA | NA |
| Cerne [85] 2011 | Caucasian | NA | NA | NA | NA | NA | NA | NA | NA | 233 | 243 | 54 | 130 | 101 | 39 |
| Hashemi [87] 2012 | Caucasian | 25 | 46 | 22 | 11 | 62 | 9 | 29 | 4 | 17 | 41 | 13 | 22 | 11 | 0 |
| Li SF [109] 2007 | Asian | 22 | 29 | 16 | 21 | 21 | 29 | 22 | 15 | NA | NA | NA | NA | NA | NA |
| Premenopausal | | | | | | | | | | | | | | | |
| Helzlsouer [5] 1998 | Mixed | 9 | 15 | 9 | 16 | 17 | 7 | 20 | 5 | 9 | 13 | 3 | 6 | 17 | 1 |
| García-Closas [7] 1999 | Mixed | 37 | 41 | 41 | 45 | 69 | 9 | 68 | 18 | NA | NA | NA | NA | NA | NA |
| Ambrosone [8] 1999 | Caucasian | 53 | 52 | 52 | 55 | NA | NA | NA | NA | NA | NA | NA | NA | NA | NA |
| Millikan [12] 2000 | Mixed | 193 | 131 | 161 | 133 | 278 | 52 | 249 | 44 | 120 | 142 | 43 | 88 | 145 | 36 |

| | | | | | | | | | | | | | | | |
|---|---|---|---|---|---|---|---|---|---|---|---|---|---|---|---|
| Mitrunen [17] 2001 | Caucasian | 89 | 75 | 105 | 96 | 139 | 25 | 176 | 25 | 98 | 57 | 9 | 113 | 75 | 15 |
| Matheson [22] 2002 | Caucasian | 66 | 91 | 80 | 77 | 113 | 44 | 139 | 18 | NA | NA | NA | NA | NA | NA |
| Zheng T [23] 2002 | Mixed | 44 | 40 | 57 | 67 | 60 | 24 | 90 | 34 | NA | NA | NA | NA | NA | NA |
| van der Hel [31] 2003 | Caucasian | 57 | 70 | 67 | 68 | NA | NA | NA | NA | NA | NA | NA | NA | NA | NA |
| Park [41] 2004 | Asian | 45 | 75 | 80 | 87 | 59 | 61 | 92 | 75 | NA | NA | NA | NA | NA | NA |
| Kim [42] 2004 | Asian | NA | NA | NA | NA | NA | NA | NA | NA | 67 | 32 | 2 | 70 | 27 | 4 |
| Chacko [49] 2005 | Indian | 24 | 30 | 35 | 19 | 45 | 9 | 51 | 3 | NA | NA | NA | NA | NA | NA |
| Steck [55] 2007 | Mixed | 163 | 147 | 181 | 158 | 242 | 68 | 262 | 77 | 156 | 149 | | 164 | 171 | |
| Sakoda [65] 2008 | Asian | 141 | 153 | 265 | 263 | NA | NA | NA | NA | 181 | 100 | 11 | 353 | 156 | 18 |
| Lee [66] 2005 | Asian | NA | NA | NA | NA | NA | NA | NA | NA | 1161 | 579 | 86 | 1096 | 553 | 48 |
| Yu [71] 2009 | Asian | 227 | 407 | 234 | 316 | NA | NA | NA | NA | NA | NA | NA | NA | NA | NA |
| Saxena [72] 2009 | Indian | 83 | 97 | 114 | 60 | 146 | 34 | 150 | 24 | 70 | 92 | 18 | 51 | 106 | 14 |
| Antognelli [73] 2009 | Caucasian | NA | NA | NA | NA | NA | NA | NA | NA | 101 | 76 | 10 | 58 | 140 | 32 |
| Cribb [83] 2011 | Caucasian | 39 | 41 | 100 | 140 | NA | NA | NA | NA | NA | NA | NA | NA | NA | NA |
| Hashemi [87] 2012 | Caucasian | 23 | 40 | 59 | 60 | 54 | 9 | 111 | 8 | 19 | 31 | 13 | 75 | 41 | 3 |
| Li SF [109] 2007 | Asian | 13 | 29 | 44 | 49 | 19 | 22 | 50 | 41 | NA | NA | NA | NA | NA | NA |

NA: not available
