# Supplementary material for: Individual and combined effects of GSTM1, GSTT1, and GSTP1 polymorphisms on breast cancer risk: A meta-analysis and re-analysis of systematic meta-analyses
Source: PLoS One. 2020 Mar 10;15(3):e0216147. doi: 10.1371/journal.pone.0216147 (PMC7064184; doi:10.1371/journal.pone.0216147)
Supplement: S7 Table — (PDF) [file pone.0216147.s007.pdf]

First author/Year	Ethnicity	*GSTM1*genotype distribution				*GSTT1* genotype distribution				*GSTP1* IIe105Val genotype distribution					
		Case		Control		Case		Control		Case			Control		
		present	null	present	null	present	null	present	null	IIe/IIe	IIe/Val	Val/Val	IIe/IIe	IIe/Val	Val/Val
Current smoking															
Kelsey [3] 1997	Mixed	12	18	12	12	NA	NA	NA	NA	NA	NA	NA	NA	NA	NA
Matheson [22] 2002	Caucasian	13	19	18	19	20	12	35	2	NA	NA	NA	NA	NA	NA
Zheng T [23] 2002	Mixed	18	33	27	29	37	14	44	14	NA	NA	NA	NA	NA	NA
Zheng W [25] 2002	Caucasian	11	11	23	35	11	5	30	10	NA	NA	NA	NA	NA	NA
van der Hel [31] 2003	Caucasian	69	84	84	75	NA	NA	NA	NA	NA	NA	NA	NA	NA	NA
Past smoking															
Kelsey [3] 1997	Mixed	50	57	55	50	NA	NA	NA	NA	NA	NA	NA	NA	NA	NA
Bailey [6] 1998	Caucasian	27	33	26	33	47	13	46	13	NA	NA	NA	NA	NA	NA
Bailey [6] 1998	African	10	8	16	14	17	1	24	6	NA	NA	NA	NA	NA	NA
Matheson [22] 2002	Caucasian	20	30	19	18	35	15	32	5	NA	NA	NA	NA	NA	NA
Zheng T [23] 2002	Mixed	63	70	56	68	95	40	105	25	NA	NA	NA	NA	NA	NA
Zheng W [25] 2002	Caucasian	22	20	52	40	24	10	51	6	NA	NA	NA	NA	NA	NA
McCarty [69] 2009	Mixed	278	252	300	253	423	112	454	109	275	278		309	284	
No-smoking															
Kelsey [3] 1997	Mixed	59	44	57	53	NA	NA	NA	NA	NA	NA	NA	NA	NA	NA
Bailey [6] 1998	Caucasian	45	56	37	61	68	33	70	28	NA	NA	NA	NA	NA	NA
Bailey [6] 1998	African	25	6	19	10	23	8	18	11	NA	NA	NA	NA	NA	NA
García-Closas [7] 1999	Mixed	NA	NA	NA	NA	175	23	181	39	NA	NA	NA	NA	NA	NA
Matheson [22] 2002	Caucasian	33	42	43	40	58	17	72	11	NA	NA	NA	NA	NA	NA
Zheng T [23] 2002	Mixed	66	62	63	76	90	41	111	35	NA	NA	NA	NA	NA	NA
Zheng W [25] 2002	Caucasian	68	67	156	168	76	23	177	46	NA	NA	NA	NA	NA	NA
van der Hel [31] 2003	Caucasian	27	49	50	54	NA	NA	NA	NA	NA	NA	NA	NA	NA	NA
Wu [51] 2006	Asian	139	123	122	103	NA	NA	NA	NA	NA	NA	NA	NA	NA	NA
Van Emburgh [62] 2008	Caucasian	107	125	107	162	190	42	222	47	204		24	205		21
Van Emburgh [62] 2008	African	19	9	33	15	21	7	42	6	23		5	35		9
McCarty [69] 2009	Mixed	227	218	247	201	351	97	341	112	235	239		242	234	

NA: not available
