# Supplementary material for: Individual and combined effects of GSTM1, GSTT1, and GSTP1 polymorphisms on breast cancer risk: A meta-analysis and re-analysis of systematic meta-analyses
Source: PLoS One. 2020 Mar 10;15(3):e0216147. doi: 10.1371/journal.pone.0216147 (PMC7064184; doi:10.1371/journal.pone.0216147)
Supplement: S8 Table — (PDF) [file pone.0216147.s008.pdf]

First author/Year	Case/control	+ +		+ −		− +		(+ −) + (− +)		(+ −) + (− +) + (+ +)		− −		All risk genotypes	
		Case	Control	Case	Control	Case	Control	Case	Control	Case	Control	Case	Control	Case	Control
Kimi [102] 2016	22/10	2	2	2	3	6	4	8	7	10	9	12	1	20	8
Chirilă [96] 2014	59/39	10	18	NA	NA	NA	NA	41	19	51	37	8	2	49	21
Possuelo [94] 2013	49/49	5	2	NA	NA	NA	NA	NA	NA	NA	NA	NA	NA	44	47
Hashemi [87] 2012	134/152	48	81	0	0	71	59	71	59	119	140	15	12	86	71
Ramalhinho [88] 2012	101/121	20	61	15	15	34	36	49	51	69	112	32	9	81	60
Kostrykina [68] 2009	695/263	306	112	61	28	257	107	318	135	624	247	71	16	389	151
Saxena [72] 2009	399/396	141	202	45	61	162	106	207	167	348	369	51	27	258	194
Unlu [67] 2008	65/108	21	40	11	33	17	24	28	57	49	97	16	11	44	68
Rajkumar [64] 2008	250/500	152	324	33	66	55	91	88	157	240	481	10	19	98	176
Steck [55] 2007	971/998	394	400	107	144	368	378	475	522	869	922	102	76	577	598
Spurdle [56] 2007	1235/659	480	267	83	63	541	283	624	346	1104	613	131	46	755	392
Cui [115] 2007	105/100	33	56	20	19	23	22	43	41	76	97	29	3	72	44
Li SF [109] 2007	91/127	16	29	19	29	24	42	43	71	59	100	32	27	75	98
Chang [52] 2006	189/417	35	82	47	109	43	126	90	235	125	317	64	100	154	335
Vogl [43] 2004	1186/849	460	327	NA	NA	NA	NA	607	412	1067	739	119	110	726	522
Gago-Dominguez [39] 2004	180/466	NA	NA	NA	NA	NA	NA	NA	NA	146	370	34	96	NA	NA
Egan [40] 2004	1132/1193	245	263	252	253	332	340	584	593	829	856	303	337	887	930
Park [41] 2004	202/299	33	70	NA	NA	NA	NA	117	165	150	235	50	54	167	219
McCready [37] 2004	70/69	NA	NA	NA	NA	NA	NA	NA	NA	57	60	8	5	NA	NA
Zheng T [32] 2003	312/319	100	115	47	31	119	133	166	164	266	279	46	40	212	204
Khedhaier [30] 2003	309/242	NA	NA	NA	NA	NA	NA	NA	NA	254	206	55	36	NA	NA
da Fonte de Amorim [24] 2002	79/123	NA	NA	NA	NA	NA	NA	NA	NA	74	107	5	16	NA	NA
da Fonte de Amorim [24] 2002	49/133	NA	NA	NA	NA	NA	NA	NA	NA	45	128	4	5	NA	NA
Zheng W [25] 2002	152/325	47	131	NA	NA	NA	NA	NA	NA	NA	NA	NA	NA	105	194
Gudmundsdottir [15] 2001	500/395	179	143	48	38	222	171	270	209	449	452	51	43	321	252
Dialyna [16] 2001	207/171	85	76	14	6	92	78	106	84	191	160	16	11	122	95
Mitrunen [17] 2001	481/478	219	236	NA	NA	NA	NA	233	221	452	457	29	21	262	242
Millikan [12] 2000	570/555	278	265	60	53	194	196	254	249	532	514	38	41	292	290
Curran [11] 2000	128/128	45	48	11	8	56	60	67	68	112	116	16	12	83	80
García-Closas [7] 1999	465/464	198	192	35	45	197	192	232	237	430	429	35	35	267	272
Helzlsouer [5] 1998	110/112	26	47	13	13	54	41	67	54	93	101	17	11	84	65

+ −: *GSTM1* present/*GSTT1* null; − +: *GSTM1* null/*GSTT1* present; − −: *GSTM1* null/*GSTT1* null; + +: *GSTM1* present/*GSTT1* present; NA: not available
