# Supplementary material for: Individual and combined effects of GSTM1, GSTT1, and GSTP1 polymorphisms on breast cancer risk: A meta-analysis and re-analysis of systematic meta-analyses
Source: PLoS One. 2020 Mar 10;15(3):e0216147. doi: 10.1371/journal.pone.0216147 (PMC7064184; doi:10.1371/journal.pone.0216147)
Supplement: S9 Table — (PDF) [file pone.0216147.s009.pdf]

First author/Year	Case/ control	+ +		+ −		− +		(+ −) + (− +)		(+ −) + (− +) + (+ +)		− −		All risk genotypes	
		**Case**	**Control**	**Case**	**Control**	**Case**	**Control**	**Case**	**Control**	**Case**	**Control**	**Case**	**Control**	**Case**	**Control**
Postmenopausal															
Gago-Dominguez [39] 2004	180/466	NA	NA	NA	NA	NA	NA	NA	NA	146	370	34	96	NA	NA
Khedhaier [30] 2003	112/242	NA	NA	NA	NA	NA	NA	NA	NA	95	206	17	36	NA	NA
Steck [55] 2007	641/614	262	247	64	94	247	231	311	325	573	572	68	42	379	367
Zheng W [25] 2002	152/325	47	131	NA	NA	NA	NA	NA	NA	NA	NA	NA	NA	105	194
Mitrunen [17] 2001	317/277	142	147	NA	NA	NA	NA	159	118	301	265	16	12	175	130
García-Closas [7] 1999	357/346	148	142	31	35	152	144	183	179	331	321	26	25	209	204
Park [41] 2004	80/122	16	34	NA	NA	NA	NA	47	65	63	99	17	23	64	88
Zheng T [23] 2002	229/201	73	74	31	21	87	89	118	110	191	184	38	17	156	127
Premenopausal															
Khedhaier [30] 2003	194/242	NA	NA	NA	NA	NA	NA	NA	NA	159	206	38	36	NA	NA
Steck [55] 2007	310/339	125	136	38	45	117	126	155	171	280	307	30	32	185	203
Mitrunen [17] 2001	164/201	77	89	NA	NA	NA	NA	74	103	150	192	13	9	87	112
García-Closas [7] 1999	108/118	50	50	4	10	45	48	49	58	99	108	9	10	58	68
Park [41] 2004	120/167	17	36	NA	NA	NA	NA	70	100	87	136	33	31	103	131
Zheng T [23] 2002	83/118	27	41	16	10	32	44	48	54	75	95	8	23	56	77

+ −: *GSTM1* present/*GSTT1* null; − +: *GSTM1* null/*GSTT1* present; − −: *GSTM1* null/*GSTT1* null; + +: *GSTM1* present/*GSTT1* present; NA: not available
