# Supplementary material for: Individual and combined effects of GSTM1, GSTT1, and GSTP1 polymorphisms on breast cancer risk: A meta-analysis and re-analysis of systematic meta-analyses
Source: PLoS One. 2020 Mar 10;15(3):e0216147. doi: 10.1371/journal.pone.0216147 (PMC7064184; doi:10.1371/journal.pone.0216147)
Supplement: S10 Table — (PDF) [file pone.0216147.s010.pdf]

First author/Year	Ethnicity	*GSTM1* present/ *GSTP1* IIe/IIe		*GSTM1* null/ *GSTP1* IIe/IIe		*GSTM1* present/*GSTP1* Val*		Total one risk genotype		*GSTM1* null/ *GSTP1* Val*		All risk genotypes	
		**Case**	**Control**	**Case**	**Control**	**Case**	**Control**	**Case**	**Control**	**Case**	**Control**	**Case**	**Control**
Hashemi [87] 2012	Caucasian	18	53	16	43	30	28	46	71	70	28	116	99
Ramalhinho [82] 2011	Caucasian	19	29	24	19	12	32	36	51	30	22	66	73
Saxena [72] 2009	Indian	84	139	60	58	102	124	162	182	153	75	315	257
Unlu [67] 2008	Caucasian	14	38	11	13	18	35	29	48	22	22	51	70
Rajkumar [64] 2008	Indian	95	180	23	50	90	210	113	260	42	60	155	320
Sakoda [65] 2008	Asian	183	293	195	276	110	157	305	433	125	150	430	583
Steck [55] 2007	Mixed	244	254	241	216	250	278	491	494	220	233	711	727
Chang [52] 2006	Asian	58	127	65	160	24	64	89	224	42	66	131	290
Vogl [43] 2004	Mixed	400	235	NA	NA	NA	NA	459	256	47	24	506	280
Egan [40] 2004	Asian	314	336	405	459	183	180	588	639	230	218	818	857
Sarmanová [38] 2004	Caucasian	30	79	NA	NA	NA	NA	138	143	68	87	206	230
Gudmundsdottir [15] 2001	Caucasian	97	95	105	82	130	86	235	168	168	132	403	300
Millikan [12] 2000	Mixed	122	98	101	86	216	220	317	306	131	151	448	457
Curran [11] 2000	Caucasian	27	30	35	29	29	26	64	55	37	43	101	98
Helzlsouer [5] 1998	Mixed	18	31	23	25	21	29	44	54	48	27	92	81

One risk genotype: *GSTM1* null/*GSTP1* IIe/IIe & *GSTM1* present/ *GSTP1* Val*; Val*: IIe/Val or Val/Val
