# Supplementary material for: Individual and combined effects of GSTM1, GSTT1, and GSTP1 polymorphisms on breast cancer risk: A meta-analysis and re-analysis of systematic meta-analyses
Source: PLoS One. 2020 Mar 10;15(3):e0216147. doi: 10.1371/journal.pone.0216147 (PMC7064184; doi:10.1371/journal.pone.0216147)
Supplement: S11 Table — (PDF) [file pone.0216147.s011.pdf]

First author/Year	Ethnicity	*GSTT1* present/ *GSTP1* IIe/IIe		*GSTT1* null/*GSTP1* IIe/IIe		*GSTT1* present/ *GSTP1* val*		Total one risk genotype		*GSTT1* null/ *GSTP1* val*		All risk genotypes	
		**Case**	**Control**	**Case**	**Control**	**Case**	**Control**	**Case**	**Control**	**Case**	**Control**	**Case**	**Control**
Hashemi [87] 2012	Caucasian	33	90	1	6	86	50	87	56	14	6	101	62
Ramalhinho [82] 2011	Caucasian	22	38	21	10	26	46	47	56	16	8	63	64
Saxena [72] 2009	Indian	112	149	32	48	191	159	223	207	64	40	287	247
Unlu [67] 2008	Caucasian	13	26	12	25	25	38	37	63	15	19	52	82
Rajkumar [64] 2008	Indian	101	185	17	45	106	230	123	275	26	40	149	315
Steck [55] 2007	Mixed	382	386	109	94	377	392	486	486	95	122	581	608
Chang [52] 2006	Asian	53	137	70	150	25	71	95	221	41	59	136	280
Vogl [43] 2004	Mixed	647	375	NA	NA	NA	NA	225	135	16	5	241	140
Egan [40] 2004	Asian	361	401	358	394	216	202	574	596	197	196	771	792
Gudmundsdottir [15] 2001	Caucasian	166	138	36	39	235	176	271	215	63	42	334	257
Millikan [12] 2000	Mixed	187	162	36	22	285	299	321	321	62	72	383	393
Curran [11] 2000	Caucasian	47	48	15	11	54	60	69	71	12	9	81	80
Helzlsouer [5] 1998	Mixed	28	47	13	9	52	41	65	50	17	15	82	65

One risk genotype: *GSTM1* null/ *GSTP1* IIe/IIe & *GSTM1* present/ *GSTP1* Val*, Val*: Ile/Val or Val/Val; NA: not available
