# Supplementary material for: Individual and combined effects of GSTM1, GSTT1, and GSTP1 polymorphisms on breast cancer risk: A meta-analysis and re-analysis of systematic meta-analyses
Source: PLoS One. 2020 Mar 10;15(3):e0216147. doi: 10.1371/journal.pone.0216147 (PMC7064184; doi:10.1371/journal.pone.0216147)
Supplement: S12 Table — (PDF) [file pone.0216147.s012.pdf]

First author/Year	*M1* present/*T1* present/*P1* IIe/IIe		*M1* null/*T1* present/*GSTP1* IIe/IIe		*M1* present/*T1* null/*P1* IIe/IIe		*M1* present/*T1* present/*P1* Val [1]		All one high-risk genotype		*M1* null/*T1* null/*P1* IIe/IIe		*M1* null/*T1* present/*P1* Val [1]		*M1* present/*T1* null/*P1* Val [1]		All two high-risk genotype		*M1* null/*T1* null/*P1* Val [1]	
	**Case**	**Control**	**Case**	**Control**	**Case**	**Control**	**Case**	**Control**	**Case**	**Control**	**Case**	**Control**	**Case**	**Control**	**Case**	**Control**	**Case**	**Control**	**Case**	**Control**
Hashemi [87] 2012	18	53	15	37	0	0	30	28	45	65	1	6	56	22	0	0	57	28	14	6
Ramalhinho [82] 2011	10	21	12	17	9	8	8	29	29	54	12	2	18	17	4	3	34	22	12	5
Saxena [72] 2009	70	106	42	43	14	33	71	96	127	172	18	15	120	63	31	28	169	106	33	12
Unlu [67] 2008	8	18	5	8	6	20	13	22	24	50	6	5	12	16	5	13	23	34	10	6
Rajkumar [64] 2008	78	144	23	41	17	36	74	180	114	257	0	9	32	50	16	30	48	89	10	10
Steck [55] 2007	184	187	190	188	58	65	201	203	449	456	51	28	172	185	47	74	270	284	48	48
Chang [52] 2006	26	53	27	84	32	74	9	29	68	187	38	76	16	42	15	35	69	153	26	24
Vogl [43] 2004	313	188	NA	NA	NA	NA	NA	NA	446	250	NA	NA	NA	NA	NA	NA	114	69	9	4
Egan [40] 2004	154	163	207	238	160	173	91	100	458	511	198	221	125	102	92	80	415	403	105	116
Gudmundsdottir [15] 2001	76	74	90	64	21	21	103	69	214	154	15	18	132	107	27	17	174	142	36	25
Millikan [12] 2000	104	86	83	76	18	12	174	179	275	267	18	10	111	120	42	41	171	171	20	31
Curran [11] 2000	22	24	25	24	5	6	23	24	53	54	10	5	31	36	6	2	47	43	6	7
Helzlsouer [5] 1998	12	27	16	20	6	4	14	20	36	44	7	5	38	21	7	9	52	35	10	6

Val[1] IIe/Val or Val/Val ; All one high-risk genotype: *M1* null/*T1* present/*P1* IIe/IIe + *M1* present/*T1* null/*P1* IIe/IIe + *M1* present/*T1* present/*P1* Val[1]; All two high-risk genotype: *M1* null/*T1* null/*P1* IIe/IIe + *M1* null/*T1* present/*P1* Val[1] + *M1* present/*T1* null/*P1* Val[1]; NA: not available
