# Supplementary figures and images for: Individual and combined effects of GSTM1, GSTT1, and GSTP1 polymorphisms on breast cancer risk: A meta-analysis and re-analysis of systematic meta-analyses
Source: PLoS One. 2020 Mar 10;15(3):e0216147. doi: 10.1371/journal.pone.0216147 (PMC7064184; doi:10.1371/journal.pone.0216147)

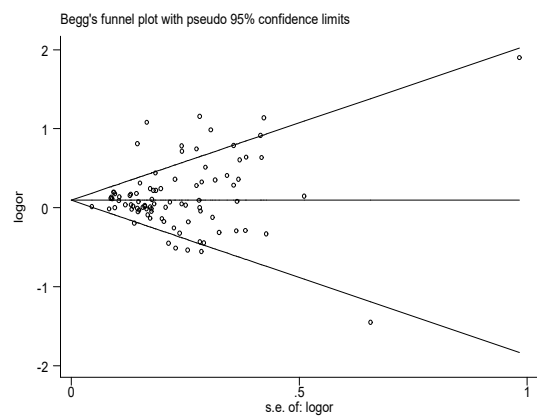

Supplement: S1 Fig — (PDF) [file pone.0216147.s013.pdf]

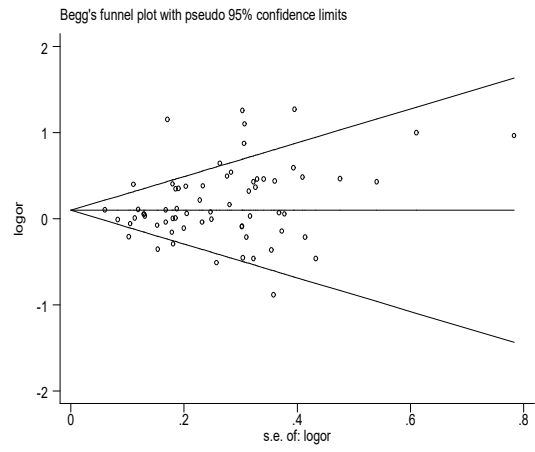

Supplement: S2 Fig — (PDF) [file pone.0216147.s014.pdf]

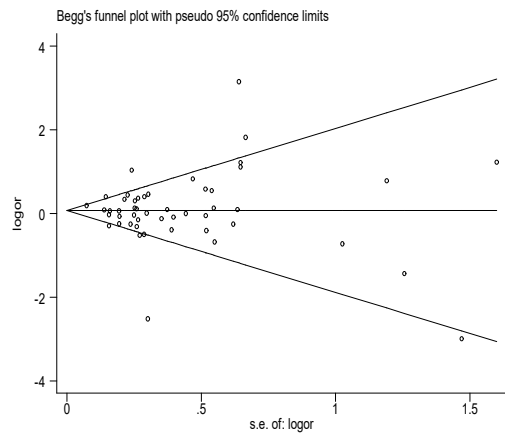

Supplement: S3 Fig — (PDF) [file pone.0216147.s015.pdf]

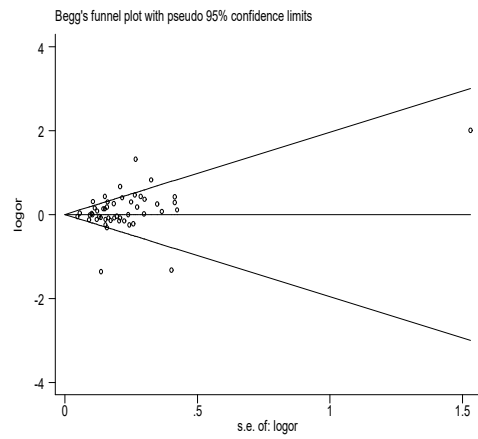

Supplement: S4 Fig — (PDF) [file pone.0216147.s016.pdf]

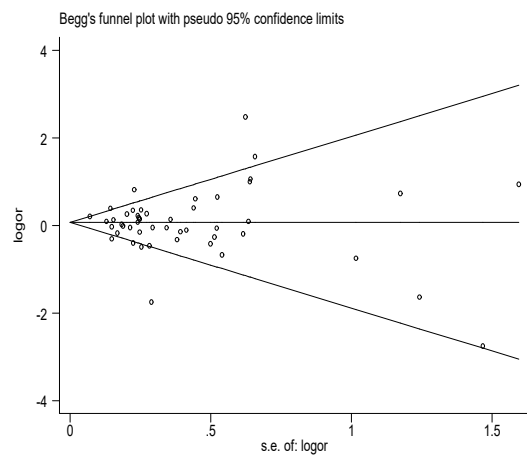

Supplement: S5 Fig — (PDF) [file pone.0216147.s017.pdf]

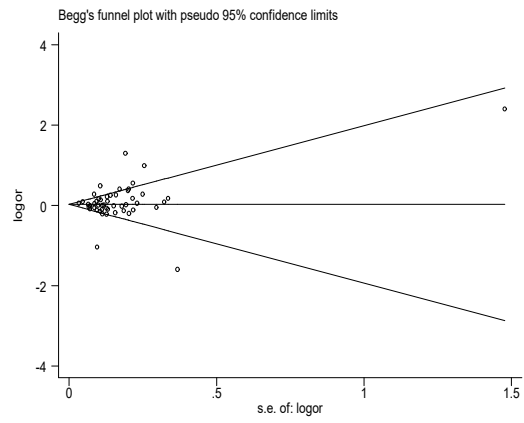

Supplement: S6 Fig — (PDF) [file pone.0216147.s018.pdf]

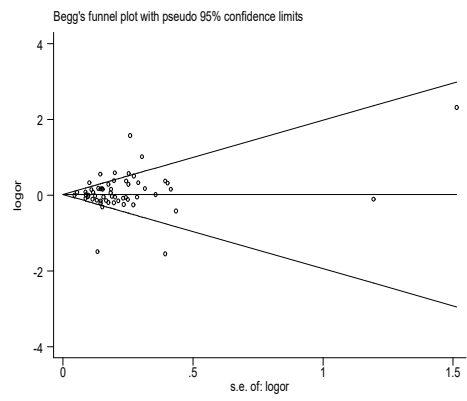

Supplement: S7 Fig — (PDF) [file pone.0216147.s019.pdf]

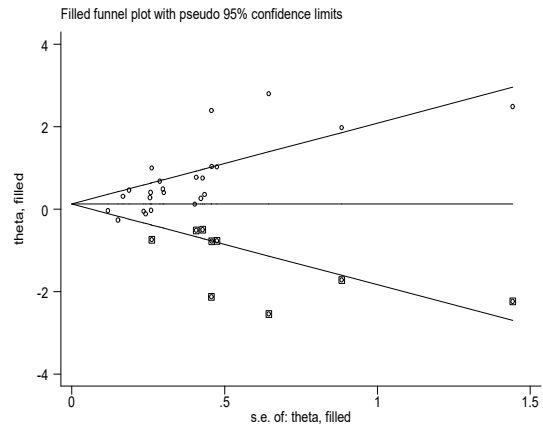

Supplement: S8 Fig — (PDF) [file pone.0216147.s020.pdf]

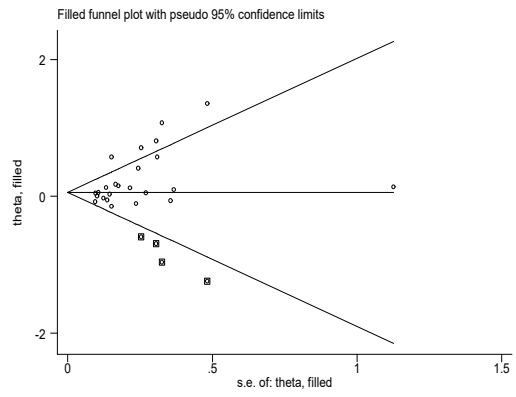

Supplement: S9 Fig — (PDF) [file pone.0216147.s021.pdf]

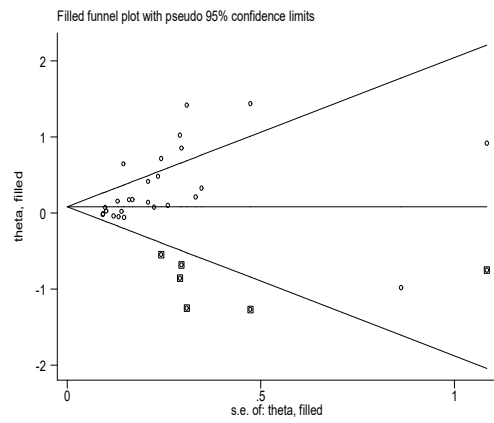

Supplement: S10 Fig — (PDF) [file pone.0216147.s022.pdf]

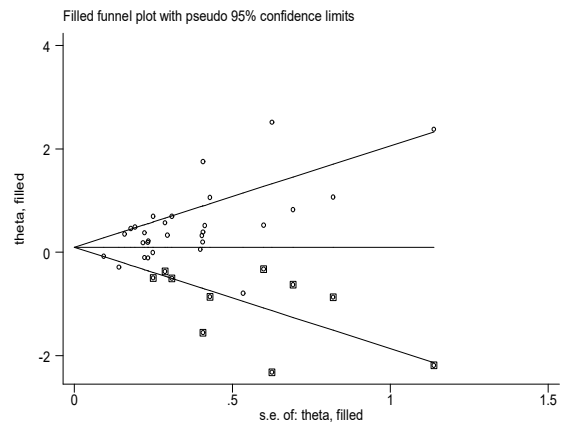

Supplement: S11 Fig — (PDF) [file pone.0216147.s023.pdf]

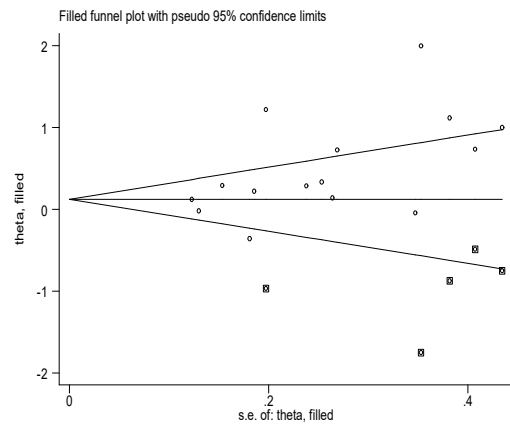

Supplement: S12 Fig — (PDF) [file pone.0216147.s024.pdf]

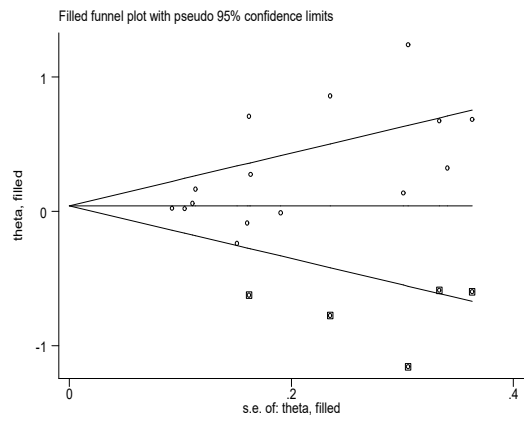

Supplement: S13 Fig — (PDF) [file pone.0216147.s025.pdf]

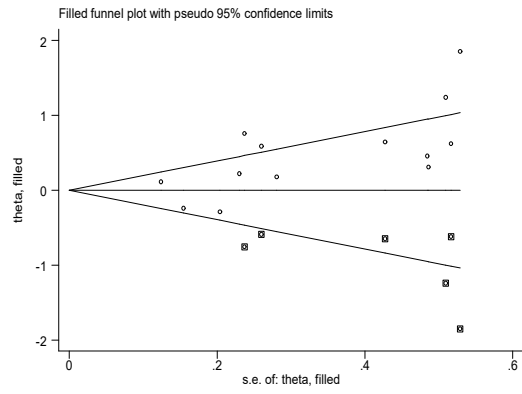

Supplement: S14 Fig — (PDF) [file pone.0216147.s026.pdf]

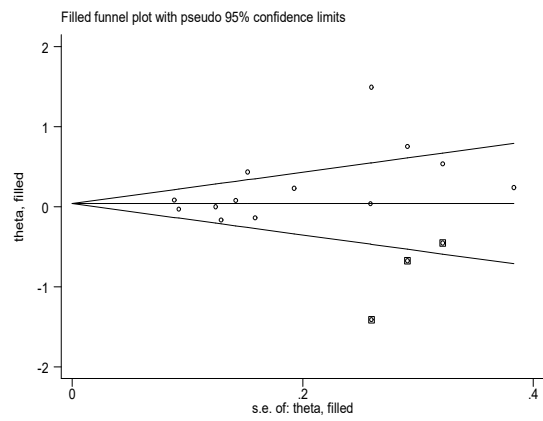

Supplement: S15 Fig — (PDF) [file pone.0216147.s027.pdf]

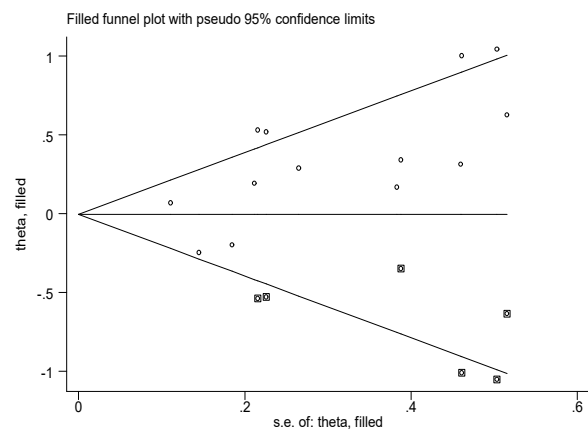

Supplement: S16 Fig — (PDF) [file pone.0216147.s028.pdf]

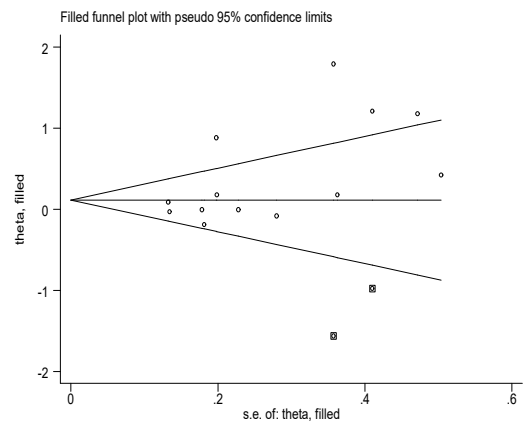

Supplement: S17 Fig — (PDF) [file pone.0216147.s029.pdf]

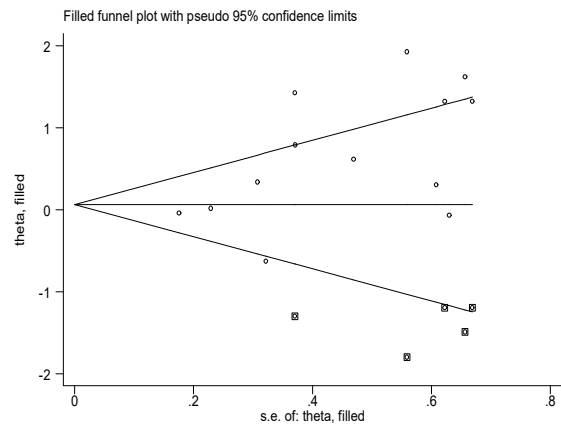

Supplement: S18 Fig — (PDF) [file pone.0216147.s030.pdf]

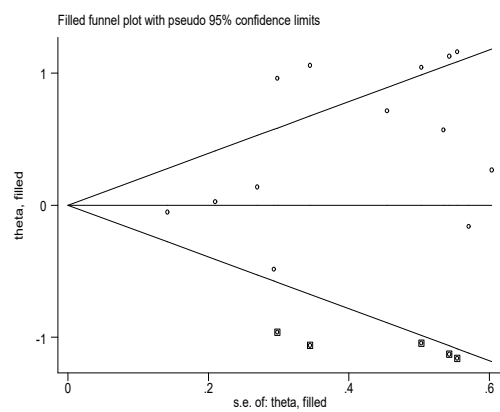

Supplement: S19 Fig — (PDF) [file pone.0216147.s031.pdf]
