# Supplementary material for: Individual and combined effects of GSTM1, GSTT1, and GSTP1 polymorphisms on breast cancer risk: A meta-analysis and re-analysis of systematic meta-analyses
Source: PLoS One. 2020 Mar 10;15(3):e0216147. doi: 10.1371/journal.pone.0216147 (PMC7064184; doi:10.1371/journal.pone.0216147)
Supplement: S1 Appendix — (PDF) [file pone.0216147.s032.pdf]

Supplemental references

1. Zhong S, Wyllie AH, Barnes D, Wolf CR, Spurr NK. Relationship between the GSTM1 genetic polymorphism and susceptibility to bladder, breast and colon cancer. Carcinogenesis. 1993;14:1821–4. PMID: 8403204
2. Ambrosone CB, Freudenheim JL, Graham S, Marshall JR, Vena JE, Brasure JR, et al. Cytochrome P4501A1 and glutathione S-transferase (M1) genetic polymorphisms and postmenopausal breast cancer risk. Cancer Res. 1995;55:3483–5. PMID: 7627950
3. Kelsey KT, Hankinson SE, Colditz GA, Springer K, Garcia-Closas M, Spiegelman D, et al. Glutathione S-transferase class mu deletion polymorphism and breast cancer: results from prevalent versus incident cases. Cancer Epidemiol Biomarkers Prev. 1997;6: 511–5. PMID: 9232338
4. Maugard CM, Charrier J, Bignon YJ. Allelic deletion at glutathione S-transferase M1 locus and its association with breast cancer susceptibility. Chem Biol Interact. 1998;111–112: 365–75. PMID: 9679567
5. Helzlsouer KJ, Selmin O, Huang HY, Strickland PT, Hoffman S, Alberg AJ, et al. Association between glutathione S-transferase M1, P1, and T1 genetic polymorphisms and development of breast cancer. J Natl Cancer Inst. 1998;90: 512–8. PMID: 9539246
6. Bailey LR, Roodi N, Verrier CS, Yee CJ, Dupont WD, Parl FF. Breast cancer and CYPIA1, GSTM1, and GSTT1 polymorphisms: evidence of a lack of association in Caucasians and African Americans. Cancer Res. 1998;58: 65–70. PMID: 9426059
7. García-Closas M, Kelsey KT, Hankinson SE, Spiegelman D, Springer K, Willett WC, et al. Glutathione S-transferase mu and theta polymorphisms and breast cancer susceptibility. J Natl Cancer Inst. 1999;91:1960–4. PMID: 10564681
8. Ambrosone CB, Coles BF, Freudenheim JL, Shields PG. Glutathione-S-transferase (GSTM1) genetic polymorphisms do not affect human breast cancer risk, regardless of dietary antioxidants. J Nutr. 1999;129:565S–8S. https://doi.org/10.1093/jn/129.2.565S PMID: 10064333
9. Charrier J, Maugard CM, Le Mevel B, Bignon YJ. Allelotype influence at glutathione S-transferase M1 locus on breast cancer susceptibility. Br J Cancer. 1999;79:346–53. https://doi.org/10.1038/sj.bjc.6690055 PMID: 9888479
10. Park SK, Yoo KY, Lee SJ, Kim SU, Ahn SH, Noh DY, et al. Alcohol consumption, glutathione S-transferase M1 and T1 genetic polymorphisms and breast cancer risk. Pharmacogenetics. 2000;10:301–9. PMID: 10862521
11. Curran JE, Weinstein SR, Griffiths LR. Polymorphisms of glutathione S-transferase genes (GSTM1, GSTP1 and GSTT1) and breast cancer susceptibility. Cancer Lett. 2000;153:113–20. PMID: 10779639
12. Millikan R, Pittman G, Tse CK, Savitz DA, Newman B, Bell D. Glutathione S-transferases M1, T1, and P1 and breast cancer. Cancer Epidemiol Biomarkers Prev. 2000;9:567–73. PMID: 15342448
13. Rundle A, Tang D, Zhou J, Cho S, Perera F. The association between glutathione S-transferase M1 genotype and polycyclic aromatic hydrocarbon-DNA adducts in breast tissue. Cancer Epidemiol Biomarkers Prev. 2000;9: 1079–85. PMID: 11045791
14. Xiong P, Bondy ML, Li D, Shen H, Wang LE, Singletary SE, et al. Sensitivity to benzo(a)pyrene diol-epoxide associated with risk of breast cancer in young women and modulation by glutathione S-transferase polymorphisms: a case-control study. Cancer Res. 2001;61: 8465–9. PMID: 11731429
15. Gudmundsdottir K, Tryggvadottir L, Eyfjord JE. GSTM1, GSTT1, and GSTP1 genotypes in relation to breast cancer risk and frequency of mutations in the p53 gene. Cancer Epidemiol Biomarkers Prev. 2001;10: 1169–73. PMID: 11700265
16. Dialyna IA, Arvanitis DA, Spandidos DA. Genetic polymorphisms and transcriptional pattern analysis of CYP1A1, AhR, GSTM1, GSTP1 and GSTT1 genes in breast cancer. Int J Mol Med. 2001;8: 79–87. PMID: 11408954
17. Mitrunen K, Jourenkova N, Kataja V, Eskelinen M, Kosma VM, Benhamou S, et al. Glutathione S-transferase M1, M3, P1, and T1 genetic polymorphisms and susceptibility to breast cancer. Cancer Epidemiol Biomarkers Prev. 2001;10:229–36. PMID: 11303592
18. Krajinovic M, Ghadirian P, Richer C, Sinnett H, Gandini S, Perret C, et al. Genetic susceptibility to breast cancer in French-Canadians: role of carcinogen-metabolizing enzymes and gene-environment interactions. Int J Cancer. 2001;92:220–5. PMID: 11291049
19. Maugard CM, Charrier J, Pitard A, Campion L, Akande O, Pleasants L, et al. Genetic polymorphism at the glutathione S-transferase (GST) P1 locus is a breast cancer risk modifier. Int J Cancer. 2001;91:334–9. PMID: 11169956
20. Zhao M, Lewis R, Gustafson DR, Wen WQ, Cerhan JR, Zheng W. No apparent association of GSTP1 A(313)G polymorphism with breast cancer risk among postmenopausal Iowa women. Cancer Epidemiol Biomarkers Prev. 2001;10:1301–2. PMID: 11751449
21. Mitrunen K, Kataja V, Eskelinen M, Kosma VM, Kang D, Benhamou S, et al. Combined COMT and GST genotypes and hormone replacement therapy associated breast cancer risk. Pharmacogenetics. 2002;12:67–72.

PMID: 11773866
22. Matheson MC, Stevenson T, Akbarzadeh S, Propert DN. GSTT1 null genotype increases risk of premenopausal breast cancer. Cancer Lett. 2002;181:73–9. PMID: 12430181
23. Zheng T, Holford TR, Zahm SH, Owens PH, Boyle P, Zhang Y, et al. Cigarette smoking, glutathione-s-transferase M1 and t1 genetic polymorphisms, and breast cancer risk (United States). Cancer Causes Control. 2002;13:637–45. PMID: 12296511
24. da Fonte de Amorim L, Rossini A, Mendonça G, Lotsch P, de Almeida Simão T, de Moura Gallo C, et al. CYP1A1, GSTM1, and GSTT1 polymorphisms and breast cancer risk in Brazilian women. Cancer Lett. 2002;181:179–86. PMID: 12175533
25. Zheng W, Wen WQ, Gustafson DR, Gross M, Cerhan JR, Folsom AR. GSTM1 and GSTT1 polymorphisms and postmenopausal breast cancer risk. Breast Cancer Res Treat. 2002;74:9–16. PMID: 12150456
26. Wu FY, Lee YJ, Chen DR, Kuo HW. Association of DNA-protein crosslinks and breast cancer. Mutat Res. 2002;501:69–78. PMID: 11934439
27. Siegelmann-Danieli N, Buetow KH. Significance of genetic variation at the glutathione S-transferase M1 and NAD(P)H:quinone oxidoreductase 1 detoxification genes in breast cancer development. Oncology. 2002;62:39–45. https://doi.org/10.1159/000048245 PMID: 11810042
28. Li D, Walcott FL, Chang P, Zhang W, Zhu J, Petrulis E, et al. Genetic and environmental determinants on tissue response to in vitro carcinogen exposure and risk of breast cancer. Cancer Res. 2002;62:4566–70. PMID: 12183407
29. Geng Y, Chen Y, Zhao W, Wang L, Wang L, Yang J. Mutations and Polymorphisms of GSTP1 Gene in Benign and Malignant Breast Lesions. Chinese Journal of Clinical Oncology 2010; 37:1028–31.
30. Khedhaier A, Remadi S, Corbex M, Ahmed SB, Bouaouina N, Mestiri S, et al. Glutathione S-transferases (GSTT1 and GSTM1) gene deletions in Tunisians: susceptibility and prognostic implications in breast carcinoma. Br J Cancer. 2003;89:1502–7. https://doi.org/10.1038/sj.bjc.6601292 PMID: 14562023
31. van der Hel OL, Peeters PH, Hein DW, Doll MA, Grobbee DE, Kromhout D, et al. NAT2 slow acetylation and GSTM1 null genotypes may increase postmenopausal breast cancer risk in long-term smoking women. Pharmacogenetics. 2003;13:399–407. https://doi.org/10.1097/01.fpc.0000054106.48725.87 PMID: 12835615
32. Zheng T, Holford TR, Zahm SH, Owens PH, Boyle P, Zhang Y, et al. Glutathione S-transferase M1 and T1 genetic polymorphisms, alcohol consumption and breast cancer risk. Br J Cancer. 2003;88:58–62. https://doi.org/10.1038/sj.bjc.6600708 PMID: 12556960
33. Kang D. Genetic polymorphisms and cancer susceptibility of breast cancer in Korean women. J Biochem Mol Biol. 2003;36:28–34. PMID: 12542972
34. Zhu J, Chang P, Bondy ML, Sahin AA, Singletary SE, Takahashi S, et al. Detection of 2-amino-1-methyl-6-phenylimidazo[4,5-b]-pyridine-DNA adducts in normal breast tissues and risk of breast cancer. Cancer Epidemiol Biomarkers Prev. 2003;12:830–7. PMID: 14504191
35. Park SK, Kang D, Noh DY, Lee KM, Kim SU, Choi JY, et al. Reproductive factors, glutathione S-transferase M1 and T1 genetic polymorphism and breast cancer risk. Breast Cancer Res Treat. 2003;78:89–96. PMID: 12611461
36. Roodi N, Dupont WD, Moore JH, Parl FF. Association of homozygous wild-type glutathione S-transferase M1 genotype with increased breast cancer risk. Cancer Res. 2004;64:1233–6. PMID: 14973116
37. McCready D, Aronson KJ, Chu W, Fan W, Vesprini D, Narod SA. Breast tissue organochlorine levels and metabolic genotypes in relation to breast cancer risk Canada. Cancer Causes Control. 2004;15:399–418. https://doi.org/10.1023/B:CACO.0000027505.32564.c2 PMID: 15141140
38. Sarmanová J, Sůsová S, Gut I, Mrhalová M, Kodet R, Adámek J, et al. Breast cancer: role of polymorphisms in biotransformation enzymes. Eur J Hum Genet. 2004;12:848–54. https://doi.org/10.1038/sj.ejhg.5201249 PMID: 15280903
39. Gago-Dominguez M, Castelao JE, Sun CL, Van Den Berg D, Koh WP, Lee HP, et al. Marine n-3 fatty acid intake, glutathione S-transferase polymorphisms and breast cancer risk in post-menopausal Chinese women in Singapore. Carcinogenesis. 2004;25:2143–7. https://doi.org/10.1093/carcin/bgh230 PMID: 15256483
40. Egan KM, Cai Q, Shu XO, Jin F, Zhu TL, Dai Q, et al. Genetic polymorphisms in GSTM1, GSTP1, and GSTT1 and the risk for breast cancer: results from the Shanghai Breast Cancer Study and meta-analysis. Cancer Epidemiol Biomarkers Prev. 2004;13:197–204. PMID: 14973092
41. Park SK, Yim DS, Yoon KS, Choi IM, Choi JY, Yoo KY, et al. Combined effect of GSTM1, GSTT1, and COMT genotypes in individual breast cancer risk. Breast Cancer Res Treat. 2004;88:55–62. https://doi.org/10.1007/s10549-004-0745-x PMID: 15538046
42. Kim SU, Lee KM, Park SK, Yoo KY, Noh DY, Choe KJ, et al. Genetic polymorphism of glutathione S-transferase P1 and breast cancer risk. J Biochem Mol Biol. 2004;37:582–5. PMID: 15479622
43. Vogl FD, Taioli E, Maugard C, Zheng W, Pinto LF, Ambrosone C, et al. Glutathione S-transferases M1, T1, and

P1 and breast cancer: a pooled analysis. Cancer Epidemiol Biomarkers Prev. 2004;13:1473–9. PMID: 15342448
44. Medeiros R, Soares R, Vasconcelos A, Schmitt F, Lopes C. Glutathione S-transferase genotype GSTM1 as a predictor of elevated angiogenic phenotype in patients with early onset breast cancer. Angiogenesis. 2004;7:53–8. https://doi.org/10.1023/B:AGEN.0000037330.20121.d8 PMID: 15302996
45. van der Hel OL, Peeters PH, Hein DW, Doll MA, Grobbee DE, Ocké M, et al. GSTM1 null genotype, red meat consumption and breast cancer risk (The Netherlands). Cancer Causes Control. 2004;15:295–303. https://doi.org/10.1023/B:CACO.0000024255.16305.f4 PMID: 15090724
46. Linhares JJ, Da Silva ID, De Souza NC, Noronha EC, Ferraro O, De Carvalho CV, et al. Genetic polymorphism of GSTM1 in women with breast cancer and interact with reproductive history and several clinical pathologies. Biol Res. 2005;38:273–81. PMID: 16238106
47. van der Hel OL, Bueno-de-Mesquita HB, van Gils CH, Roest M, Slothouber B, Grobbee DE, et al. Cumulative genetic defects in carcinogen metabolism may increase breast cancer risk (The Netherlands). Cancer Causes Control. 2005;16:675–81. https://doi.org/10.1007/s10552-005-1227-0 PMID: 16049806
48. Ceschi M, Sun CL, Van Den Berg D, Koh WP, Yu MC, Probst-Hensch N. The effect of cyclin D1 (CCND1) G870A-polymorphism on breast cancer risk is modified by oxidative stress among Chinese women in Singapore. Carcinogenesis. 2005;26:1457–64. https://doi.org/10.1093/carcin/bgi093 PMID: 15845652
49. Chacko P, Joseph T, Mathew BS, Rajan B, Pillai MR. Role of xenobiotic metabolizing gene polymorphisms in breast cancer susceptibility and treatment outcome. Mutat Res. 2005;581:153–63. https://doi.org/10.1016/j.mrgentox.2004.11.018 PMID: 15725614
50. Cheng TC, Chen ST, Huang CS, Fu YP, Yu JC, Cheng CW, et al. Breast cancer risk associated with genotype polymorphism of the catechol estrogen-metabolizing genes: a multigenic study on cancer susceptibility. Int J Cancer. 2005;113:345–53. https://doi.org/10.1002/ijc.20630 PMID: 15455371
51. Wu SH, Tsai SM, Hou MF, Lin HS, Hou LA, Ma H, et al. Interaction of genetic polymorphisms in cytochrome P450 2E1 and glutathione S-transferase M1 to breast cancer in Taiwanese woman without smoking and drinking habits. Breast Cancer Res Treat. 2006;100:93–8. https://doi.org/10.1007/s10549-006-9226-8 PMID: 16758119
52. Chang TW, Wang SM, Guo YL, Tsai PC, Huang CJ, Huang W. Glutathione S-transferase polymorphisms associated with risk of breast cancer in southern Taiwan. Breast. 2006;15:754–61. https://doi.org/10.1016/j.breast.2006.03.008 PMID: 16713266
53. Onay VU, Briollais L, Knight JA, Shi E, Wang Y, Wells S, et al. SNP-SNP interactions in breast cancer susceptibility. BMC Cancer. 2006;6:114. https://doi.org/10.1186/1471-2407-6-114 PMID: 16672066
54. Samson M, Swaminathan R, Rama R, Sridevi V, Nancy KN, Rajkumar T. Role of GSTM1 (Null/Present), GSTP1 (Ile105Val) and P53 (Arg72Pro) genetic polymorphisms and the risk of breast cancer: a case control study from South India. Asian Pac J Cancer Prev. 2007;8:253–7. PMID: 17696741
55. Steck SE, Gaudet MM, Britton JA, Teitelbaum SL, Terry MB, Neugut AI, et al. Interactions among GSTM1, GSTT1 and GSTP1 polymorphisms, cruciferous vegetable intake and breast cancer risk. Carcinogenesis. 2007;28:1954–9. https://doi.org/10.1093/carcin/bgm141 PMID: 17693660
56. Spurdle AB, Chang JH, Byrnes GB, Chen X, Dite GS, McCredie MR, et al. A systematic approach to analysing gene-gene interactions: polymorphisms at the microsomal epoxide hydrolase EPHX and glutathione S-transferase GSTM1, GSTT1, and GSTP1 loci and breast cancer risk. Cancer Epidemiol Biomarkers Prev. 2007;16:769–74. https://doi.org/10.1158/1055-9965.EPI-06-0776 PMID: 17416769
57. Edvardsen H, Kristensen VN, Grenaker Alnaes GI, Bøhn M, Erikstein B, Helland A, et al. Germline glutathione S-transferase variants in breast cancer: relation to diagnosis and cutaneous long-term adverse effects after two fractionation patterns of radiotherapy. Int J Radiat Oncol Biol Phys. 2007;67:1163–71. https://doi.org/10.1016/j.ijrobp.2006.11.009 PMID: 17336217
58. Nordgard SH, Ritchie MD, Jensrud SD, Motsinger AA, Alnaes GI, Lemmon G, et al. ABCB1 and GST polymorphisms associated with TP53 status in breast cancer. Pharmacogenet Genomics. 2007;17:127–36. https://doi.org/10.1097/FPC.0b013e328011abaa PMID: 17301692
59. Justenhoven C, Hamann U, Schubert F, Zapatka M, Pierl CB, Rabstein S, et al. Breast cancer: a candidate gene approach across the estrogen metabolic pathway. Breast Cancer Res Treat. 2008;108:137–49. https://doi.org/10.1007/s10549-007-9586-8 PMID: 17588204
60. Torresan C, Oliveira MM, Torrezan GT, de Oliveira SF, Abuázar CS, Losi-Guembarovski R, et al. Genetic polymorphisms in oestrogen metabolic pathway and breast cancer: a positive association with combined CYP/GST genotypes. Clin Exp Med. 2008;8:65–71. https://doi.org/10.1007/s10238-008-0159-x PMID: 18618215
61. Kadouri L, Kote-Jarai Z, Hubert A, Baras M, Abeliovich D, Hamburger T, et al. Glutathione-S-transferase M1, T1 and P1 polymorphisms, and breast cancer risk, in BRCA1/2 mutation carriers. Br J Cancer. 2008;98:2006–10. https://doi.org/10.1038/sj.bjc.6604394 PMID: 18542066
62. Van Emburgh BO, Hu JJ, Levine EA, Mosley LJ, Perrier ND, Freimanis RI, et al. Polymorphisms in CYP1B1,

GSTM1, GSTT1 and GSTP1, and susceptibility to breast cancer. Oncol Rep. 2008;19:1311–21. PMID: 18425393
63. Syamala VS, Sreeja L, Syamala V, Raveendran PB, Balakrishnan R, Kuttan R, et al. Influence of germline polymorphisms of GSTT1, GSTM1, and GSTP1 in familial versus sporadic breast cancer susceptibility and survival. Fam Cancer. 2008;7:213–20. https://doi.org/10.1007/s10689-007-9177-1 PMID: 18080216
64. Rajkumar T, Samson M, Rama R, Sridevi V, Mahji U, Swaminathan R, et al. TGFbeta1 (Leu10Pro), p53 (Arg72Pro) can predict for increased risk for breast cancer in south Indian women and TGFbeta1 Pro (Leu10Pro) allele predicts response to neo-adjuvant chemo-radiotherapy. Breast Cancer Res Treat. 2008;112:81–7. https://doi.org/ 10.1007/s10549-007-9821-3 PMID: 18058229
65. Sakoda LC, Blackston CR, Xue K, Doherty JA, Ray RM, Lin MG, et al. Glutathione S-transferase M1 and P1 polymorphisms and risk of breast cancer and fibrocystic breast conditions in Chinese women. Breast Cancer Res Treat. 2008;109:143–55. https://doi.org/10.1007/s10549-007-9633-5 PMID:17624589
66. Lee SA, Fowke JH, Lu W, Ye C, Zheng Y, Cai Q, et al. Cruciferous vegetables, the GSTP1 Ile105Val genetic polymorphism, and breast cancer risk. Am J Clin Nutr. 2008;87:753–60. https://doi.org/10.1093/ajcn/87.3.753 PMID: 18326615
67. Unlü A, Ates NA, Tamer L, Ates C. Relation of glutathione S-transferase T1, M1 and P1 genotypes and breast cancer risk. Cell Biochem Funct. 2008;26:643–7. https://doi.org/10.1002/cbf.1490 PMID: 18521819
68. Kostrykina NA, Pechkovskii EV, Mishukova OV, Khripko UI, Zarubina NA, Selezneva IA, et al. Studying the association of polymorphic variants of GSTM1 and GSTT1 genes with breast cancer in female residents of Altai Krai. Bull Exp Biol Med. 2009;148:89–93. PMID: 19902106
69. McCarty KM, Santella RM, Steck SE, Cleveland RJ, Ahn J, Ambrosone CB, et al. PAH-DNA adducts, cigarette smoking, GST polymorphisms, and breast cancer risk. Environ Health Perspect. 2009;117:552–8. https://doi.org/10.1289/ehp.0800119 PMID: 19440493
70. Reding KW, Weiss NS, Chen C, Li CI, Carlson CS, Wilkerson HW, et al. Genetic polymorphisms in the catechol estrogen metabolism pathway and breast cancer risk. Cancer Epidemiol Biomarkers Prev. 2009;18:1461–7. https://doi.org/10.1158/1055-9965.EPI-08-0917 PMID: 19383894
71. Yu KD, Di GH, Fan L, Wu J, Hu Z, Shen ZZ, et al. A functional polymorphism in the promoter region of GSTM1 implies a complex role for GSTM1 in breast cancer. FASEB J. 2009;23:2274–87. https://doi.org/10.1096/fj.08-124073 PMID: 19228880
72. Saxena A, Dhillon VS, Raish M, Asim M, Rehman S, Shukla NK, et al. Detection and relevance of germline genetic polymorphisms in glutathione S-transferases (GSTs) in breast cancer patients from northern Indian population. Breast Cancer Res Treat. 2009;115:537–43. https://doi.org/10.1007/s10549-008-0098-y PMID: 18574688
73. Antognelli C, Del Buono C, Ludovini V, Gori S, Talesa VN, Crinò L, et al. CYP17, GSTP1, PON1 and GLO1 gene polymorphisms as risk factors for breast cancer: an Italian case–control study. BMC Cancer. 2009;9:115. https://doi.org/10.1186/1471-2407-9-115 PMID: 19379515
74. Pongtheerat T, Treetrisool M, Purisa W. Glutathione s-transferase polymorphisms in breast cancers of Thai patients. Asian Pac J Cancer Prev. 2009;10:127–32. PMID: 19469640
75. Kaushal M, Mishra AK, Raju BS, Ihsan R, Chakraborty A, Sharma J, et al. Betel quid chewing as an environmental risk factor for breast cancer. Mutat Res. 2010;703:143–8. https://doi.org/10.1016/j.mrgentox.2010.08.011 PMID: 20728566
76. Yu KD, Fan L, Di GH, Yuan WT, Zheng Y, Huang W, et al. Genetic variants in GSTM3 gene within GSTM4-GSTM2-GSTM1-GSTM5-GSTM3 cluster influence breast cancer susceptibility depending on GSTM1. Breast Cancer Res Treat. 2010;121:485–96. https://doi.org/10.1007/s10549-009-0585-9 PMID: 19856098
77. Masoudi M, Saadat I, Omidvari S, Saadat M. Additive effects of genetic variations of xenobiotic detoxification enzymes and DNA repair gene XRCC1 on the susceptibility to breast cancer. Breast Cancer Res Treat. 2010;120:263–5. https://doi.org/10.1007/s10549-009-0521-z PMID: 19731014
78. MARIE-GENICA Consortium on Genetic Susceptibility for Menopausal Hormone Therapy Related Breast Cancer Risk, Chang-Claude J, Beckmann L, Corson C, Hein R, et al. Genetic polymorphisms in phase I and phase II enzymes and breast cancer risk associated with menopausal hormone therapy in postmenopausal women. Breast Cancer Res Treat. 2010;119:463–74. https://doi.org/10.1007/s10549-009-0407-0 PMID: 19424794
79. Delort L, Satih S, Kwiatkowski F, Bignon YJ, Bernard-Gallon DJ. Evaluation of breast cancer risk in a multigenic model including low penetrance genes involved in xenobiotic and estrogen metabolisms. Nutr Cancer. 2010;62:243–51. https://doi.org/10.1080/01635580903305300 PMID: 20099199
80. Sangrajrang S, Sato Y, Sakamoto H, Ohnami S, Khuhaprema T, Yoshida T. Genetic polymorphisms in folate and alcohol metabolism and breast cancer risk: a case-control study in Thai women. Breast Cancer Res Treat. 2010;123:885–93. https://doi.org/10.1007/s10549-010-0804-4 PMID: 20180013
81. Nosheen M, Malik FA, Kayani MA. Lack of influence of glutathione S-transferase gene deletions in sporadic

breast cancer in Pakistan. Asian Pac J Cancer Prev. 2011;12:1749–52. PMID: 22126558
82. Ramalhinho AC, Fonseca-Moutinho JA, Breitenfeld L. Glutathione S-transferase M1, T1, and P1 genotypes and breast cancer risk: a study in a Portuguese population. Mol Cell Biochem. 2011;355:265–71. https://doi.org/10.1007/s11010-011-0863-9 PMID: 21559761
83. Cribb AE, Joy Knight M, Guernsey J, Dryer D, Hender K, Shawwa A, et al. CYP17, catechol-o-methyltransferase, and glutathione transferase M1 genetic polymorphisms, lifestyle factors, and breast cancer risk in women on Prince Edward Island. Breast J. 2011;17:24–31. https://doi.org/10.1111/j.1524-4741.2010.01025.x PMID:21129090
84. Naushad SM, Reddy CA, Rupasree Y, Pavani A, Digumarti RR, Gottumukkala SR, et al. Cross-talk between one-carbon metabolism and xenobiotic metabolism: implications on oxidative DNA damage and susceptibility to breast cancer. Cell Biochem Biophys. 2011;61:715–23. https://doi.org/10.1007/s12013-011-9245-x PMID: 21792634
85. Cerne JZ, Pohar-Perme M, Novakovic S, Frkovic-Grazio S, Stegel V, Gersak K. Combined effect of CYP1B1, COMT, GSTP1, and MnSOD genotypes and risk of postmenopausal breast cancer. J Gynecol Oncol. 2011;22:110–9. https://doi.org/10.3802/jgo.2011.22.2.110 PMID: 21860737
86. Reding KW, Chen C, Lowe K, Doody DR, Carlson CS, Chen CT, et al. Estrogen-related genes and their contribution to racial differences in breast cancer risk. Cancer Causes Control. 2012;23:671–81. https://doi.org/10.1007/s10552-012-9925-x PMID: 22418777
87. Hashemi M, Eskandari-Nasab E, Fazaeli A, Taheri M, Rezaei H, Mashhadi M, et al. Association between polymorphisms of glutathione S-transferase genes (GSTM1, GSTP1 and GSTT1) and breast cancer risk in a sample Iranian population. Biomark Med. 2012;6:797–803. https://doi.org/10.2217/bmm.12.61 PMID: 23227845
88. Ramalhinho AC, Fonseca-Moutinho JA, Breitenfeld Granadeiro LA. Positive association of polymorphisms in estrogen biosynthesis gene, CYP19A1, and metabolism, GST, in breast cancer susceptibility. DNA Cell Biol. 2012;31:1100–6. https://doi.org/10.1089/dna.2011.1538 PMID: 22300440
89. Luo J, Gao YT, Chow WH, Shu XO, Li H, Yang G, et al. Urinary polyphenols, glutathione S-transferases copy number variation, and breast cancer risk: results from the Shanghai women's health study. Mol Carcinog. 2012;51:379–88. https://doi.org/10.1002/mc.20799 PMID: 21557334
90. Saxena A, Dhillon VS, Shahid M, Khalil HS, Rani M, Prasad DAS T, et al. GSTP1 methylation and polymorphism increase the risk of breast cancer and the effects of diet and lifestyle in breast cancer patients. Exp Ther Med. 2012;4:1097–1103. https://doi.org/10.3892/etm.2012.710 PMID: 23226781
91. McCullough LE, Santella RM, Cleveland RJ, Bradshaw PT, Millikan RC, North KE, et al. Polymorphisms in oxidative stress genes, physical activity, and breast cancer risk. Cancer Causes Control. 2012;23:1949–58. https://doi.org/10.1007/s10552-012-0072-1 PMID: 23053794
92. Sohail A, Kanwal N, Ali M, Sadia S, Masood AI, Ali F, et al. Effects of glutathione-S-transferase polymorphisms on the risk of breast cancer: a population-based case–control study in Pakistan. Environ Toxicol Pharmacol. 2013;35:143–53. https://doi.org/10.1016/j.etap.2012.11.014 PMID: 23376175
93. Zgheib NK, Shamseddine AA, Geryess E, Tfayli A, Bazarbachi A, Salem Z, et al. Genetic polymorphisms of CYP2E1, GST, and NAT2 enzymes are not associated with risk of breast cancer in a sample of Lebanese women. Mutat Res. 2013;747–748:40–7. https://doi.org/10.1016/j.mrfmmm.2013.04.004 PMID: 23628324
94. Possuelo LG, Peraça CF, Eisenhardt MF, Dotto ML, Cappelletti L, Foletto E, et al. Polymorphisms of GSTM1 and GSTT1 genes in breast cancer susceptibility: a case–control study. Rev Bras Ginecol Obstet. 2013;35:569–74. PMID: 24500512
95. Ge J, Tian AX, Wang QS, Kong PZ, Yu Y, Li XQ, et al. The GSTP1 105Val allele increases breast cancer risk and aggressiveness but enhances response to cyclophosphamide chemotherapy in North China. PLoS One. 2013;8:e67589. https://doi.org/10.1371/journal.pone.0067589 PMID: 23826324
96. Chirilă DN, Bălăcescu O, Popp R, Oprea A, Constantea NA, Vesa S, et al. GSTM1, GSTT1 and GSTP1 in patients with multiple breast cancers and breast cancer in association with another type of cancer. Chirurgia (Bucur). 2014;109:626–33. PMID: 25375048
97. Li JY, Long QM, Tao P, Hu R, Li H, Lei FM, et al. Using MSR model to analyze the impact of gene-gene interaction with related to the genetic polymorphism of metabolism enzymes on the risk of breast cancer. Sichuan Da Xue Xue Bao Yi Xue Ban 2008;39:780–783. PMID: 19024313
98. Khabaz MN. Polymorphism of the glutathione S-transferase P1 gene (GST-pi) in breast carcinoma. Pol J Pathol. 2014;65:141–6. PMID: 25119175
99. Khabaz MN, Gari MA, Al-Maghrabi JA, Nedjadi T, Bakarman M. Association between GSTP1 genotypes and hormone receptor phenotype in invasive ductal carcinomas of breast. Asian Pac J Cancer Prev. 2015;16:1707–13. PMID: 25773813
100. Soto-Quintana O, Zúñiga-González GM, Ramírez-Patiño R, Ramos-Silva A, Figuera LE, Carrillo-Moreno DI, et

al. Association of the GSTM1 null polymorphism with breast cancer in a Mexican population. Genet Mol Res. 2015;14:13066–75. https://doi.org/10.4238/2015.October.26.2 PMID: 26535619
101. Jaramillo-Rangel G, Ortega-Martínez M, Cerda-Flores RM, Barrera-Saldaña HA. Polymorphisms in GSTM1, GSTT1, GSTP1, and GSTM3 genes and breast cancer risk in northeastern Mexico. Genet Mol Res. 2015;14:6465–71. https://doi.org/10.4238/2015.June.11.22 PMID: 26125851
102. Kimi L, Ghatak S, Yadav RP, Chhuani L, Lallawmzuali D, Pautu JL, et al. Relevance of GSTM1, GSTT1 and GSTP1 Gene Polymorphism to Breast Cancer Susceptibility in Mizoram Population, Northeast India. Biochem Genet. 2016;54:41–9. https://doi.org/10.1007/s10528-015-9698-5 PMID: 26407578
103. García-Martínez A, Gamboa-Loira B, Tejero ME, Sierra-Santoyo A, Cebrián ME, López-Carrillo L. CYP1A1, CYP1B1, GSTM1 and GSTT1 genetic variants and breast cancer risk in Mexican women. Salud Publica Mex. 2017;59:540–7. https://doi.org/10.21149/8527 PMID: 29267651
104. Wang X, Jihu Q, Wang H. Relationship Between GSTM1 Gene Deletion and Susceptibility to Breast Cancer. Acta Academ e Medicinae Nantong. 2002;22:11–2.
105. Cui Z, Qian BY, Chen KX, Zheng F, Ma J. The Relationship of CYP1A1, GSTT1 and GSTM1 with Susceptibility of Breast Cancer Based on a Case–Control study. Chin J Prev Control Chron Dis. 2010;18: 249–52.
106. Fan B, Tang JH, Wu JZ, Wang ZH, Xue L. A preliminary study on the relationship between genetic polymorphism of Glutathione-S transferases T1, M1 genes susceptibility of young female breast cancer in Nanjing area. Prog Mod Biomed. 2012;12:4813–5.
107. Ma J, Cui Z, Sun Z, Qian B, Chen K. A case–control study on the association between polymorphism of GSTT1, GSTM1 and susceptibility for breast cancer. Chin J Prev Contr Chron. 2007;15:123–6.
108. Li J, Chang YL, Jiang SF, Guo Z, Yao SQ, Hu WN, et al. Relationship between serum organochlorine residues genetic polymorphism of glutathione S-transferase M1 and breast cancer in women. Chin J Public Health. 2010;26:558–60.
109. Li SF. A case–control study on the association between polymorphism of GSTM1, GSTT1 and susceptibility to breast cancer and lung cancer. The Master Thesis of Sichuan University. 2007.
110. Harries LW, Stubbins MJ, Forman D, Howard GC, Wolf CR. Identification of genetic polymorphisms at the glutathione S-transferase Pi locus and association with susceptibility to bladder, testicular and prostate cancer. Carcinogenesis. 1997;18:641–644. PMID: 9111193
111. Sgambato A, Campisi B, Zupa A. Glutathione S-transferase (GST) polymorphisms as risk factors for cancer in a highly homogeneous population from southern Italy. Anticancer Res. 2002;22:3647–52. PMID: 12552971
112. Ermolenko NA, Boiarskikh UA, Sushko AG, Voronina EN, Selezneva IA, Sinkina TV, et al. [Effect of point substitutions in the MnSOD, GPX1, and GSTP1 genes on the risk of familial and sporadic breast cancers in residents of the Altaĭ region of the Russian Federation]. Genetika. 2010;46:1685–91. PMID: 21434422
113. Morais LM, Cardoso Filho C, Lourenço GJ, Shinzato JY, Zeferino LC, Lima CS, et al. [Polymorphisms GSTM1 and GSTT1 and sporadic breast cancer mammographic features]. Rev Assoc Med Bras. 1992;2008;54:61–6. PMID: 18392488
114. Chang YL, Li J, Yao SQ, Hu WN, Jiang SF, Guo Z, et al. [A case–control study on serum organochlorines residues, genetic polymorphisms of glutathione S-transferase T1 and the risks of breast cancer]. Zhonghua Liu Xing Bing Xue Za Zhi. 2008;29:763–6. PMID: 19103108
115. Cui Z, Ma J, Qian BY, Zheng F, Chen KX. Case–Control Study on Polymorphism of GSTT1 and GSTM1 in Breast Cancer. Tianjin Med J. 2007;35:284–6.
116. Kong Z. The association of glutathione S-transferase P1 gene polymorphism with the susceptibility of breast cancer in GuangXi. The Master Thesis of Sichuan University. 2016.
